# An integrative genomic analysis revealed the relevance of microRNA and gene expression for drug-resistance in human breast cancer cells

**DOI:** 10.1186/1476-4598-10-135

**Published:** 2011-11-03

**Authors:** Yusuke Yamamoto, Yusuke Yoshioka, Kaho Minoura, Ryou-u Takahashi, Fumitaka Takeshita, Toshiki Taya, Reiko Horii, Yayoi Fukuoka, Takashi Kato, Nobuyoshi Kosaka, Takahiro Ochiya

**Affiliations:** 1 Division of Molecular and Cellular Medicine, National Cancer Center Research Institute, 1-1, Tsukiji, 5-chome, Chuo-ku, Tokyo 104-0045, Japan; 2 Major in Integrative Bioscience and Biomedical Engineering, Graduate School of Science and Engineering, Waseda University, Nishi-waseda 1-6-1, Shinjuku-ku, Tokyo, 169-8050, Japan; 3 Agilent Technologies Japan Ltd., 9-1, Takakura-cho, Hachioji-shi, Tokyo, 192-8510, Japan; 4 Research Fellow of the Japan Society for the Promotion of Science (JSPS) 8 Ichibancho, Chiyoda-ku, Tokyo 102-8472, Japan

**Keywords:** aCGH, microRNA, gene expression, breast cancer, drug resistance

## Abstract

**Background:**

Acquisition of drug-resistance in cancer has led to treatment failure, however, their mechanisms have not been clarified yet. Recent observations indicated that aberrant expressed microRNA (miRNA) caused by chromosomal alterations play a critical role in the initiation and progression of cancer. Here, we performed an integrated genomic analysis combined with array-based comparative hybridization, miRNA, and gene expression microarray to elucidate the mechanism of drug-resistance.

**Results:**

Through genomic approaches in MCF7-ADR; a drug-resistant breast cancer cell line, our results reflect the unique features of drug-resistance, including MDR1 overexpression via genomic amplification and miRNA-mediated TP53INP1 down-regulation. Using a gain of function study with 12 miRNAs whose expressions were down-regulated and genome regions were deleted, we show that miR-505 is a novel tumor suppressive miRNA and inhibits cell proliferation by inducing apoptosis. We also find that Akt3, correlate inversely with miR-505, modulates drug sensitivity in MCF7-ADR.

**Conclusion:**

These findings indicate that various genes and miRNAs orchestrate to temper the drug-resistance in cancer cells, and thus acquisition of drug-resistance is intricately controlled by genomic status, gene and miRNA expression changes.

## Background

Systemic therapy improves disease-free survival in patients with breast cancer, but does not cure patients with advanced or metastatic disease, and fails to benefit the majority of patients with localized breast cancer. Intrinsic resistance to chemotherapy is emerging as a significant cause of treatment failure, and evolving research has identified several potential causes of resistance [1]. For instance, P-glycoprotein (Pgp), the drug efflux pump encoded by the MDR-1 gene is associated with multidrug resistance in several kinds of advanced cancer. Furthermore, the multidrug resistance-associated protein MRP1 [2,3], breast cancer resistance protein (ABCG2) and other transporters [4], which act as energy-dependent efflux pumps capable of expelling a large range of xenobiotics, have been reported to be upregulated in tumor cells showing the multidrug-resistant phenotype. In addition, overexpression of anti-apoptotic proteins, such as Bcl-2 and Bcl-xL, are also associated with drug resistance and poor clinical outcome in cancer patients. It is essential to decide the molecular target to treat the advanced cancer by molecular targeted therapies such as RNA interference and antibody treatment, however, regulatory networks underlying drug resistance in cancer cells have been elusive.

MicroRNAs (miRNAs) are small non-coding RNA of 21-25nt transcripts, playing central roles in physiological and pathological processes, including cell differentiation, apoptosis, and oncogenesis by either inducing mRNA degradation or by regulating the translational efficiency of mRNA [5-7]. Recently, several research groups have provided evidence that some miRNA expression levels are frequently modulated by genomic aberrations, such as genomic DNA copy number gain or loss, translocations, and epigenetic regulations [8]. For example, miR-15a and miR-16-1, whose genomic regions are deleted and expressions are down-regulated in the majority of chronic lymphocytic leukemia (CLL). Furthermore, their target Bcl-2 is overexpressed in CLL at the mRNA and protein level [9]. Another study showed that the expression level of miR-34a was down-regulated by deletion of 1p36 heterozygosity in neuroblastoma and contributed to an aggressive phenotype [10]. As reported in the studies of cancer genetics in lung, leukemia, colon, breast and ovary, a large number of miRNAs are located at chromosomal fragile sites, i.e., minimal regions of loss of heterozygosity (LOH) and minimal regions of genomic amplification [11]. These reports indicated that the emphasis on a genomic analysis was due to the fact that DNA copy number alterations are associated with expression levels of miRNAs and genes.

In this study, to better understand the regulatory network underlying drug resistance in breast cancer cells, we focus on miRNAs and genes located on the genome-amplified and -deleted regions because genomic aberration is closely associated with gene expression, and this expression alteration might be constantly maintained. For the identification of molecular targets, we initially performed an integrated genomic analysis to compare the DNA copy number and expression profile of mRNA and miRNA between MCF7; a parental breast cancer cell line and MCF7-ADR; a drug-resistant breast cancer cell line [12,13]. Through the genomic analysis, we found that the genomic alterations of drug resistance-related genes, e.g. amplified genomic regions and overexpression of MDR-1 and miRNA-mediated TP53INP1 down-regulation. In addition, of 12 miRNAs whose expressions were down-regulated and genomic regions were deleted, we determined that miR-505 promotes the inhibition of cell growth in MCF7-ADR cells, by inducing apoptotic cell death in the presence of docetaxel (DOC).

## Methods

### Cell culture

MCF7 human mammary carcinoma cells and multidrug-resistant MCF7-ADR human mammary carcinoma cells were obtained from Shien-Lab, Medical Oncology, National Cancer Center Hospital. MCF7-ADR-Luc cells were established by transfecting with a pLuc-neo expression vector, which has the firefly luciferase GL3 cDNA cloned into the downstream of the SV40 promoter and the G418 selective marker gene. Cells were selected in a medium containing 0.6 mg/ml of G418 (Gibco BRL) and were maintained and passaged in an RPMI 1640 medium (Gibco BRL) supplemented with 10% fetal bovine serum (Gibco BRL) under 5% CO_2 _in a humidified incubator at 37°C.

### RNA and genomic DNA extraction

Total RNA was extracted from MCF7, MCF7-ADR, and MCF7-ADR-Luc cells using the ISOGEN solution (Nippon Gene, Tokyo, Japan) according to the manufacturer's protocol. Genomic DNA was prepared from MCF7 and MCF7-ADR. The yield and purity of the genomic DNA and total RNA were measured using a NanoDrop ND-1000 spectrophotometer (Thermo Fisher Scientific). The quality of the total RNA was verified to have an RNA Integrity Number using a Bioanalyzer and RNA 6000 LabChip Kit (Agilent Technologies).

### Oligonucleotide array CGH (aCGH) Analysis

All DNA labeling reactions and hybridizations were carried out following the manufacture's protocol (Agilent Oligonucleotide Array-Based CGH for Genomic DNA Analysis, Version 4.0, Direct Method). Briefly, 3.0 μg of MCF-7, MCF7-ADR and reference DNA (Promega, female, p/n G1521) were digested with *Alu*I and *Rsa*I for 2 hours at 37°C, followed by heat inactivation at 65°C for 10 minutes. Digested DNA was then labeled using the Agilent Genomic DNA Labeling Kit Plus (p/n 5188-5309) using random primers and the exo-Klenow fragment to differentially label genomic DNA samples with fluorescently labeled nucleotides. All experimental and reference samples were labeled with Cyanine-5 dUTP and Cyanine-3 dUTP, separately, for 2 hours at 37°C to enable duplicate hybridizations with the corresponding dye reversal arrays. Experimental and reference targets for each hybridization were purified with a Microcon YM-30 column (Millipore) and validated by the NanoDrop ND-1000, respectively to ensure the yield and the specific dye incorporation activity of the labeled genomic DNA. The individual pair of labeled targets were combined together, mixed with Cot-1 DNA (Invitrogen) and 10xBlocking Agent (Agilent), and then mixed with Agilent 2xHybridization Buffer (p/n 5188-5220). Before hybridization, the combined mixtures were denatured for 3 minutes at 95°C, incubated for 30 minutes at 37°C and then applied to the Agilent Human 244A CGH arrays (G4411B). Using an Agilent microarray hybridization chambers, the hybridization was carried out for 40 hours at 65°C in a rotating oven (Agilent) at 20 r.p.m. The hybridization chambers were then disassembled and array slides were washed for 5 minutes at room temperature in Agilent Oligo aCGH Wash Buffers 1, followed by 1 minute at 37°C in Agilent Oligo aCGH Wash Buffer 2 (p/n 5188-5226) (prewarmed to 37°C overnight). The slides were removed from the wash buffer 2 slowly (5-10 seconds) after which time they were completely dry and were scanned using an Agilent DNA Microarray scanner with 5 μm resolution. The data of microarray images were extracted by Agilent Feature Extraction Software v9.5 in which a modified Feature Extraction protocol, CGH-v4_95_Feb07 was used in conjunction with a gene list on chromosome 21 q-arm to normalize spot-intensity values and ratios to each extraction set. These ratio data along with associated error values and flagged features were imported into CGH Analytics Software v3.4 (Agilent). The dye reversal data and intra-replicate spots were then combined while the data centralization and fuzzy zero algorithms were not applied in the CGH Analytics. To make aberration calls, an aberration detection algorithm, ADM-2 [14] was used at threshold 10 and an aberration filter was set at 2 for the minimum number of probe region and 1 for minimum absolute average log2 ratio for regions in the CGH Analytics to reduce false positives.

### Gene Expression Analysis

All RNA labeling reactions and hybridizations were carried out following the manufacture's protocol (Agilent One-Color Microarray-Based Gene Expression Analysis, Version 5.0.1). Briefly, polyA(+)RNA in 500 ng of total RNA was primed with an oligo (d)T-T7 primer and converted into dsDNA with MMLV-RT, then transcribed and simultaneously labeled with Cyanine 3-CTP for 2 hours at 40°C using Agilent Low RNA Input Linear Amplification Kit (p/n 5188-5339). After labeling and cRNA purification, cRNA was quantified and the specific dye incorporation activity was validated using the NanoDrop ND-1000. 1.65 μg of labeled cRNA was mixed with Agilent 10×Blocking Agent and 25×Fragmentation Buffer, then incubated at 60°C for 30 hours. After fragmentation, the cRNA mixtures were immediately mixed with Agilent 2×Hybridization Buffer (p/n 5188-5339) and applied to the Agilent Human 4×44 K whole genome microarrays (G4112F) for 17 hours at 65°C (10 r.p.m.). Array slides were washed with Agilent Gene Expression Wash Buffer 1 and 2 (p/n 5188-5327) and then scanned using the Agilent DNA Microarray scanner with 5 μm resolution and the eXtended Dynamic range setting (XDR Hi 100%, Low 10%) to avoid saturated features. The data of microarray images were extracted by Agilent Feature Extraction Software v9.5 using the GE1_v5_95 protocol. The extracted signal intensities and flagged information were imported into GeneSpring 7.3.1 software and the data sets were normalized by adjusting the intensity distribution of well-above background and unflagged features to 50th percentile to account for the interchip variability. Comparison of MCF7 and MCF7-ADR was done using duplicate array data set for each cell line.

### miRNA Expression Analysis

All RNA labeling reactions and hybridizations were carried out following the manufacture's prototype protocol (Agilent miRNA Microarray system, Version 0.3, early access). Briefly, 100 ng of total RNA including fraction of small mature miRNA was dephosphorylated by calf intestine alkaline phosphatase (p/n E2250Y, Amersham Biosciences) for 30 minutes at 37°C and denatured by adding DMSO (p/n D8418, Sigma) for 8 minutes at 100°C. Ligation was then carried out with T4 RNA ligase (p/n E2050Y, Amersham Biosciences) and pCp-Cy3 (p/n 5190-0408, Agilent) for 2 hours at 16°C that allowed us to perform a quantitative direct labeling method [15]. The labeled miRNAs were desalted with Micro Bio-Spin 6 column (p/n 732-6221, Bio-Rad) and combined with Agilent 10×GE Blocking Agent and 2×Hybridization Buffer (p/n 5190-0408). The mixture was heated for 5 minutes at 100°C and immediately cooled to 0°C. Each sample was hybridized to the Agilent early access Human 8×15 K microRNA microarrays covered 470 miRNAs (AMADID 015508, early access) for 20 hours at 55°C (20 r.p.m.). Array slides were washed with 6× SSC/0.005% Triton X-100 for 10 minutes, then 0.1× SSC/0.005% Triton X-100 for 5 minutes, both at room temperature. Slides were scanned using the Agilent DNA Microarray scanner with 5 μm resolution and the eXtended Dynamic range setting (XDR Hi 100%, Low 5%) to avoid saturated features. The data were extracted by Agilent Feature Extraction Software v9.5 using the miRNA_120106 protocol which extracts intensities of multiple probes with multiple features per probe and reports the measurements and errors as the TotalGeneSignal and TotalGeneSignalError for each of the miRNAs. These values were imported to the GeneSpring GX version 7.3.1 without applying any normalization algorithm. The miRNA profiles generated on the Agilent platform were prior normalized to the amount of input total RNA in which 100 ng of total RNA were equally used for each assay and all of the labeled targets were loaded on each array. Comparison of MCF7 and MCF7-ADR was done using duplicate array data set for each cell line.

### Transfection of miRNA into MCF7-ADR-Luc cells

For MCF7-ADR-Luc cells, transfection of miRNA was carried out using DharmaFECT 1 (Dharmacon) according to the manufacturer's protocol. MCF7-ADR-Luc cells were plated in growth medium 24 hours before transfection. The cells, which were grown to 50% confluence, were transfected with 20 nM miRNAs and cultured. Two or 3 days after transfection, the cells were subjected to further analyses.

### Apoptosis assay measurement of caspase activity *in vitro*

Caspase-7, which plays key effecter roles in apoptosis, was detected in caspase-3-deficient MCF7-ADR-Luc cells. The arrays used in an *in vitro *growth assay were measured with the Apo-ONE Homogeneous Caspase-3/7 Assay (Promega) according to the manufacturer's instructions. Cells were incubated with the Apo-ONE Caspase-3/7 Assay Reagent for 1.5 hours at room temperature, and the fluorescence was then measured at 485Ex/535Em with a Wallac Multi-label Counter.

### Hoechst staining

Cells were washed with PBS(-), and a fixative and staining solution was added (4% paraformaldehyde, 1 μg/ml Hoechst 33342 in PBS). Ten minutes after incubation, cells were washed with PBS, and the number of apoptotic cells was then determined in three microscopic fields of each well by fluorescence microscopy (Olympus).

### Luciferase assay for the measurement of cell growth

MCF7-ADR-Luc cells were plated into 5 × 10^3 ^cells per well and cultured. The cell growth in each well was then estimated by firefly luciferase activity because the cell numbers were correlated with the bioluminescence from MCF7-ADR-Luc cells. Luciferase assays were performed with a Wallac Multi-label Counter (PerkinElmer) and Bright-Glo Luciferase Assay System (Promega, Tokyo, Japan) according to the manufacturer's protocol.

### Real-time RT-PCR

The total RNA was used to produce cDNAs with the SuperScript™ II First-Strand Synthesis System (Invitrogen, Tokyo, Japan) according to the manufacturer's protocol. For quantification, cDNA samples were subjected to real-time PCR using Platinum SYBR Green qPCR SuperMix UDG (Invitrogen) in triplicates, and reactions were carried out in an ABI PRISM 7300 (Applied Biosystems, Tokyo, Japan). The specific sequences of primers for the analyzed genes are shown in Additional File 1 Table S1. The expression levels of genes were normalized to GAPDH. For miRNA real-time RT-PCR, total RNAs of approximately 100 ng were reverse-transcribed using the Taqman miRNA reverse transcription kit (Applied Biosystems). Real-time quantitative PCR amplification of the cDNA template was done using Taqman Universal PCR Master Mix (Applied Biosystems, Tokyo, Japan) in an ABI PRISM 7300 (Applied Biosystems). The PCR conditions were 50°C for 2 minutes and 95°C for 10 minutes followed by 50 cycles of 95°C for 15 seconds and 60°C for 1 minute. Taqman probes for human were used to assess the expression levels of miRNAs (hsa-miR-505, ID: 4373230, hsa-miR-130, ID: 000454, and hsa-miR-155, ID: 002623).

### 3'UTR assay plasmid constructs

A 1235 bp fragment from the 3'UTR of Akt3 containing the predicted target sequence of miR-505 (located at positions 529-535 of the Akt3 3'UTR) and a 934 bp fragment from the 3'UTR of Akt3 containing the predicted target sequence of miR-505 (located at positions 529-535 of this fragment) were PCR-cloned from MCF7-ADR cells isolated total RNA. Three prime A-overhang was added to the PCR products after 15 minutes of regular Taq polymerase treatment at 72°C. The PCR products were cloned into a pGEM-T easy vector (Promega). The amplified products were ligated into the NotI sites of the 3'UTR of the *Renilla *luciferase gene in the psi-check-2 plasmid (Promega) to generate psi-Akt3_1 (1235 bp) and psi-Akt3_2 (934 bp). Primer sequences used for PCR-cloning were shown as below: Akt3_F, GAGCCAGAGAGCATCTTTCC, Akt3_R1, GCTGCCTTAGTAAAATGCCC, and Akt3_R2, GACTTCACAGGCTGCTTTGG.

### Statistical analysis

The results are given as the mean ± s.d. Statistical analysis was conducted using the analysis of variance with the Student's t-test. A *P *value of 0.05 or less was considered to indicate a significant difference.

## Results

### An integrated genomic analysis unveils the status of cancer cells

**Figure 1:**
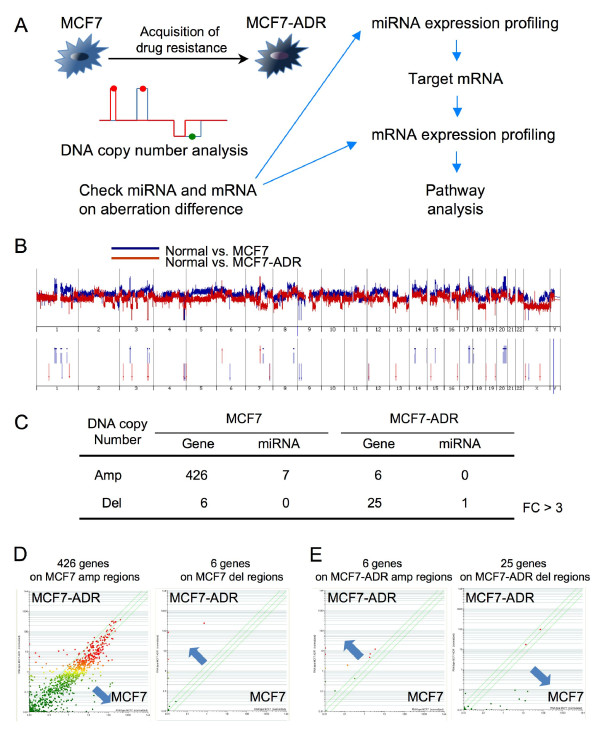
**An integrated genomic analysis to clarify drug resistance in MCF7-ADR (drug-resistant breast cancer cell line)**. (A) Schematic representation of an integrated genomic analysis. During the acquisition of drug resistance in breast cancer cell (MCF7; parental cell line and MCF7-ADR; drug resistance cell line), a large number of genomic alterations were raised, such as amplification or deletion, to modulate the expression of genes and miRNAs. Based on aCGH, miRNAs and genes on the aberration region of genome are selected for further analysis of its target genes and their associated-pathways *in silico*. (B) aCGH analysis of MCF7 and MCF7-ADR as compared with normal human female genome. Blue line shows normal vs. MCF7, and red line shows normal vs. MCF7-ADR (top). Amplified or deleted genome regions (fold change > 3) are highlighted (bottom). (C) The numbers of genes and miRNAs located on the amplified or deleted genome regions (FC > 3). (D and E) The expression tendency of genes located on the aberrant genome regions. 426 genes from MCF7 amplified regions and 6 genes from MCF7-ADR amplified regions are plotted in left panels of each scatter plot. Six genes from MCF7 deleted regions and 25 genes from MCF7-ADR deleted regions are plotted in right panels of each scatter plot.

MCF7-ADR is a multi-drug resistant cell line derived from MCF7 breast cancer cell line. We utilized these two cell lines to understand the regulatory network underlying drug resistance in breast cancer and conducted three types of genomic analysis, i.e. array-based comparative hybridization (aCGH) (Figure 1B), miRNA (Additional File 2 Figure. S1A) and gene expression (Additional File 2 Figure. S1B). Genes and miRNAs, which are located on the genome-amplified and -deleted regions, are expected to be responsible for drug resistance and sensitivity. Moreover, these three types of array data were used for miRNA target prediction and pathway to further elucidate key factors for drug resistance (Figure 1A).

As a consequence of array-based CGH, changes in DNA copy number in MCF7-ADR and MCF7 as compared with normal female genome were found in a large number of regions as amplification and deletion (Figure 1B). Accuracy of array-based CGH was validated by dye flip experiment that exhibited strikingly mirroring images (Additional File 3 Figure. S2). The numbers of genes and miRNAs located on the amplified and deleted genome regions (fold change > 3) in MCF7-ADR and MCF7 cells are shown (Figure 1C). On further comparison, a large number of changes in gene and miRNA expressions were observed between MCF7 and MCF7-ADR (Additional File 2 Figure. S1A and B). These genes with aberrant differences were observed predominantly in amplified region in MCF7 and, in contrast, they were mainly in deleted regions in MCF7-ADR (Figure 1B and 1C). In a different criterion of aCGH data (fold change > 2), we could see the same propensity more clearly (Additional File 4 Figure. S3A and B).

Because genomic amplification and deletion are thought to be associated with up- and down-regulation in expression, respectively, 426 genes on the amplified region in MCF7 and 6 genes on the deleted regions in MCF7 were plotted (Figure 1D). When the expression levels of amplified 426 genes and deleted 6 genes were checked, the expression of 426 genes tended to increase (Figure 1D left), and *vice versa *that of 6 genes tended to decrease in MCF7 as compared with MCF7-ADR (Figure 1D right), although these gene numbers were counted based on the comparison of MCF7 and normal female genome. Consistent with MCF7 result, we can see similar tendency in gene expression in the result of MCF7-ADR (Figure 1E), indicating that the gene expression levels and genomic alterations are broadly correlated in this experiment.

### An integrated genomic analysis reflects the unique features of drug resistance in breast cancer cells

**Figure 2:**
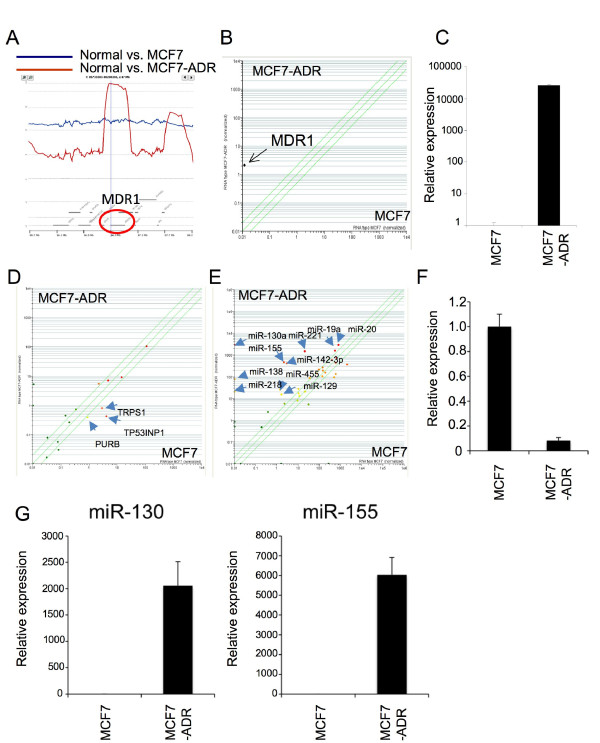
**Genomic amplification and overexpression of MDR1, and a number of miRNA regulation of TP53INP1**. (A) Twenty fold amplification of multi-drug resistance gene (MDR1) region in MCF7-ADR as compared with MCF7. (B and C) Overepression of MDR1 gene in MCF7-ADR by microarray and real-time PCR, respectively. (D) Scatter plot of miRNA target genes predicted by Targetscan. More than 15% of miRNAs up-regulated in MCF7-ADR (74 miRNAs) were predicted to potentially bind the 3'-UTR of these genes. Expression levels of three (TP53INP1, PURB and TRPS1) of them are clearly dwon-regulated. (E) Scatter plot of miRNAs that might bind to 3'UTR of TP53INP1 gene. (F and G) Confirmation of TP53INP1 gene, miR-130 and miR155 expression by real-time PCR. Standard deviation was calculated in triplicate determinants in the experiment.

We hypothesized that genes and miRNAs in the region of genomic alteration were relevant to drug resistance in MCF7-ADR cells. In the most amplified region in MCF7-ADR, we found MDR1 gene, which is an important efflux pump for drug resistance. Its genome locus was amplified more than 20-fold (Figure 2A), and its expression was up-regulated 800-fold or more in MCF7-ADR by microarray (Figure 2B). Expression level of MDR1 was confirmed by real-time RT-PCR and was observed to be remarkably up-regulated in MCF7-ADR consistent with our previous study (Figure 2C) [13], suggesting that the genomic amplification and overexpression of MDR1 were one of the reasons for drug resistance of MCF7-ADR.

**Figure 3:**
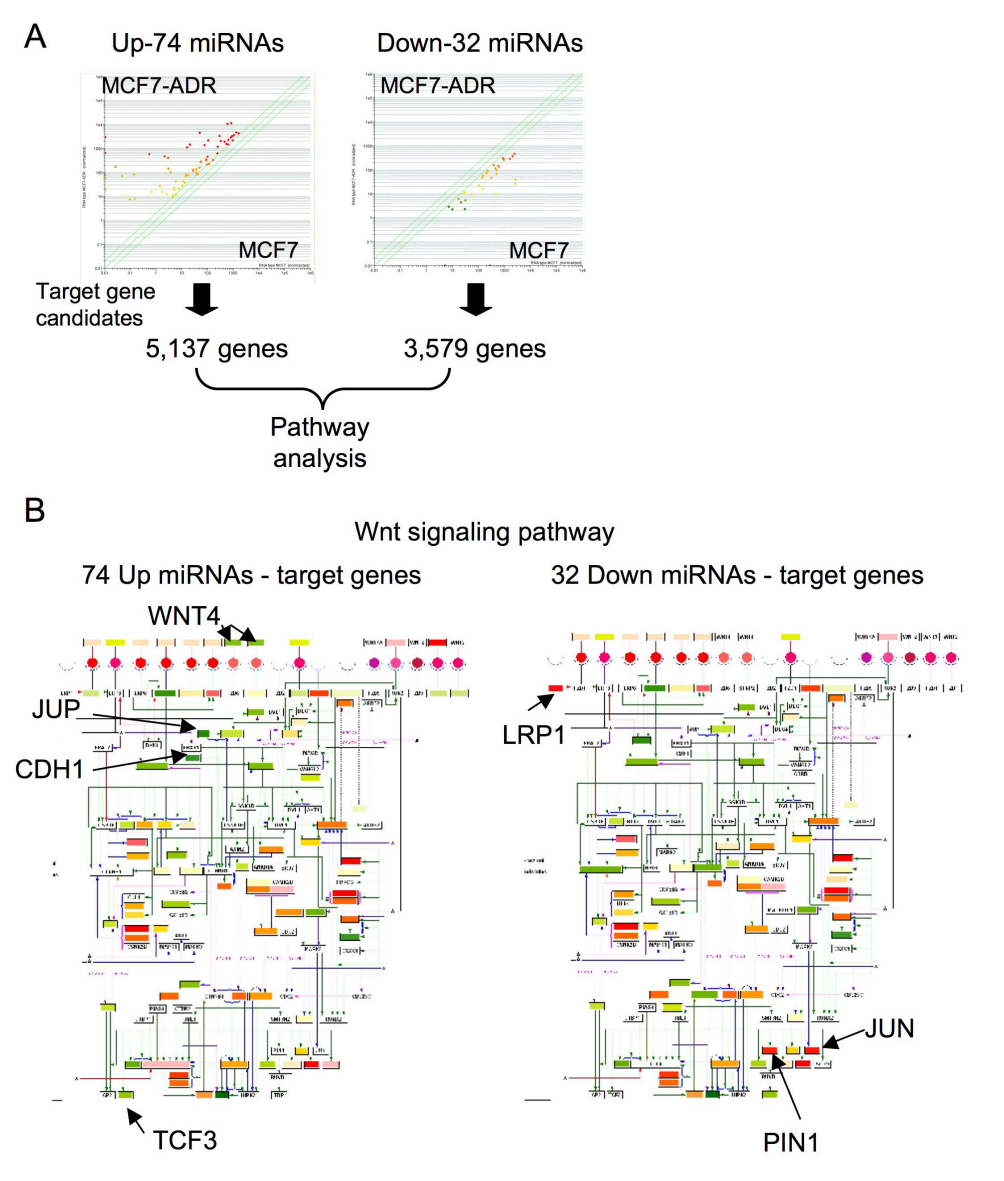
**Pathway enrichment analysis of up- and down-regulated miRNAs in breast cancer cells**. (A) Diagram of GO analysis of MCF7 and MCF7-ADR. In the comparison with MCF7 and MCF7-ADR, 5, 137 genes and 3, 579 genes are predicted as miRNA target candidates of 74 up-regulated miRNAs and 32 down-regulated miRNAs, respectively. With these predicted genes, enriched pathways are selected, as shown Table S1. (B) Wnt signaling pathway. 74 miRNA-targeted genes are plotted in Wnt signal pathway map (left), 32 miRNA-targeted genes are plotted (right). Red boxes show high expression genes, and green boxes show low expression genes.

Next, we tried to know the target genes of differentially-expressed miRNA in MCF7-ADR, because these genes might act as key molecules for drug resistance. Seventy-four miRNAs were 2 fold or more up-regulated in MCF7-ADR (Figure 3A), and we selected genes that have binding sites of more than 15% of up-regulated miRNAs in their 3'UTR. The scatter plot shows expression levels of these genes (Figure 2D), and 3 gene names are displayed because their expression levels are considerably down-regulated in MCF7-ADR. One of them is tumor protein p53 inducible nuclear protein 1 (TP53INP1) (Figure 2D), which has been recently shown to be suppressed by several miRNAs such as miR-130 and miR-155 [16,17]. Furthermore, decreased expression of TP53INP1 is involved in breast cancer progression [18]. As shown in Figure 2E, expression of miRNAs which potentially bind to 3'UTR of TP53INP1 were plotted, and it displays names of miRNAs whose expression levels were considerably up-regulated in MCF7-ADR. These included miR-130 and miR-155 also known as TP53INP1 binding miRNAs, and most of them are highly expressed in MCF7-ADR, indicating that these miRNAs and TP53INP1 expressions were inversely correlated. From the real-time RT-PCR analysis, down-regulation of TP53INP1 and up-regulation of miR-130 and miR-155 in MCF7-ADR as compared with MCF-7 (Figure 2F and 2G). Taken together with our results and recent publications, our integrated genomic analysis can clearly reflect the status of multi-drug resistant MCF7-ADR.

### Pathway analysis of up- and down-regulated miRNA target genes

Cancer cells abrogate the function of drug sensitive genes, such as tumor suppressor gene and related-gene pathway after the anticancer drug treatment, and thus we speculated that differentially-expressed miRNAs between MCF7-ADR and MCF7 controlled the gene pathway governing drug resistance. As shown in Figure 3A, 74 miRNAs and 32 miRNAs were up- and down-regulated in MCF7-ADR, respectively. Target gene prediction showed up-regulated 74 miRNAs had 5, 137 genes and down-regulated 32 miRNAs had 3, 579 genes as target candidates (Figure 3A). Expression levels of these genes were plotted in the scatter plots, however, decreasing and increasing expression tendencies were not clearly observed (data not shown). So, with these genes, we next checked what kind of gene pathways were expected to be regulated by differentially-expressed miRNAs. A large number of pathways were significantly chosen (Top 20 pathways shown in Additional File 5 Table S2). Surprisingly, many common pathways were enriched in both up-and down-regulated miRNA targeted genes, suggesting that the differentially-expressed miRNAs orchestrated to lead the global gene expression changes in MCF7-ADR. Intriguingly, these miRNAs seem to regulate some of drug resistance related signaling pathways, such as Wnt, insulin, EGFR1, MAPK and TGF-beta receptor (Additional File 5 Table S2, Figure. 3B and Additional File 6 Figure. S4). This data shows the possibility that differentially-expressed miRNAs might cooperatively regulate their target pathways and change drug resistance and sensitivity in MCF7-ADR.

### Identification of miRNAs that suppress cell proliferation in MCF7-ADR

**Figure 4:**
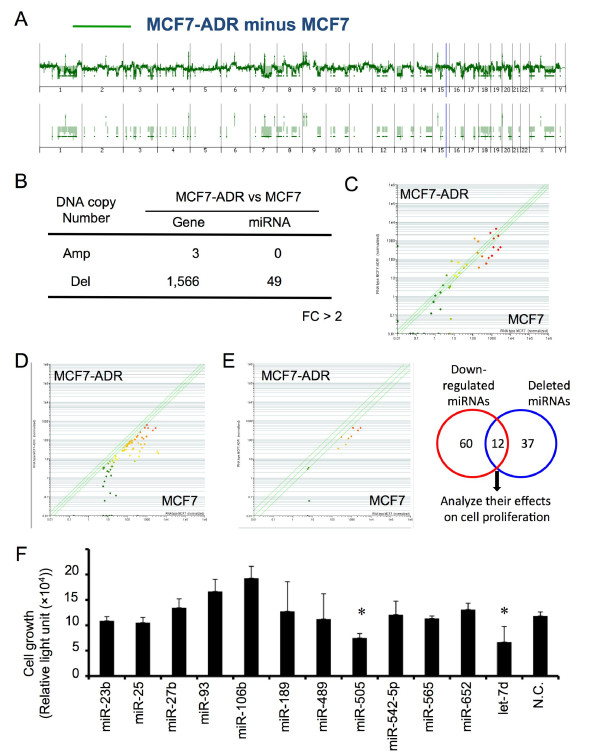
**Screening of miRNAs responsible for drug resistance in MCF7-ADR**. (A) Direct comparison of MCF7 and MCF7-ADR genomic status based aCGH. Modified Figure1B, differences in genomic status between MCF7 and MCF7-ADR are shown (FC > 2). (B) The number of genes and miRNAs located on the amplified or deleted genome regions in MCF7-ADR as compared with MCF7 (FC > 2). (C) Scatter plot of 49 miRNAs that locates in the deleted genomic regions in MCF7-ADR. (D) Scatter plot of 72 miRNAs whose expression was down-regulated in MCF7-ADR when compared with MCF7. (E) Twelve miRNAs (miR-23b, miR-25, miR-27b, miR-93, miR-106b, miR-189, miR-489, miR-505, miR-542-5p, miR-565, miR-652 and let-7d), which are overlapped between deleted and down-regulated in MCF7-ADR, are candidates responsible for drug resistance in MCF7-ADR. (F) Transfection analysis of selected 12 miRNAs in MCF7-ADR-Luc, which stably express luciferase. All miRNAs were transfected at 20 nM. Seventy-two hours after transfection, cell growth was estimated by luciferase activity in MCF7-ADR-Luc cells (*n *= 3-6 per group). P < 0.05.

We next explored whether the miRNAs that were located on the aberrant genome regions played a role in regulating drug resistance of MCF7-ADR. To this end, genomic status between MCF7-ADR and MCF7 were directly compared (Figure 4A). The numbers of genes and miRNAs that were located on the aberrant regions are shown (Figure 4B) and most of them were on the deleted regions in MCF7-ADR. A scatter plot showed 49 miRNAs on the deleted genomic regions in MCF7-ADR (Figure 4C) and expression levels of these 49 miRNAs had decreasing trend in MCF7-ADR. In this study, we focus on miRNAs whose expressions were down-regulated and genomic status was deleted in MCF7-ADR cells as compared to MCF7. Expression levels of 72 miRNAs were significantly down-regulated (p < 0.05, Additional File 7 Table S3 and Figure 4D) and 49 miRNAs (Additional File 8 Table S4 and Figure 4C) were located in deleted regions (FC > 2) in MCF7-ADR cells. Twelve miRNAs were overlapped between the 72 down-regulated miRNAs and 49 deleted miRNAs (Table 1 and Figure 4E).

**Table 1:** Selected 12 miRNAs

Name	MCF7-ADR/MCF7	Chromosome	Start	Stop
hsa-miR-565	0.48	3	45705468	45705564
hsa-miR-489	0.01	7	92951184	92951267
hsa-miR-25	0.16	7	99529119	99529202
hsa-miR-93	0.15	7	99529327	99529406
hsa-miR-106b	0.15	7	99529552	99529633
hsa-let-7d	0.37	9	95980937	95981023
hsa-miR-23b	0.18	9	96887311	96887407
hsa-miR-27b	0.12	9	96887548	96887644
hsa-miR-189 (miR-24)	0.00	9	96888124	96888191
hsa-miR-652	0.18	X	109185213	109185310
hsa-miR-542-5p	0.56	X	133501037	133505133
hsa-miR-505	0.53	X	138833973	138834056

To examine the functions of these miRNAs, we tested the effects of 12 miRNAs on cell proliferation in MCF7-ADR cells. The 12 miRNAs (miR-23b, miR-25, miR-27b, miR-93, miR-106b, miR-189, miR-489, miR-505, miR-542-5p, miR-565, miR-652, and let-7d) were transfected into MCF7-ADR-Luc cells (Figure 4F). Interestingly, miR-25, miR-93, and miR-106b are known as polycistronic miRNAs [19] and their expression and genomic region were coincidently changed between MCF7-ADR and MCF7 (Additional File 9 Figure. S5). Consistent with previous report, transfection with miR-93 and miR-106b promoted cell proliferation as compared with negative control miRNA, suggesting that they actually act as oncogenic miRNAs [19,20]. Inversely, transfection of miR-505 and let-7d inhibited the cell proliferation of MCF7-ADR-Luc cells. Let-7 family is a well known tumor suppressive miRNA as described in many reports [21-23]. Inhibitory effects of cell growth by miR-505 and let-7d transfection are at similar level (Figure 4F). These data suggest that miR-505 is a novel tumor suppressive miRNA and plays a role in the regulation of cell proliferation similar to let-7d.

### miR-505 inhibits cell growth by inducing apoptotic cell death in MCF7-ADR cells

**Figure 5:**
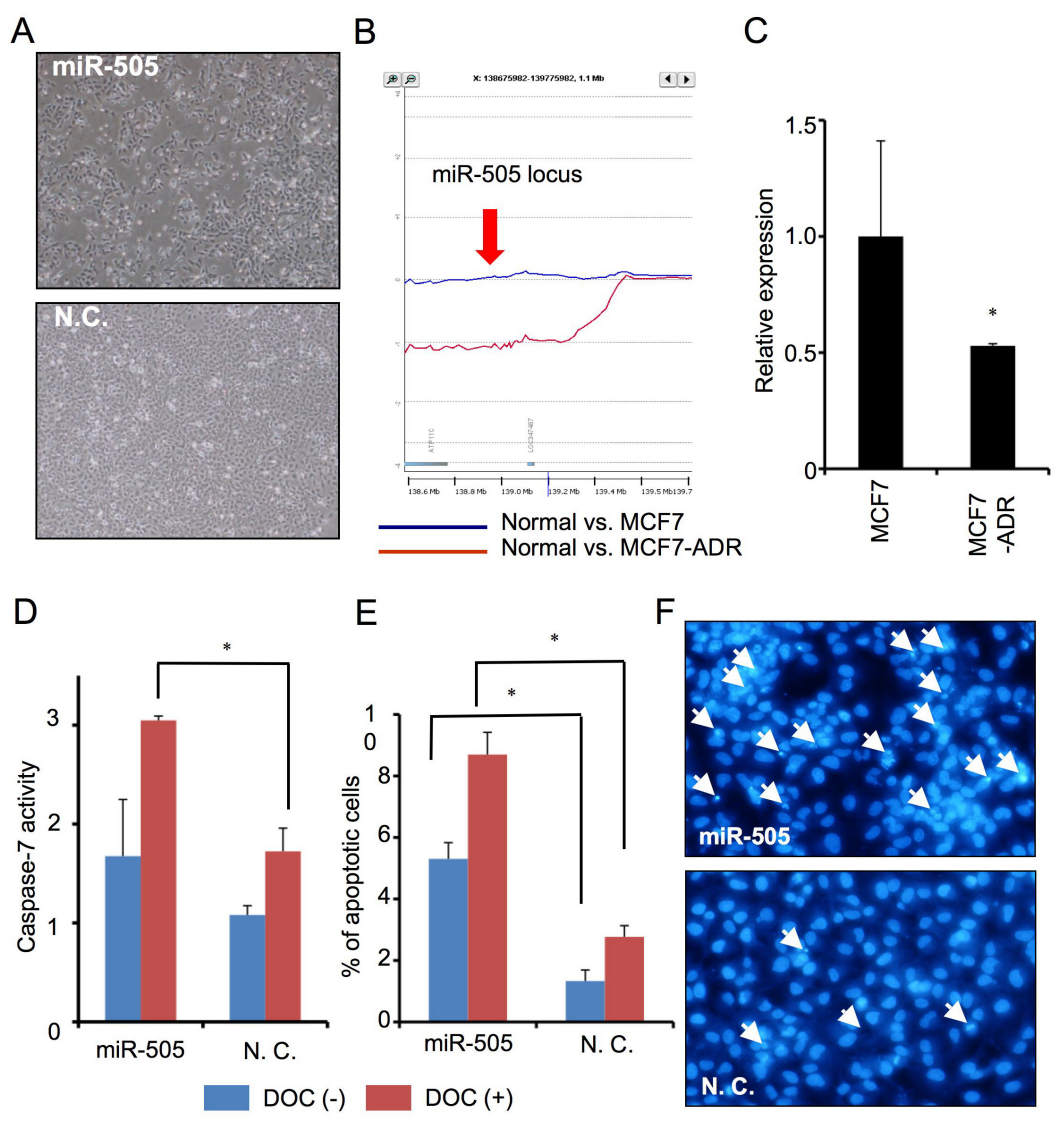
**miR-505 inhibits cell growth by inducing apoptotic cell death in MCF7-ADR**. (A) Images show typical MCF7-ADR cells transfected with miR-505 (top) and control miRNA (bottom) in 72 hours. (B) DNA copy number on the miR-505 locus of MCF7-ADR deleted as compared with that of MCF7. (C) Real-time RT-PCR analysis was performed to determine expression levels of miR-505 in parental cell line MCF7 and drug-resistant cell line MCF7-ADR. Error bar represents S.D. of triplicate determinations in a single experiment. P < 0.05. (D). Seventy-two hours after transfection, caspase-7 activity in the presence or absence of DOC (1 nM) was detected in miR-505 or control miRNA-transfected MCF7-ADR cells. n = 6, P < 0.001. (E) Condensed and fragmented apoptotic cells are counted in the presence or absence of DOC (1 nM) by staining with Hoechst dye. n = 4, P < 0.05. Mean ± S.D. is shown. (F) Images of apoptotic nuclear condensation and fragmentation. Seventy-two hours after transfection with miR-505 (top) or control miRNA (bottom), condensed and fragmented cells are observed. White arrows show apoptotic cells in culture.

Since transfection of miR-505 showed the inhibition of cell proliferation effectively and significantly (Figure 4F and 5A), we focused on miR-505 to evaluate its molecular function in this study. From the aCGH data, genomic region of miR-505 locus in MCF7-ADR was deleted (Figure 5B), in contrast it was intact in MCF7. The expression level of miR-505 was also decreased by real-time RT-PCR analysis in MCF7 and MCF7-ADR cells (Figure 5C). To further examine the mechanism of cell growth inhibition, we sought to check whether miR-505 is responsible for cell apoptosis in MCF7-ADR cells. A mature form of miR-505 was transfected into MCF7-ADR cells in the presence or absence of DOC (1 nM), as MCF7-ADR cells are resistant to DOC, and caspase-7 activity was measured to estimate apoptotic cell death in MCF7-ADR. The results of the caspase-7 assay indicated that transfection of miR-505 with DOC resulted in a marked induction of apoptosis (*p *< 0.05, Figure 5D), although no significant difference was seen in the samples without DOC. We validated this result by counting the Hoechst-stained cells showing apoptotic nuclear condensation and fragmentation and found that significantly higher apoptotic cell death was observed in cells with miR-505 than in control miRNA (*p *< 0.05, Figure 5E and 5F). Taken together, we concluded that transfection of miR-505 inhibit the growth of drug resistance cells, MCF7-ADR, through the inducing apoptosis.

### Akt3, correlates inversely with miR-505 expression, modulates drug sensitivity

**Figure 6:**
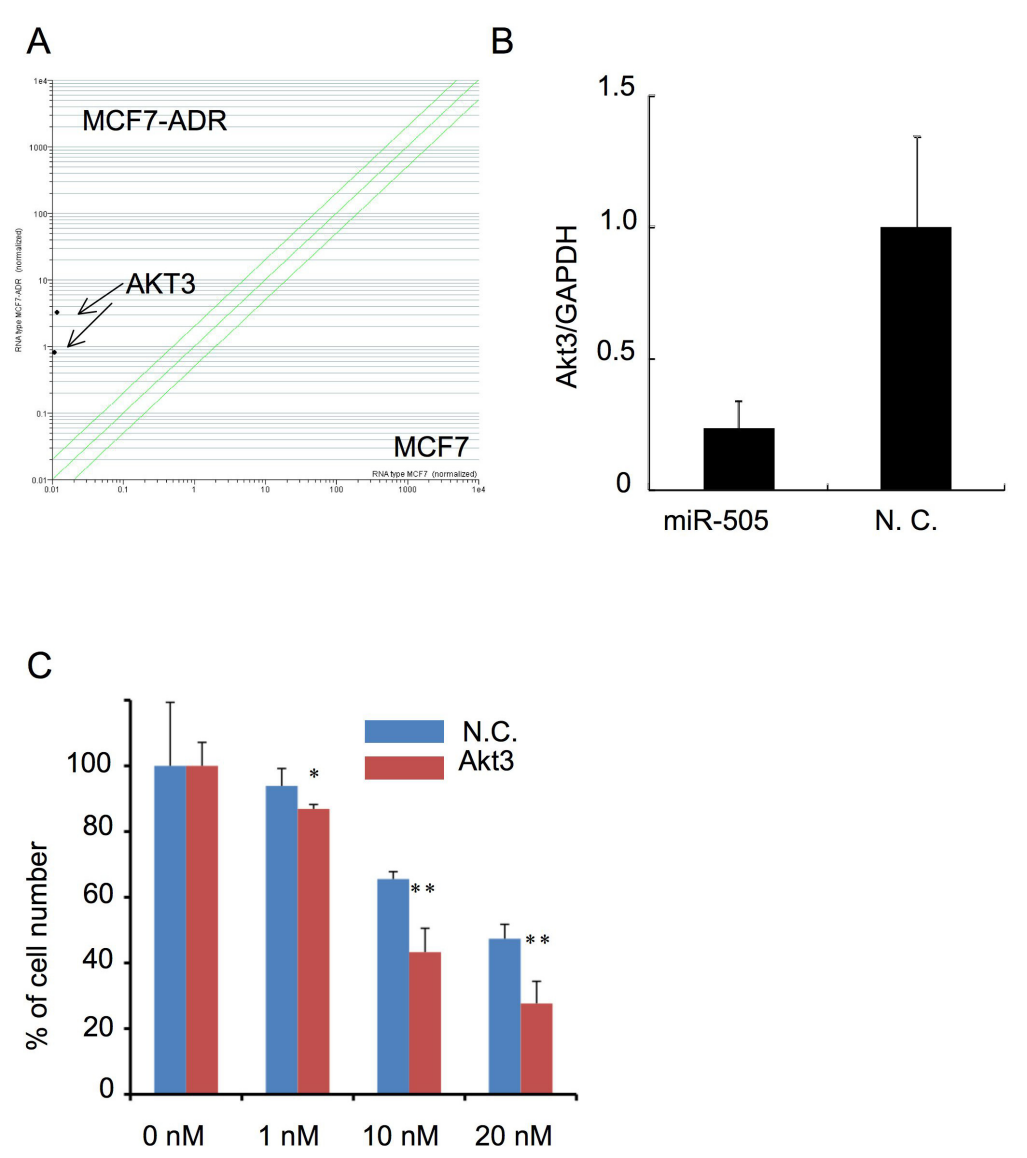
**Akt3, whose expression was inversely correlated with miR-505, is associated with drug resistance in MCF7-ADR cells**. (A) Scatter plot of 2 probes of Akt3 genes that up-regulated in MCF7-ADR cells. (B) Real-time RT-PCR analysis for Akt3 in miR-505 or control miRNA-transfected cells. Seventy-two hours after transfection with miR-505 or control miRNA, Akt3 expression levels were determined. Expression levels were normalized to GAPDH expression levels. N = 9, mean ± S.D. is shown. (C) Down-regulation of Akt3 induced cell growth arrest in MCF7-ADR cells at 1, 10 and 20 nM DOC conditions. Cell growth was calculated by luciferase activity. n = 3, ∗, P < 0.05. ∗∗, 0.01.

It has been already known that drug resistance in cancer cells was an acquired characteristic by activation of multiple drug resistance-responsible genes. To investigate what kind of the genes are responsible for the drug sensivity by miR-505 induce gene suppression, we combined gene expression data and gene ontology (Figure 6A). As for gene expression data, since the expression level of miRNA-regulating genes was up-regulated when miRNA expression was lower in MCF7-ADR than in MCF7 cells, up-regulated genes judged by the T-test (1, 758 genes, Additional File 10 Table S5) were selected as candidates of miR-505. As miR-505-targeted genes, apoptosis-related genes (153 genes, Additional File 11 Table S6) were chosen using a database (KEGG web site, http://www.genome.jp/kegg/) because transfection of miR-505 induced apoptotic cell death in MCF7-ADR-Luc cells. By combining these data, we postulated that Akt3 gene is candidate of miR-505-regulating gene, which is responsible for the drug resistance in breast cancer cell (Figure 6A). Notably, several studies have reported that Akt3 promotes melanoma development [24] and that the down-regulation of Akt3 distinctly inhibited the proliferation of ovarian cancer cell lines by slowing G2-M phase transition [25]. Given this evidence and the observed phenotype in miR-505-transfected MCF7-ADR-Luc cells, we sought to determine whether Akt3 was a target of miR-505 or not. As shown in Figure 6B, decrease in relative gene expression was observed with miR-505 in MCF7-ADR-Luc cells, suggesting that miR-505 suppresses the gene expression of Akt3. However, we found that miR-505 could not bind to the 3'-UTR of Akt3 gene (Additional File 12 Figure. S6), indicating that down-regulation of Akt3 after miR-505 overexpression was caused by indirect effect of miR-505-mediated gene suppression. Finally, transfection of Akt3 siRNA was conducted to examine whether down-regulation of the Akt3 gene induced cell growth arrest in the presence or absence of DOC. Three days after transfection, the expression of Akt3 was clearly suppressed (Additional File 13 Figure. S7), and cell growth rates were assayed with or without 1, 10, and 20 nM DOC conditions. A slight decrease in cell growth was observed with 1 nM DOC condition, and more remarkable decreases was detected with 10 and 20 nM DOC conditions in Akt3 siRNA-transfected MCF7-ADR cells than in control siRNA-transfected cells (Figure 6C). Therefore, our data show that Akt3, whose expression is correlated conversely with miR-505, regulates DOC sensitivity in MCF7-ADR cells.

## Discussion

According to recent high-throughput analyses of both coding and non-coding genes, cancer progression is caused by genetic alteration involving structural and expression abnormalities of oncogenes and tumor suppressor genes [6,26]. In this study, we performed an integrated genomic analysis to link miRNA expression data to aCGH and gene expression microarray, using the parental cell line MCF7 and the drug-resistant cell line MCF7-ADR, to examine the molecular mechanism governing drug resistance in breast cancer. We found that the expression of miR-505 was down-regulated, and its genomic region was deleted in MCF7-ADR cells, which provided evidence that miR-505 was a tumor suppressive miRNA and had a pivotal role for inducing apoptosis in drug resistant cancer cells. In addition, by using the data of gene expression and bioinformatics analysis (gene function), our data identified Akt3 whose expression was conversely correlated with miR-505, which modulated drug sensitivity in MCF7-ADR.

Akt is a homolog of the retroviral oncogene v-Akt, which is ubiquitously expressed and has 3 members; Akt1, Akt2, and Akt3 [27]. Downstream genes of the Akt signal pathway modulate the cell cycle, DNA repair, and nitric oxide production. Moreover, Akt inhibits apoptotic cell death by inactivation of a key apoptotic molecule and is broadly activated in various kinds of cancer. Importantly, the Akt signal pathway is tightly related to drug resistance in cancer. Several studies have reported that inactivation of Akt promotes drug-induced apoptosis [28,29]. Therefore, inhibition of Akt3 is a therapeutic strategy for cancers by inducing apoptotic cell death and reversing drug resistance. However, our results showed that inhibition of Akt3 was less effective than transfection of miR-505 in cell growth arrest. A simple explanation of a low effect might be that miRNA could modulate the expression of a large number of downstream target genes in a highly orchestrated manner to control apoptosis and cell cycle processes

Concerning the variations in the DNA copy numbers and genomic aberrations, several reports have shown the deletions of miRNAs that act as tumor suppressors, namely miR-15, miR-16, and miR-34a. They are observed in cancer, and down-regulation of these miRNAs contributed to cancer progression, indicating that variations in DNA copy numbers are closely associated with miRNA expression and carcinogenesis [9,10]. Meanwhile, several data provided evidences that miRNA expressions were regulated by epigenetic modifications, such as DNA hypomethylation and hypermethylation. It has been demonstrated that miR-342 was methylated in colorectal cancer and the reconstitution of miR-342 induced apoptosis in a colorectal cancer cell line [30]. A recent study showed that miR-127, which was embedded in a CpG island, was expressed in normal fibroblast but silenced or down-regulated in cancer cells. The silencing of the miRNA promoter region of miR-127 was mediated by CpG island hypermethylation, which could be reversed by simultaneous treatment with the chromatin-modifying drugs 5-aza-2'-deoxycytidine and 4-phenylbutyric acid [31]. In addition, recent studies have shown that impaired miRNA processing contributes to a decrease in mature miRNA expression and accelerates tumorigenesis [32], and a number of groups have also revealed that miRNA expression is regulated by transcription factors and cytokines as well as coding genes [33]. In this study, we found 12 miRNAs whose expressions are down-regulated and their genomic regions are deleted in MCF7-ADR. Interestingly, some of them, such as miR25-93-106b and miR-23b-27b-189, are located in close proximity and their expressions are expected to be regulated by the same transcriptional regulators. Curiously, miR-23b-27b-189 is localized on the Ch9q22.3, and LOH of this region is strongly correlated with cancer progression and lymph node metastasis [34-36]. In our assay we could not observe any significant differences, however, they could be related to malignancy in different aspects [37,38].

Pathway analysis, which was based on miRNA target prediction, proved that differentially-expressed miRNA cooperatively regulated a large number of signaling pathways, including Wnt, insulin, EGFR1, MAPK and TGF-β receptor, which are relevant to drug resistance as well as tumorigenesis. Concerning the Wnt signaling pathway, it has been reported that activation of the Wnt/β-catenin pathway plays critical roles in establishment of MLL leukemic stem cells and conferring drug-resistant properties [39]. Additionally, activation of Wnt/beta-catenin signaling in plasma cells induced chemoresistance [40], and RNAi-mediated gene silencing of β-catenin negatively regulated drug-induced apoptosis [41]. Therefore, Wnt/β-catenin signaling pathway would be a potential therapeutic target to sensitize drug-resistant cancer cell. Furthermore, other pathways were also reported to be associated with drug resistance and apoptosis. Sequential treatment of TGF-β induced MDR1 expression in rat hepatocytes [42]. In contrast, TGF-β also induces apoptotic cell death in hepatocytes and activation of the MAPK/ERK pathway confers resistance to TGF-β-induced cell death [43]. Our findings showed that a lot of pathways were commonly enriched in up- and down-regulated miRNA targets, however, it is hard to decide whether these pathways are positively or negatively regulated in MCF7-ADR, because a large number of target candidates exist in each signaling pathway. Further investigation such as systems biology would be needed to clarify this point.

We believe that the integrative genomic analyses as described here have a huge potential to fundamentally understand transcriptional regulatory networks and identify the novel molecular targets for therapy in the field of cancer biology. By integrating array data and bioinformatics, it could be possible to expeditiously explore the key molecule in the all aspects of pathophysiology. Our studies by means of an integrated genomic analysis not only identified miR-505 as a tumor suppressive miRNA that inhibited cell proliferation by inducing apoptotic cell death but also, more broadly highlighted that various genes and miRNAs orchestrate to temper the drug resistance by intricately controlling genomic status, gene and miRNA expression in cancer cells. Thus, it would be a useful approach to accelerate the understanding of cancer genetics and discover the key targets for diagnosis, prognosis and therapy.

## Competing interests

The authors declare that they have no conflict of interest. KM, TT, RH, and YF are Agilent employees.

## Authors' contributions

YYa, YYo, KM, RT, and FT carried out the experimental work, YYa, KM, TT, RH and YF provided data analysis, YYa, TK, NK and TO designed the study and YYa, NK and TO participated in writing the paper. All authors read and approved the manuscript.

## Supplementary Material

Additional file 1 **Table S1**. Primer list for real-time PCR. Click here for file

Additional file 2 **Figure. S1**. Microarray analysis of gene expression and miRNA in MCF7-ADR and MCF7. (A) Scatter plot of gene expression. 41, 000 probes (n = 3). (B) Scatter plot of miRNA expression. 470 probes (n = 2). Click here for file

Additional file 3 **Figure. S2**. Validation of accuracy of aCGH in MCF7 and MCF7-ADR. (A) aCGH analysis of MCF7. (B) aCGH analysis of MCF7-ADR. For each sample, the experiment was repeated once, wherein the dye was reversed between the experimental and the reference sample, in order to account for dye-incorporation bias. An aberration filter was set at 2 for the minimum number of probe region and 1 for minimum absolute average log2 ratio for regions in the CGH Analytics to reduce false positives. Click here for file

Additional file 4 **Figure. S3**. aCGH analysis of MCF7 and MCF7-ADR as compared with normal human female genome. (A) Blue line shows normal vs. MCF7, and red line shows normal vs. MCF7-ADR (top). Amplified or deleted genome regions (fold change > 2) are highlighted (bottom). (B) The numbers of genes and miRNAs located on the amplified or deleted genome regions (FC > 2). Click here for file

Additional file 5 **Table S2**. Up and Down miRNA target gene related pathways. Click here for file

Additional file 6 **Figure. S4**. TGF-β signaling pathway. Seventy-four miRNA-targeted genes are plotted in TGF-β signaling pathway map (left), 32 miRNA-targeted genes are plotted (right). Click here for file

Additional file 7 **Table S3**. List of down-regulated miRNAs in MCF7-ADR. Click here for file

Additional file 8 **Table S4**. List of miRNAs located in genome deletion regions in MCF7-ADR. Click here for file

Additional file 9 **Figure. S5**. Polycistronic miRNAs; miR-106-25 cluster. miR-106-25 cluster is located on the deleted genomic region, and the expression is coincidently downregulated. Click here for file

Additional file 10 **Table S5**. List of up-regulated genes in MCF7-ADR judged by T-test in comparison with MCF7. Click here for file

Additional file 11 **Table S6**. List of genes accosiated with apoptosis (KEGG web site). Click here for file

Additional file 12 **Figure. S6**. The 3'-UTR assay of Akt3 by miR-505 in MCF7-ADR cells and HEK293 cells. (A) MCF7-ADR cells and (B) HEK293 cells were co-transfected with pre-miR-505 or pre-NC and the psi-Akt3_1 or with psi-Akt3_2. After 48 h, luciferase activities were measured. n.s. represents not significant. Click here for file

Additional file 13 **Figure. S7**. Real-time RT-PCR analysis of Akt3 gene by transfection of siRNA in MCF7-ADR cells. Real-time RT-PCR analysis was performed to examine Akt3 from RNA extracted from MCF7-ADR cells transfected with either Akt3 siRNA or negative control siRNA. Akt3 expression levels were normalized to GAPDH expression levels. The mean ± S. D. of results from triplicate transfections is shown. Results represent the mean ± S. D. (n = 3). Since Akt3 siRNA-1 was most effectively inhibited the expression of Akt3 genes, it was used for the analysis of cell growth arrest. Click here for file
