# Supplementary material for: An integrative genomic analysis revealed the relevance of microRNA and gene expression for drug-resistance in human breast cancer cells
Source: Mol Cancer. 2011 Nov 3;10:135. doi: 10.1186/1476-4598-10-135 (PMC3247093; doi:10.1186/1476-4598-10-135)
Supplement: Additional file 1 — Table S1. Primer list for real-time PCR. [file 1476-4598-10-135-S1.PDF]

Table S1 Primer list for real-time PCR

Gene	Forword primer	Reverse primer	annealing (℃)
MDR	5'-ATTCGCTATGGCCGTGAAA-3'	5'-TTCTGCCCACCACTCAACTG-3'	55
TP53INP1	5'-GACTTCATAGATACTTGCAC	5'-ATTGGACATGACTCAAACTG	60
Akt3	5'-AGGACCGCACACGTTTC-3’	5'-CAGTCTACTGCTCGGCCATA-3’	55
GAPDH	5'-GAAGGTGAAGGTCGGAGT-3’	5'-GAAGATGGTGATGGGATTTC-3’	55
