# Supplementary figures and images for: An integrative genomic analysis revealed the relevance of microRNA and gene expression for drug-resistance in human breast cancer cells
Source: Mol Cancer. 2011 Nov 3;10:135. doi: 10.1186/1476-4598-10-135 (PMC3247093; doi:10.1186/1476-4598-10-135)

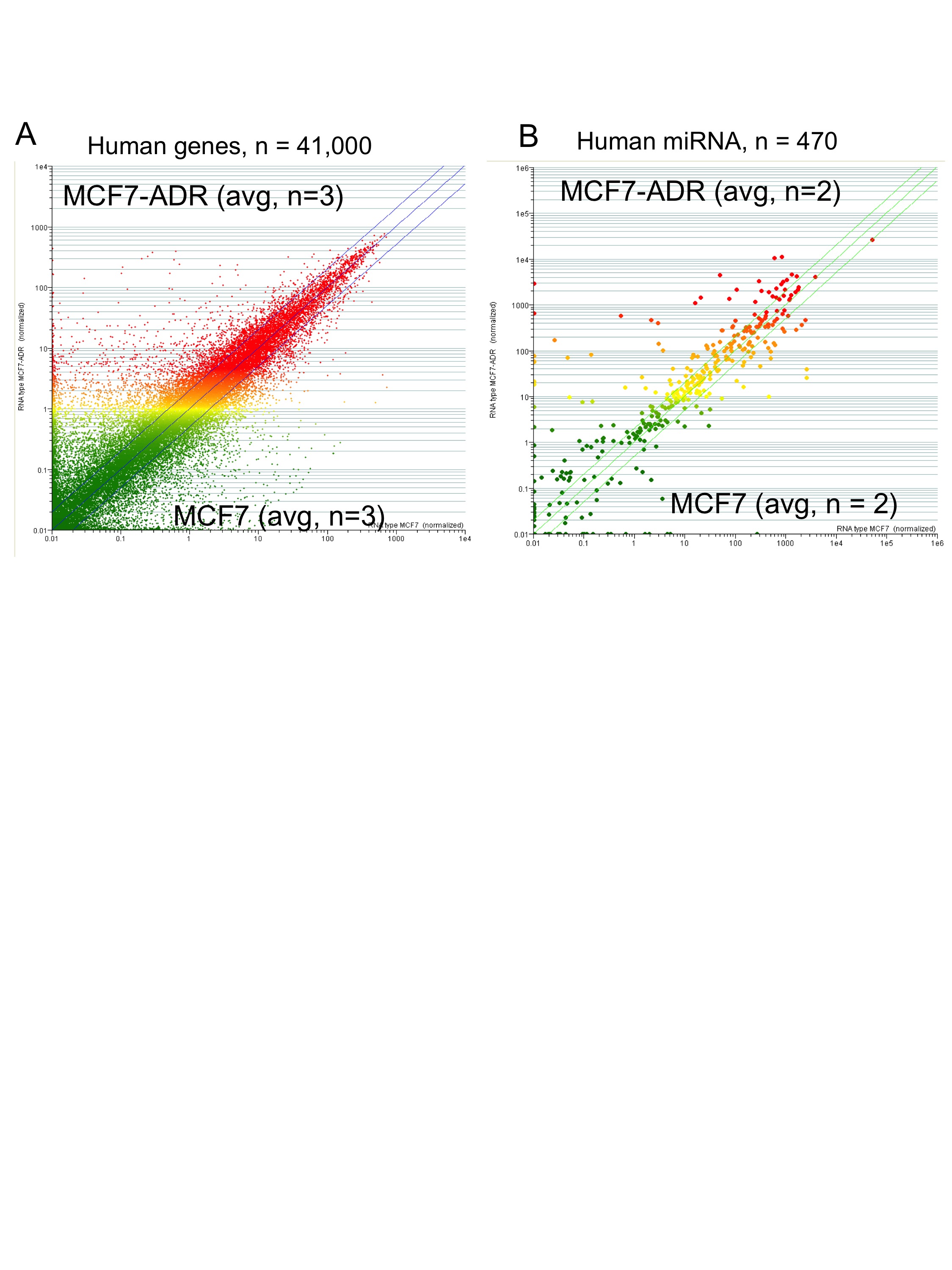

Supplement: Additional file 2 — Figure. S1. Microarray analysis of gene expression and miRNA in MCF7-ADR and MCF7. (A) Scatter plot of gene expression. 41, 000 probes (n = 3). (B) Scatter plot of miRNA expression. 470 probes (n = 2). [file 1476-4598-10-135-S2.JPEG]

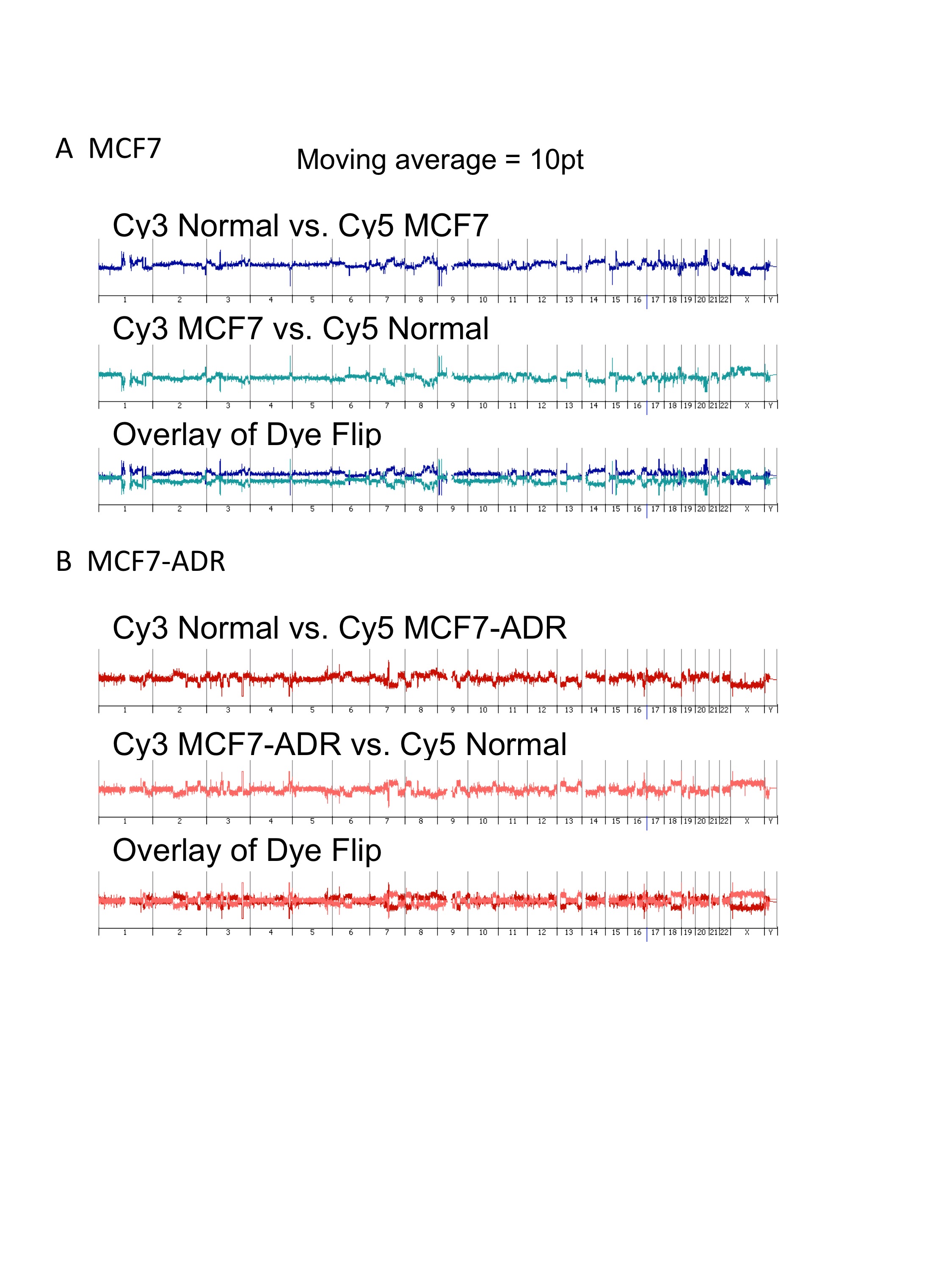

Supplement: Additional file 3 — Figure. S2. Validation of accuracy of aCGH in MCF7 and MCF7-ADR. (A) aCGH analysis of MCF7. (B) aCGH analysis of MCF7-ADR. For each sample, the experiment was repeated once, wherein the dye was reversed between the experimental and the reference sample, in order to account for dye-incorporation bias. An aberration filter was set at 2 for the minimum number of probe region and 1 for minimum absolute average log2 ratio for regions in the CGH Analytics to reduce false positives. [file 1476-4598-10-135-S3.JPEG]

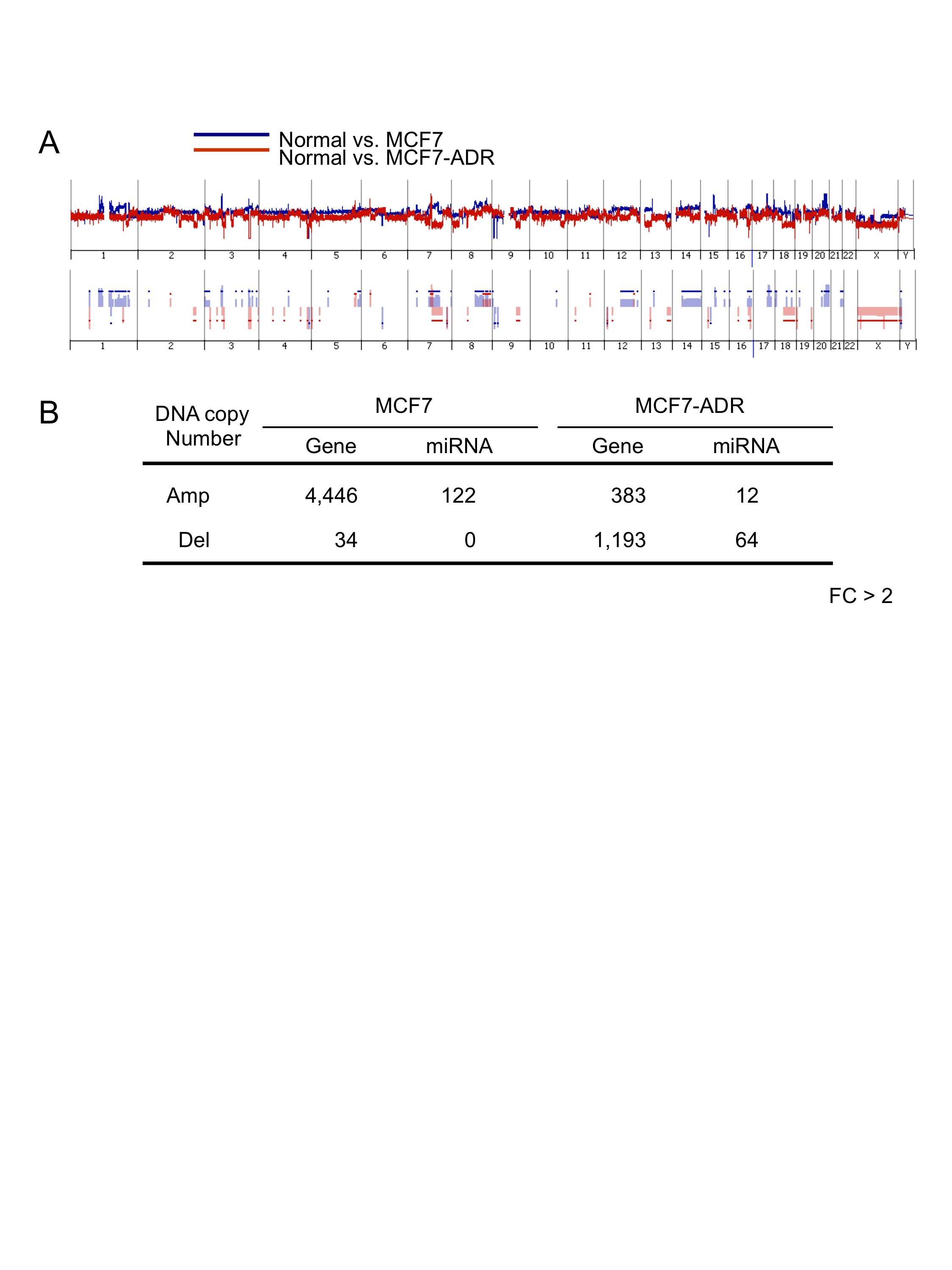

Supplement: Additional file 4 — Figure. S3. aCGH analysis of MCF7 and MCF7-ADR as compared with normal human female genome. (A) Blue line shows normal vs. MCF7, and red line shows normal vs. MCF7-ADR (top). Amplified or deleted genome regions (fold change > 2) are highlighted (bottom). (B) The numbers of genes and miRNAs located on the amplified or deleted genome regions (FC > 2). [file 1476-4598-10-135-S4.JPEG]

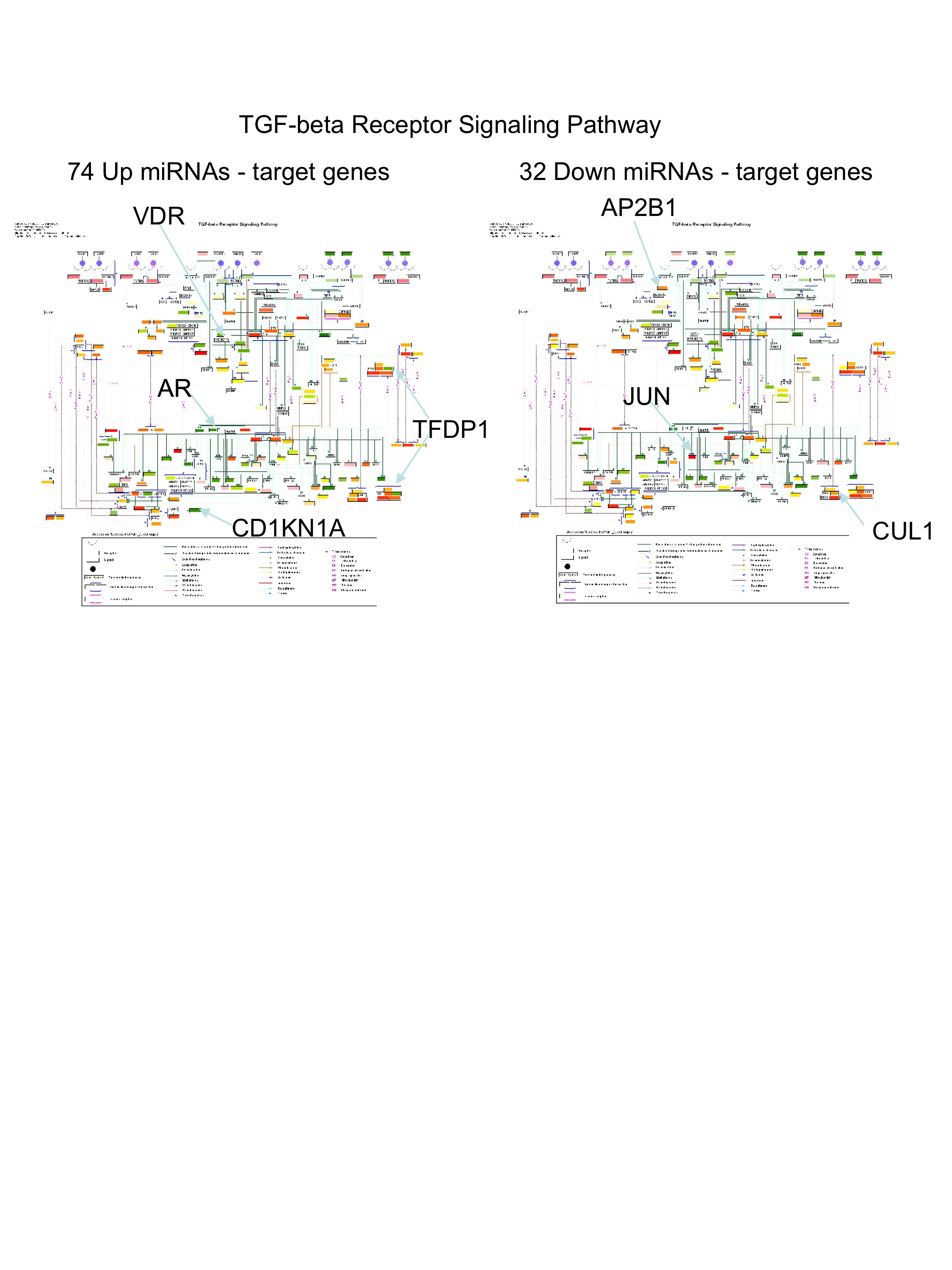

Supplement: Additional file 6 — Figure. S4. TGF-β signaling pathway. Seventy-four miRNA-targeted genes are plotted in TGF-β signaling pathway map (left), 32 miRNA-targeted genes are plotted (right). [file 1476-4598-10-135-S6.JPEG]

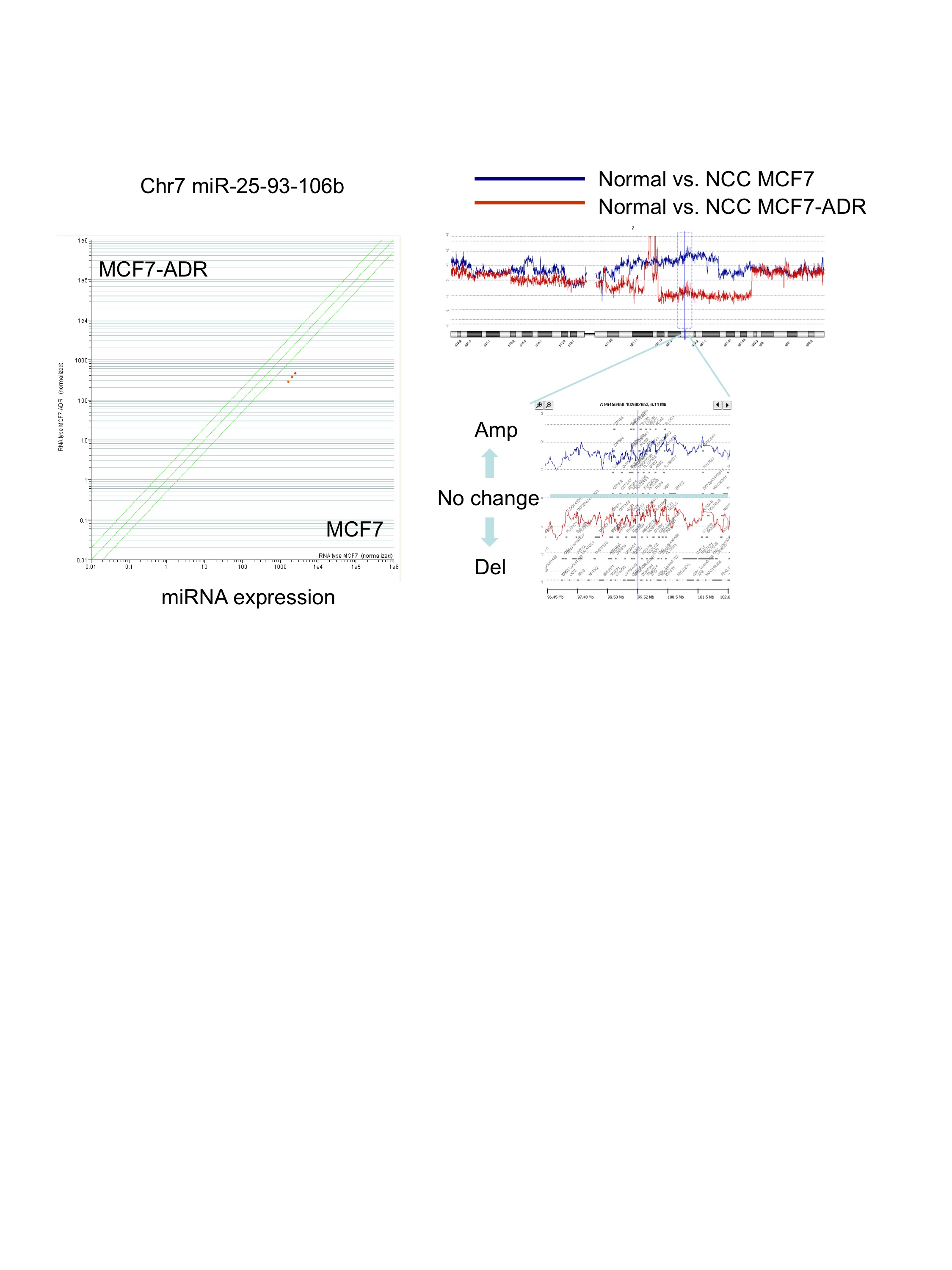

Supplement: Additional file 9 — Figure. S5. Polycistronic miRNAs; miR-106-25 cluster. miR-106-25 cluster is located on the deleted genomic region, and the expression is coincidently downregulated. [file 1476-4598-10-135-S9.JPEG]

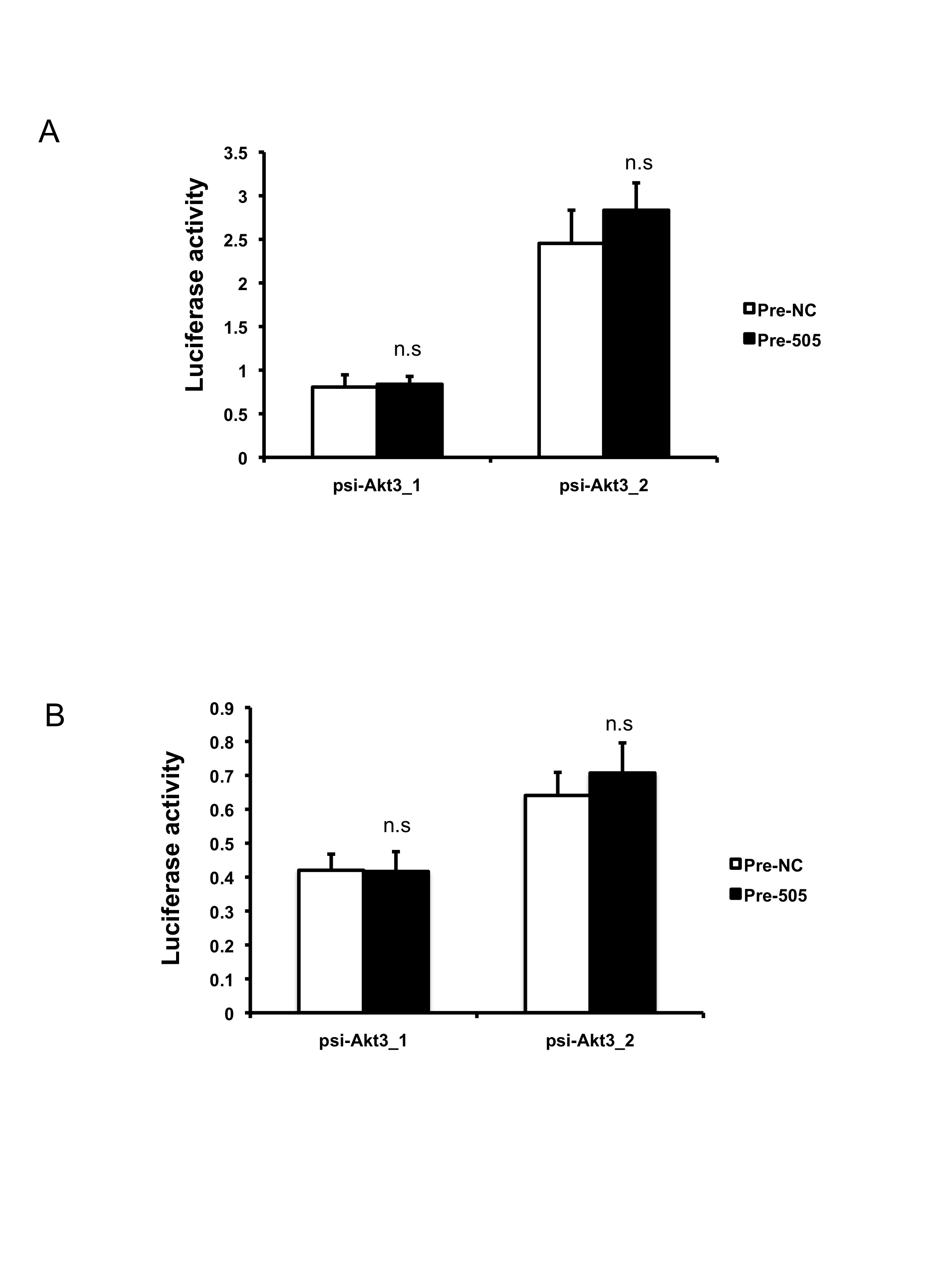

Supplement: Additional file 12 — Figure. S6. The 3'-UTR assay of Akt3 by miR-505 in MCF7-ADR cells and HEK293 cells. (A) MCF7-ADR cells and (B) HEK293 cells were co-transfected with pre-miR-505 or pre-NC and the psi-Akt3_1 or with psi-Akt3_2. After 48 h, luciferase activities were measured. n.s. represents not significant. [file 1476-4598-10-135-S12.JPEG]

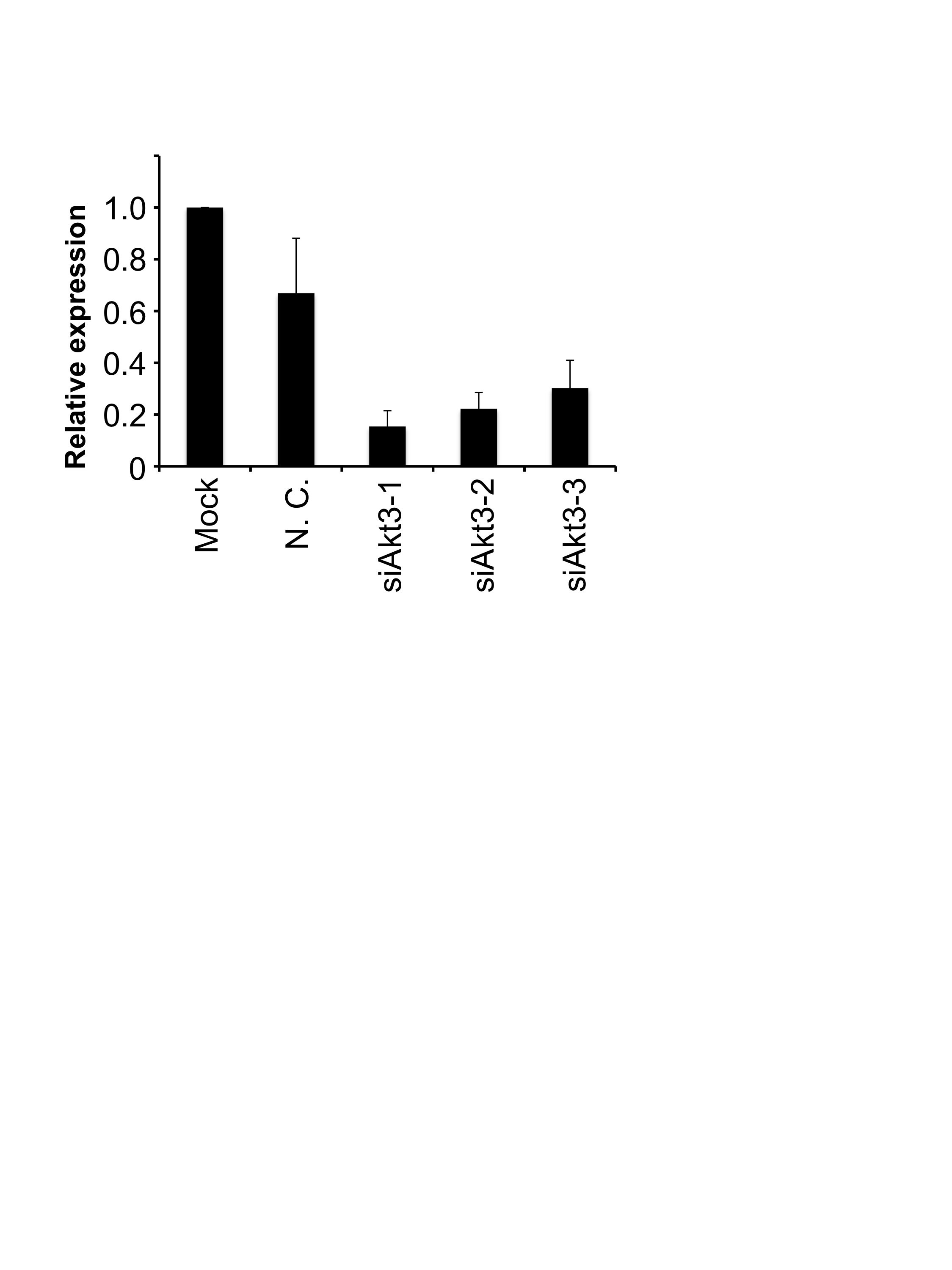

Supplement: Additional file 13 — Figure. S7. Real-time RT-PCR analysis of Akt3 gene by transfection of siRNA in MCF7-ADR cells. Real-time RT-PCR analysis was performed to examine Akt3 from RNA extracted from MCF7-ADR cells transfected with either Akt3 siRNA or negative control siRNA. Akt3 expression levels were normalized to GAPDH expression levels. The mean ± S. D. of results from triplicate transfections is shown. Results represent the mean ± S. D. (n = 3). Since Akt3 siRNA-1 was most effectively inhibited the expression of Akt3 genes, it was used for the analysis of cell growth arrest. [file 1476-4598-10-135-S13.JPEG]
