# Supplementary material for: An integrative genomic analysis revealed the relevance of microRNA and gene expression for drug-resistance in human breast cancer cells
Source: Mol Cancer. 2011 Nov 3;10:135. doi: 10.1186/1476-4598-10-135 (PMC3247093; doi:10.1186/1476-4598-10-135)
Supplement: Additional file 5 — Table S2. Up and Down miRNA target gene related pathways. [file 1476-4598-10-135-S5.PDF]

Table S2 Up and Down miRNA target gene related pathways

Enriched pathway in Up miRNA taget genes (n = 5,137)		Enriched pathway in Down miRNA taget genes (n = 3,579)	
Pathway name	p value	Pathway name	p value
MAPK signaling pathway-Homo Sapiens	3.03E-17	EGFR1 Signaling Pathway	4.08E-19
Human Insulin Signaling	4.49E-17	Human Insulin Signaling	8.54E-17
EGFR1 Signaling Pathway	2.55E-16	TGF-beta Receptor Signaling Pathway	4.46E-16
Axon guidance-Homo Sapiens	3.21E-16	Axon guidance-Homo Sapiens	2.15E-15
MAPK signaling pathway	1.77E-12	Wnt signaling pathway	1.04E-13
Focal adhension-Homo Sapiens	7.78E-12	MAPK signaling pathway-Homo Sapiens	5.11E-13
TGF-beta Receptor Signaling Pathway	1.31E-11	Wnt signaling pathway-Homo Sapiens	9.72E-11
Myometrial Relaxation and Contraction Pathway	2.81E-11	MAPK signaling pathway	2.21E-10
Wnt signaling pathway-Homo Sapiens	4.86E-11	Gap junction-Homo Sapiens	6.66E-10
Wnt signaling pathway	5.77E-11	B Cell Receptor Signaling Pathway	1.66E-09
Regulation of actin cytoskelton-Homo Sapiens	7.75E-11	Myometrial Relaxation and Contraction Pathway	1.97E-09
IL-6 signaling pathway	9.42E-11	Adipogenesis Human	7.54E-07
Focal adhension	5.71E-10	Delta-Notch Signaling Pathway	9.92E-08
Calcium Regulation in the Cardiac Cell	1.91E-09	Cell cycle-Homo Sapiens	1.25E-07
Adipogenesis Human	3.14E-09	Calcium Regulation in the Cardiac Cell	2.76E-07
Integrin-mediated cell adhension	4.07E-09	Regulation of actin cytoskelton-Homo Sapiens	3.01E-07
Regulation of actin cytoskelton	1.13E-08	Long-term potentiation-Homo Sapiens	6.31E-07
Androgen Receptor Signaling Pathway	2.33E-08	Focal adhension-Homo Sapiens	9.87E-07
B Cell Receptor Signaling Pathway	4.35E-08	GnRH signaling pathway-Homo Sapiens	1.03E-06
Calcium signaling pathway-Homo Sapiens	1.16E-07	IL-6 signaling pathway	1.08E-06

Top20 signaling pathways are shown based on p value.
