# Supplementary material for: An integrative genomic analysis revealed the relevance of microRNA and gene expression for drug-resistance in human breast cancer cells
Source: Mol Cancer. 2011 Nov 3;10:135. doi: 10.1186/1476-4598-10-135 (PMC3247093; doi:10.1186/1476-4598-10-135)
Supplement: Additional file 7 — Table S3. List of down-regulated miRNAs in MCF7-ADR. [file 1476-4598-10-135-S7.PDF]

Table S3 List of down-regulated miRNAs in MCF7-ADR

	MCF7		MCF7-ADR	
**Gene name**	**Normalized**	**StdErr Norm**	**Normalized**	**StdErr Norm**
hsa-miR-141	4013.94	226.06	19.37	1.94
hsa-miR-375	335.42	20.83	0.01	1.57
hsa-miR-136	12.70	1.58	0.15	173.05
hsa-miR-132	12.32	1.57	7.16	1.62
hsa-miR-34a	390.77	21.36	13.58	1.86
hsa-miR-342	358.68	18.75	68.76	3.92
hsa-miR-377	14.36	1.68	0.01	1.57
hsa-miR-376a	15.48	1.38	0.01	1.29
hsa-miR-345	134.90	8.21	15.32	1.85
hsa-miR-200c	3479.96	182.88	24.43	2.06
hsa-miR-127	15.18	1.63	4.03	1.59
hsa-miR-200a*	13.98	1.31	1.30	1.29
hsa-miR-200b	555.01	29.46	38.58	2.62
hsa-let-7d	1160.77	59.89	425.85	23.08
hsa-miR-148a	83.03	4.54	30.86	2.28
hsa-let-7e	1012.31	51.47	621.16	32.14
hsa-miR-106b	2791.38	148.07	426.84	23.30
hsa-miR-25	965.39	54.81	155.15	8.98
hsa-miR-326	5.53	1.23	0.36	1.33
hsa-miR-27b	487.05	29.18	58.20	3.90
hsa-miR-23b	661.41	38.00	118.07	9.12
hsa-miR-338	120.97	6.24	63.80	3.57
hsa-miR-339	567.25	29.29	136.13	7.21
hsa-miR-33	250.92	18.20	106.04	8.75
hsa-miR-324-5p	332.76	17.03	169.53	8.76
hsa-miR-330	12.90	1.32	2.75	1.31
hsa-miR-425-3p	8.89	1.24	2.28	1.33
hsa-miR-425-5p	229.77	13.03	107.43	9.24
hsa-miR-148b	64.18	4.35	40.85	3.08
hsa-miR-431	11.29	1.57	0.01	1.57
hsa-miR-296	28.73	2.22	9.50	1.66
hsa-miR-26b	87.88	5.09	46.50	3.04
hsa-miR-429	236.16	13.82	7.60	1.66
hsa-miR-421	23.42	1.89	10.80	1.68
hsa-miR-93	2069.31	128.70	316.16	19.76
hsa-miR-92b	55.05	3.45	27.43	2.09
hsa-miR-99b	125.21	7.04	63.87	3.88
hsa-miR-98	108.88	6.93	62.70	4.20
hsa-miR-615	10.53	1.18	2.06	1.13
hsa-miR-618	7.97	1.47	1.19	1.78
hsa-miR-590	98.20	4.77	49.05	2.67
hsa-miR-7	180.82	9.69	31.52	2.27
hsa-miR-497	19.65	1.84	6.49	1.64
hsa-miR-551a	9.90	1.50	5.65	1.66
hsa-miR-550	16.34	1.35	4.08	1.31
hsa-miR-565	298.68	18.95	143.48	9.19
hsa-miR-769-5p	67.38	3.66	30.61	2.21
hsa-miR-769-3p	24.19	1.38	13.45	1.29
hsa-miR-652	200.84	7.20	35.18	1.69
hsa-miR-505	5.59	1.04	2.95	1.17
hsa-miR-203	764.17	40.38	60.05	3.55
hsa-miR-210	157.44	8.15	70.01	3.95

hsa-miR-181c	16.67	1.70	8.12	1.64
hsa-miR-205	24.82	1.91	0.12	34.61
hsa-miR-196a	85.30	5.96	48.55	3.18
hsa-miR-200a	433.94	25.05	24.78	2.25
hsa-miR-181d	131.72	6.82	82.19	4.45
hsa-miR-195	97.48	5.66	55.89	3.61
hsa-miR-191	65.78	4.13	27.46	2.43
hsa-miR-181a	169.70	9.81	90.25	5.50
hsa-miR-189	5.51	1.47	0.01	1.57
hsa-miR-185	174.99	10.99	72.72	4.84
hsa-miR-181a*	23.13	1.40	14.68	1.28
hsa-miR-183	123.37	6.92	70.92	4.25
hsa-miR-181b	453.69	26.28	253.91	15.16
hsa-miR-301	790.45	45.03	438.17	24.79
hsa-miR-217	0.16	295.06	0.01	1.11
hsa-miR-299-3p	5.66	1.44	3.57	1.59
hsa-miR-542-5p	6.19	1.53	3.47	1.83
hsa-miR-493-5p	6.39	1.06	0.65	3.88
hsa-miR-489	6.55	1.48	0.06	1.58
hsa-miR-410	7.83	1.49	0.01	1.57
