# Supplementary material for: An integrative genomic analysis revealed the relevance of microRNA and gene expression for drug-resistance in human breast cancer cells
Source: Mol Cancer. 2011 Nov 3;10:135. doi: 10.1186/1476-4598-10-135 (PMC3247093; doi:10.1186/1476-4598-10-135)
Supplement: Additional file 8 — Table S4. List of miRNAs located in genome deletion regions in MCF7-ADR. [file 1476-4598-10-135-S8.PDF]

Table S4 List of miRNAs located in genome deletion regions in MCF7-ADR

Gene name	Delation	Aberration region
hsa-let-7a	-1.07	chr9:91275583-106849984
hsa-let-7d	-1.07	chr9:91275583-106849984
hsa-let-7f	-1.07	chr9:91275583-106849984
hsa-miR-105	-1.04	chrX:2710116-154442577
hsa-miR-106a	-1.04	chrX:2710116-154442577
hsa-miR-106b	-1.06	chr7:88230678-127989538
hsa-miR-122a	-1.03	chr18:32382054-76103396
hsa-miR-129	-1.06	chr7:88230678-127989538
hsa-miR-189	-1.07	chr9:91275583-106849984
hsa-miR-18b	-1.04	chrX:2710116-154442577
hsa-miR-20b	-1.04	chrX:2710116-154442577
hsa-miR-220	-1.04	chrX:2710116-154442577
hsa-miR-224	-1.04	chrX:2710116-154442577
hsa-miR-23b	-1.07	chr9:91275583-106849984
hsa-miR-24	-1.07	chr9:91275583-106849984
hsa-miR-25	-1.06	chr7:88230678-127989538
hsa-miR-27b	-1.07	chr9:91275583-106849984
hsa-miR-363	-1.04	chrX:2710116-154442577
hsa-miR-363*	-1.04	chrX:2710116-154442577
hsa-miR-424	-1.04	chrX:2710116-154442577
hsa-miR-448	-1.04	chrX:2710116-154442577
hsa-miR-450	-1.04	chrX:2710116-154442577
hsa-miR-452	-1.04	chrX:2710116-154442577
hsa-miR-452*	-1.04	chrX:2710116-154442577
hsa-miR-489	-1.06	chr7:88230678-127989538
hsa-miR-503	-1.04	chrX:2710116-154442577
hsa-miR-504	-1.04	chrX:2710116-154442577
hsa-miR-505	-1.04	chrX:2710116-154442577
hsa-miR-506	-1.04	chrX:2710116-154442577
hsa-miR-507	-1.04	chrX:2710116-154442577
hsa-miR-508	-1.04	chrX:2710116-154442577
hsa-miR-509	-1.04	chrX:2710116-154442577
hsa-miR-510	-1.04	chrX:2710116-154442577
hsa-miR-513	-1.04	chrX:2710116-154442577
hsa-miR-514	-1.04	chrX:2710116-154442577
hsa-miR-542-3p	-1.04	chrX:2710116-154442577
hsa-miR-542-5p	-1.04	chrX:2710116-154442577
hsa-miR-551b	-5.04	chr3:163490512-170718270
hsa-miR-565	-1.08	chr3:45453353-45736276
hsa-miR-591	-1.06	chr7:88230678-127989538
hsa-miR-592	-1.06	chr7:88230678-127989538
hsa-miR-593	-1.06	chr7:88230678-127989538
hsa-miR-623	-1.02	chr13:97699665-114107778
hsa-miR-652	-1.04	chrX:2710116-154442577
hsa-miR-653	-1.06	chr7:88230678-127989538
hsa-miR-766	-1.04	chrX:2710116-154442577
hsa-miR-767-3p	-1.04	chrX:2710116-154442577
hsa-miR-767-5p	-1.04	chrX:2710116-154442577
hsa-miR-93	-1.06	chr7:88230678-127989538
