# Supplementary material for: An integrative genomic analysis revealed the relevance of microRNA and gene expression for drug-resistance in human breast cancer cells
Source: Mol Cancer. 2011 Nov 3;10:135. doi: 10.1186/1476-4598-10-135 (PMC3247093; doi:10.1186/1476-4598-10-135)
Supplement: Additional file 10 — Table S5. List of up-regulated genes in MCF7-ADR judged by T-test in comparison with MCF7. [file 1476-4598-10-135-S10.PDF]

Table S5 List of up-regulated genes in MCF7-ADR judged by T-test in comparison with MCF7

ID	MCF7		MCF7-ADR			
Systematic	Normalized	StdErr Norm	Normalized	StdErr Norm	Genbank	Gene Symbol
A_23_P159986	1.12	0.08	14.22	1.28	BC007360	
A_24_P921086	0.01	0.01	0.43	0.06		
A_24_P299318	0.41	0.03	18.71	2.35	BC014203	FAM101B
A_23_P135381	0.16	0.03	3.65	0.40	NM_001003845	SP5
A_24_P283341	0.63	0.05	2.46	0.17	NM_022765	MICAL1
A_23_P353541	0.01	0.01	0.05	0.01	BC069363	DLX6
A_23_P62115	2.26	0.18	9.99	0.71	NM_003254	TIMP1
A_23_P108170	0.05	0.01	4.99	0.80	NM_002782	PSG6
A_24_P13190	0.01	0.01	0.11	0.01	NM_138961	ESAM
A_24_P350838	0.01	0.01	0.26	0.03	X62009	FBN2
A_23_P401547	0.01	0.01	3.43	0.28	NM_015480	PVRL3
A_23_P207367	0.02	0.01	0.38	0.06	NM_003152	STAT5A
A_23_P345139	4.29	0.30	13.52	0.96	NM_174983	C19orf28
A_23_P327910	0.06	0.01	0.22	0.02	NM_003413	ZIC3
A_23_P144458	0.14	0.02	2.39	0.17	NM_001221	CAMK2D
A_32_P30760	0.13	0.01	0.63	0.05	BM727690	
A_23_P207387	0.21	0.02	0.79	0.06	NM_032484	GHDC
A_23_P146554	0.26	0.02	6.02	0.48	NM_000954	PTGDS
A_24_P389608	0.03	0.01	0.50	0.07	NM_153256	C10orf47
A_32_P185628	0.01	0.01	0.07	0.01		PRNP
A_23_P143274	0.28	0.02	1.56	0.11	NM_024958	NRSN2
A_23_P350249	0.01	0.01	0.29	0.02	AB065086	GLIS3
A_23_P43490	0.86	0.06	271.63	23.37	NM_058197	CDKN2A
A_23_P23457	0.06	0.01	0.24	0.02	NM_017556	FBLIM1
A_23_P119196	2.40	0.17	44.77	4.53	NM_016270	KLF2
A_23_P81640	0.01	0.01	0.09	0.01	NM_052909	
A_32_P141013	0.19	0.02	12.32	0.89	AB029033	IQSEC3
A_23_P84929	0.06	0.01	13.65	0.97	NM_033518	SLC38A5
A_23_P51646	0.76	0.05	2.73	0.19	NM_004073	PLK3
A_32_P161681	0.38	0.05	3.75	0.49	NM_001008528	MXRA7
A_23_P50338	2.13	0.15	23.88	2.45	NM_006087	TUBB4
A_23_P201386	2.68	0.20	11.46	0.97	NM_012137	DDAH1
A_23_P40611	0.01	0.01	0.23	0.02	NM_000355	TCN2
A_32_P77225	0.03	0.01	0.08	0.01		
A_23_P17837	0.01	0.01	0.17	0.02	NM_145344	APOL1
A_23_P388812	0.69	0.07	4.63	0.52	NM_152515	CKAP2L
A_23_P85716	0.04	0.01	0.10	0.01	NM_021642	FCGR2A
A_24_P183128	0.18	0.02	16.01	1.13	NM_016619	PLAC8
A_23_P320185	27.59	1.95	90.45	6.40	NM_175614	NDUFA11
A_24_P83158	2.76	0.20	9.55	0.68	NR_002184	
A_23_P43238	0.46	0.03	5.85	0.49	NM_145201	NAPRT1
A_23_P131754	0.20	0.02	1.28	0.09	NM_024059	C20orf195
A_23_P130995	0.90	0.06	12.93	0.91	NM_144779	FXYD5
A_23_P21063	0.01	0.01	0.29	0.04	NM_198056	SCN5A
A_23_P301803	0.06	0.01	0.60	0.07		
A_23_P396981	0.05	0.01	0.22	0.02	NM_001012506	CCDC66
A_23_P70746	0.05	0.01	0.19	0.02	NM_017651	AHI1
A_23_P391586	13.32	0.94	60.70	5.15	NM_001018004	TPM1
A_23_P391906	0.01	0.01	4.71	0.44	BC044246	KIAA1913
A_23_P373100	0.01	0.01	0.73	0.08	BC020879	
A_23_P58002	0.46	0.03	1.73	0.12	NM_022171	TCTA
A_23_P421423	0.70	0.05	9.77	0.91	NM_006291	TNFAIP2
A_23_P34233	10.28	0.73	34.41	2.43	NM_014298	QPRT
A_23_P213699	0.01	0.01	0.13	0.02	NM_013982	NRG2
A_32_P107372	0.01	0.01	0.55	0.08	NM_002053	GBP1
A_23_P301304	0.73	0.05	5.80	0.41	NM_023109	
A_24_P380734	0.51	0.04	5.00	0.53	NM_002998	SDC2
A_32_P168701	0.09	0.01	3.85	0.42		
A_24_P77432	0.01	0.01	1.31	0.19	NM_133631	ROBO1
A_23_P315451	0.02	0.01	1.85	0.21	NM_199180	KIRREL2
A_24_P47182	1.55	0.11	15.99	1.56	NM_014000	VCL
A_23_P142075	0.32	0.02	2.25	0.23	NM_001611	ACP5
A_23_P212649	0.01	0.01	0.37	0.04	AK094187	

A_32_P81357	6.43	0.46	23.53	1.66	NM_016044	FAHD2A
A_24_P817209	0.01	0.01	0.20	0.02	AK125077	
A_32_P184937	1.40	0.12	6.97	0.55	BU678941	
A_23_P348264	0.06	0.01	1.17	0.08	NM_144652	LETM2
A_23_P120325	0.68	0.05	2.71	0.20	NM_002254	KIF3C
A_23_P203299	4.01	0.28	14.88	1.08	NM_002901	RCN1
A_23_P115046	0.74	0.05	2.96	0.21	NM_020365	EIF2B3
A_23_P127584	0.01	0.01	73.75	5.88	NM_006169	NNMT
A_23_P410507	0.13	0.01	0.40	0.03	NM_004158	PSPN
A_32_P109817	0.32	0.04	2.40	0.22	NM_006007	ZFAND5
A_23_P122216	0.08	0.01	0.90	0.11	NM_002317	LOX
A_23_P121637	0.04	0.01	2.96	0.33	NM_003619	PRSS12
A_24_P402690	3.02	0.34	28.52	2.23	NM_030926	ITM2C
A_32_P217655	9.14	0.65	38.64	3.09	XM_496405	
A_24_P506977	3.84	0.28	14.32	1.01	AK096179	
A_23_P37988	1.65	0.16	9.34	0.75	NM_152727	CPNE2
A_24_P84834	0.02	0.01	0.23	0.03	NM_201444	DGKA
A_23_P134454	0.17	0.01	14.31	1.67	NM_001753	CAV1
A_23_P334955	0.08	0.01	0.58	0.04	NM_053279	C8orf13
A_23_P325924	0.24	0.03	1.90	0.22	AK090454	FAM59B
A_23_P100711	5.07	0.37	20.28	1.59	NM_000304	PMP22
A_32_P4349	0.01	0.01	0.07	0.01		FNDC3B
A_32_P12562	0.01	0.01	0.38	0.03	BX648855	
A_23_P149019	0.27	0.02	1.02	0.07	NM_001703	BAI2
A_23_P119390	0.23	0.02	0.61	0.04	NM_014601	EHD2
A_23_P26994	0.01	0.01	9.38	0.98	NM_031498	GNGT2
A_23_P153651	1.18	0.08	6.64	0.47	NM_024333	FSD1
A_23_P65659	0.01	0.01	0.11	0.01	NM_024490	ATP10A
A_32_P203408	0.03	0.01	1.29	0.12	XM_374101	
A_23_P320739	0.03	0.01	0.33	0.04	NM_002397	MEF2C
A_23_P321846	0.01	0.01	0.61	0.05	NM_002251	KCNS1
A_23_P129956	1.76	0.12	5.77	0.41	NM_004090	DUSP3
A_24_P88801	0.37	0.04	2.82	0.32	NM_000272	NPHP1
A_23_P34548	0.03	0.01	0.20	0.02	NM_006642	SDCCAG8
A_23_P321377	0.23	0.02	0.59	0.04	NM_152374	
A_32_P229132	0.01	0.01	0.31	0.03	NM_020066	FMN2
A_23_P165343	1.40	0.12	8.34	0.83	NM_002830	PTPN4
A_23_P202773	5.40	0.38	28.37	2.18	NM_016611	
A_23_P24926	4.14	0.29	20.39	1.77	NM_002027	FNTA
A_23_P120504	0.04	0.01	1.56	0.12	NM_018354	C20orf46
A_23_P129433	0.79	0.06	2.77	0.21	NM_004594	SLC9A5
A_24_P215765	0.01	0.01	0.46	0.07	NM_024490	ATP10A
A_24_P88583	0.08	0.01	0.66	0.07	AK094353	
A_23_P128598	0.25	0.02	1.67	0.12	NM_079836	TUBA3C
A_24_P698136	0.95	0.07	2.55	0.18	AK125299	ACOT7
A_32_P201897	0.01	0.01	1.24	0.09	NM_020066	FMN2
A_23_P156408	0.01	0.01	0.13	0.02	NM_024882	
A_24_P342511	0.04	0.01	0.12	0.01	NM_152497	
A_23_P59637	0.02	0.01	0.21	0.03	AB018259	DOCK4
A_32_P207420	0.38	0.03	0.77	0.06		
A_23_P154065	1.96	0.14	34.09	3.46	NM_006000	TUBA4A
A_23_P130509	0.01	0.01	0.15	0.02	NM_014518	ZNF229
A_24_P372223	0.01	0.01	0.14	0.02	NM_138715	MSR1
A_24_P48898	1.37	0.10	4.08	0.29	NM_145637	APOL2
A_24_P152527	0.42	0.03	1.54	0.11	NM_006373	VAT1
A_23_P114947	0.11	0.01	2.02	0.17	NM_002923	RGS2
A_32_P59737	0.02	0.01	0.44	0.07		
A_24_P404033	0.23	0.03	1.73	0.13	NM_182616	C15orf38
A_23_P82065	0.02	0.01	0.39	0.04	NM_022726	ELOVL4
A_23_P50786	0.07	0.01	5.29	0.37	NM_015526	CLIP3
A_23_P61140	0.10	0.01	0.33	0.02	NM_006480	RGS14
A_24_P136641	0.63	0.04	1.58	0.11	BC013655	
A_24_P274795	7.38	0.54	26.39	2.02	NM_018719	CDCA7L
A_23_P111126	0.04	0.01	1.58	0.11	L06175	HCP5
A_23_P406122	0.16	0.01	0.59	0.04	AL110249	
A_23_P314526	1.26	0.09	3.92	0.28	NM_005803	FLOT1

A_24_P27412	0.94	0.07	3.01	0.21	NM_005701	SNUPN
A_23_P26223	3.99	0.28	11.30	0.80	NM_000048	ASL
A_24_P918762	0.09	0.01	0.36	0.03		
A_23_P201901	0.01	0.01	8.13	0.66	NM_001001957	OR2W3
A_23_P29303	0.78	0.06	2.74	0.21	NM_015703	
A_32_P69849	0.79	0.06	2.64	0.19	NM_001012978	NGFRAP1L1
A_23_P49792	0.01	0.01	0.07	0.01	NM_178500	PHOSPHO1
A_32_P103131	1.14	0.08	6.27	0.57		
A_32_P143824	0.23	0.02	2.80	0.30		PTRF
A_23_P347623	0.04	0.01	0.36	0.05	NM_019891	ERO1LB
A_23_P160968	0.01	0.01	0.94	0.09	NM_018891	LAMC2
A_23_P320261	13.46	0.95	65.85	5.89	NM_033317	DMKN
A_23_P351679	0.54	0.04	1.32	0.09	NM_004614	TK2
A_23_P435394	0.02	0.01	0.09	0.01	NM_000958	PTGER4
A_23_P7684	0.05	0.01	1.26	0.13	AK131381	CCNJL
A_23_P391264	0.62	0.04	2.03	0.14	NM_007047	BTN3A2
A_23_P344555	0.04	0.01	0.70	0.13	NM_006403	NEDD9
A_23_P76882	10.51	0.74	35.25	2.62	NM_021178	CCNB1IP1
A_24_P61490	0.03	0.01	6.06	0.73	NM_003317	TITF1
A_24_P15292	0.30	0.03	1.74	0.15	NM_032018	C1orf124
A_23_P24870	5.31	0.31	40.97	3.93	NM_000610	CD44
A_32_P425998	0.04	0.01	1.42	0.10	NM_178275	
A_23_P62807	3.43	0.28	42.13	5.27	NM_016002	SCCPDH
A_24_P85942	0.48	0.04	2.33	0.20	NM_181453	GCC2
A_24_P416346	0.08	0.01	1.08	0.08	NM_001986	ETV4
A_23_P135494	4.54	0.32	49.26	5.12	NM_013943	CLIC4
A_23_P372946	0.01	0.01	0.21	0.03	NM_138461	TM4SF19
A_23_P371933	0.01	0.01	0.12	0.01	BC012536	HSD17B12
A_23_P36562	0.08	0.01	1.54	0.11	NM_002205	ITGA5
A_23_P256413	0.55	0.04	7.98	0.68	NM_138410	CMTM7
A_24_P43876	0.67	0.06	3.50	0.35	NM_017998	C9orf40
A_23_P214176	0.22	0.02	3.27	0.43	NM_133493	CD109
A_32_P154361	0.01	0.01	0.35	0.05		
A_23_P120467	1.14	0.08	11.46	1.19	NM_199427	ZFP64
A_23_P3866	13.81	0.98	85.05	7.27	NM_021149	COTL1
A_23_P161481	0.01	0.01	0.46	0.06	NM_014431	KIAA1274
A_23_P139919	0.28	0.02	1.48	0.14	NM_018413	CHST11
A_32_P108666	0.01	0.01	0.56	0.08	AK091731	
A_23_P428819	5.28	0.46	23.35	1.90	NM_018064	C6orf166
A_23_P169351	0.01	0.01	0.25	0.03	NM_003026	SH3GL2
A_23_P96158	0.58	0.04	1.32	0.09	NM_000422	KRT17
A_23_P9415	0.76	0.05	3.67	0.29	NM_002197	ACO1
A_24_P169048	0.03	0.01	0.18	0.02	NR_001450	RFPL3S
A_23_P87461	0.79	0.06	2.47	0.17	NM_001478	B4GALNT1
A_24_P314681	0.04	0.01	0.09	0.01	BX281397	
A_32_P524014	0.37	0.03	2.40	0.26	AK023675	UTRN
A_32_P102627	0.01	0.01	0.10	0.01		
A_23_P142055	0.34	0.03	0.80	0.06	XM_172995	
A_23_P124084	1.91	0.14	61.57	6.75	NM_005576	LOXL1
A_23_P210164	0.01	0.01	0.58	0.08	NM_019558	HOXD8
A_24_P98975	1.33	0.09	4.66	0.35	NM_033121	ANKRD13A
A_24_P390096	0.04	0.01	0.43	0.03	U16307	GLIPR1
A_23_P76102	1.77	0.13	6.57	0.47	NM_005811	GDF11
A_23_P362893	1.60	0.14	7.38	0.63	NM_021961	TEAD1
A_23_P101908	0.12	0.02	2.78	0.23	NM_005883	APC2
A_23_P36825	0.36	0.03	1.72	0.16	NM_003979	GPRC5A
A_23_P145965	0.89	0.11	8.28	1.10	NM_003596	TPST1
A_23_P161624	0.04	0.01	3.57	0.25	NM_005438	FOSL1
A_23_P215341	0.50	0.05	2.57	0.19	NM_017946	FKBP14
A_24_P355816	1.62	0.11	6.02	0.46	NM_018370	
A_32_P176675	0.06	0.01	0.44	0.04	NM_145269	
A_24_P199929	0.04	0.01	0.11	0.01	NM_016516	VPS54
A_23_P342869	0.01	0.01	1.67	0.15	NM_020066	FMN2
A_32_P2354	1.19	0.08	4.30	0.30	BX508036	
A_24_P278167	1.71	0.19	12.46	1.46	NM_006007	ZFAND5
A_24_P724886	0.02	0.01	0.29	0.04	BQ437598	

A_32_P63886	0.10	0.01	0.36	0.03		CD59
A_32_P16954	0.01	0.01	0.07	0.01	AF075028	
A_23_P122815	1.11	0.09	7.26	0.51	NM_001219	CALU
A_23_P8108	0.86	0.07	3.08	0.22	NM_002123	HLA-DQB1
A_23_P218770	0.36	0.03	14.07	0.99	NM_002872	RAC2
A_24_P579356	0.01	0.01	0.15	0.02	NM_001010000	ARHGAP28
A_23_P357207	0.01	0.01	0.39	0.03	NM_138409	C6orf117
A_23_P431319	5.01	0.41	29.64	3.08	NM_173834	YIPF6
A_23_P381261	0.06	0.01	0.15	0.01	NM_139247	ADCY4
A_23_P129133	0.01	0.01	0.14	0.02	NM_000275	OCA2
A_23_P163467	1.20	0.09	26.32	2.98	NM_207380	
A_23_P819	6.03	0.43	18.53	1.31	NM_005101	ISG15
A_24_P309415	4.10	0.34	21.21	1.67	NM_052932	TMEM123
A_23_P44436	0.01	0.01	0.07	0.01	NM_019617	GKN1
A_23_P254741	0.09	0.01	2.45	0.19	NM_003102	SOD3
A_23_P66038	0.04	0.01	0.18	0.01	NM_013304	ZDHHC1
A_24_P388940	0.07	0.01	0.77	0.10	NM_020425	C6orf162
A_23_P58706	0.01	0.01	0.11	0.01	AK001520	
A_24_P50245	0.32	0.03	2.91	0.21	NM_006120	HLA-DMA
A_23_P422212	0.01	0.01	4.60	0.67	NM_173508	SLC35F3
A_23_P118536	0.01	0.01	0.38	0.05	NM_018042	SLFN12
A_23_P79458	0.27	0.02	0.72	0.05	NM_001009812	LBX2
A_23_P67785	0.01	0.01	0.41	0.05	NM_024532	SPAG16
A_23_P210763	0.06	0.01	0.51	0.05	NM_000214	JAG1
A_24_P77904	0.01	0.01	0.11	0.01	NM_018951	HOXA10
A_23_P77731	0.53	0.05	3.45	0.24	NM_001888	CRYM
A_23_P342744	0.06	0.01	0.67	0.08	NM_153713	LIX1L
A_32_P46571	0.03	0.01	0.76	0.09	NM_017821	RHBDL2
A_32_P143850	0.23	0.02	0.85	0.06	BC024198	
A_23_P149270	0.08	0.01	1.14	0.10		CLCNKB
A_23_P104798	0.04	0.01	9.68	0.73	NM_001562	IL18
A_23_P90359	0.80	0.06	3.44	0.26	NM_004558	NRTN
A_23_P20443	0.01	0.01	1.15	0.17	NM_021020	LZTS1
A_32_P10403	0.01	0.01	0.57	0.07		
A_23_P130376	0.06	0.01	1.12	0.09	NM_022068	FAM38B
A_23_P396328	0.19	0.02	1.18	0.10	AK098775	MPDZ
A_23_P21644	2.83	0.21	26.42	2.74	NM_016245	HSD17B11
A_24_P165949	0.02	0.01	0.30	0.03	NM_021101	CLDN1
A_23_P429449	0.01	0.01	0.05	0.01	NM_033119	NKD1
A_23_P8582	0.08	0.01	0.73	0.07	NM_032581	FAM126A
A_24_P85099	0.01	0.01	0.17	0.02	NM_003483	
A_24_P116710	0.01	0.01	0.06	0.01	NM_005854	RAMP2
A_23_P90804	0.17	0.02	0.76	0.05	NM_145686	MAP4K4
A_24_P108779	0.32	0.02	1.35	0.10	NM_024681	KCTD17
A_23_P250102	0.02	0.01	12.68	1.37	AB014567	CAND2
A_23_P3295	0.13	0.01	0.63	0.05	AL110257	
A_23_P36753	0.10	0.01	1.24	0.14	NM_000690	ALDH2
A_23_P343954	0.02	0.01	2.22	0.22	NM_006546	IGF2BP1
A_23_P254271	51.55	3.65	343.06	30.05	NM_032525	TUBB6
A_23_P108075	0.01	0.01	1.11	0.16	NM_019849	SLC7A10
A_23_P75989	0.23	0.02	4.77	0.55	NM_002576	PAK1
A_23_P200096	0.12	0.01	0.44	0.03	NM_025106	SPSB1
A_23_P11980	0.02	0.01	0.67	0.05	XM_496361	
A_32_P220696	0.32	0.02	1.12	0.08	NM_017489	TERF1
A_23_P356554	1.68	0.14	9.60	0.84	NM_004282	BAG2
A_32_P305020	0.01	0.01	0.27	0.03	AK074383	
A_23_P70983	0.25	0.02	5.02	0.36	AB011131	
A_32_P103695	0.21	0.02	2.02	0.23	NM_145269	
A_24_P943781	0.01	0.01	0.08	0.01	NM_024913	
A_24_P344087	0.26	0.03	13.72	0.98	NM_005132	REC8
A_23_P151805	0.04	0.01	0.61	0.09	NM_006329	FBLN5
A_23_P37244	0.31	0.03	2.82	0.30	NM_003082	SNAPC1
A_23_P38894	0.93	0.07	3.70	0.26	NM_018381	
A_23_P28999	0.01	0.01	0.47	0.06	AK025855	CDH4
A_23_P118967	0.01	0.01	1.80	0.27	NM_005559	LAMA1
A_23_P43630	0.01	0.01	0.08	0.01	AK023447	

A_24_P28739	0.01	0.01	0.12	0.01	NM_018084	CCDC88A
A_23_P153185	0.02	0.01	0.44	0.06	BC012609	SERPINB2
A_32_P23517	0.11	0.01	0.36	0.03	BU753102	
A_23_P89871	0.01	0.01	0.91	0.11	NM_018355	ZNF415
A_24_P232365	0.01	0.01	0.92	0.07	NM_019043	APBB1IP
A_23_P65000	1.49	0.11	6.46	0.46	NM_013300	C12orf24
A_23_P29347	0.65	0.05	2.91	0.21	NM_002969	MAPK12
A_23_P328206	0.53	0.04	2.34	0.17	AB023227	DNMBP
A_24_P101282	0.07	0.01	1.02	0.07	BC031342	
A_23_P418199	0.37	0.03	2.98	0.27	BC037171	CCL27
A_24_P207139	0.14	0.01	0.29	0.02	NM_033238	PML
A_24_P392110	0.01	0.01	4.69	0.68	NM_182707	PSG8
A_23_P91802	2.76	0.20	8.49	0.60	NM_001953	ECGF1
A_23_P35906	0.88	0.06	74.16	7.34	NM_033306	CASP4
A_24_P294343	0.01	0.01	0.14	0.01	NM_024616	C3orf52
A_24_P303420	0.01	0.01	0.30	0.04	NM_001010871	
A_23_P431305	3.73	0.26	11.77	0.83	NM_152421	FAM69B
A_23_P303251	0.01	0.01	0.26	0.03	NM_173495	PTCHD1
A_23_P169934	2.81	0.20	14.73	1.17	NM_178314	
A_32_P62863	0.30	0.03	15.58	2.31	NM_014575	SCHIP1
A_23_P80068	1.46	0.10	32.27	3.73	NM_006806	BTG3
A_23_P358548	0.01	0.01	3.44	0.37	NM_199329	SLC43A3
A_24_P925040	0.01	0.01	0.06	0.01	NM_001233	CAV2
A_24_P68991	0.01	0.01	0.14	0.01	NM_006168	NKX6-1
A_23_P139864	0.02	0.01	0.15	0.02	NM_031289	GSG1
A_23_P219105	0.34	0.02	1.70	0.13	NM_023111	
A_23_P377318	0.09	0.01	0.38	0.03	NM_153221	CILP2
A_23_P80503	0.01	0.01	0.10	0.01	NM_133631	ROBO1
A_24_P827037	0.01	0.01	0.19	0.02	NM_130830	LRRC15
A_23_P324340	0.47	0.04	3.55	0.36	NM_033510	DISP2
A_24_P941459	0.06	0.01	0.17	0.01	AK022156	CRSP6
A_23_P104881	0.02	0.01	0.41	0.03	NM_019055	ROBO4
A_23_P343935	1.15	0.09	11.60	1.23	NM_022051	EGLN1
A_23_P163455	0.05	0.01	0.55	0.04	NM_002373	MAP1A
A_32_P77252	0.05	0.01	0.56	0.05		
A_23_P103775	0.01	0.01	0.16	0.02	NM_032270	LRRC8C
A_23_P160582	1.55	0.13	10.55	0.75	NM_031207	HYI
A_24_P940426	0.01	0.01	0.18	0.01	NM_206855	QKI
A_23_P306346	0.01	0.01	0.05	0.01	NM_152420	C9orf41
A_24_P466102	0.04	0.01	0.12	0.01		
A_32_P117760	0.15	0.02	1.35	0.11	BG208131	
A_23_P390097	0.10	0.01	0.60	0.04	NM_152574	C9orf52
A_24_P174353	0.01	0.01	0.19	0.02		
A_23_P109427	0.01	0.01	1.40	0.12	NM_000854	GSTT2
A_24_P652609	0.01	0.01	0.63	0.05	AK054645	
A_23_P215819	0.01	0.01	0.64	0.06	NM_001009957	
A_23_P468	0.01	0.01	0.41	0.05	NM_021179	C1orf114
A_24_P204135	0.93	0.07	2.89	0.20		
A_23_P22205	0.01	0.01	0.12	0.01	NM_003759	SLC4A4
A_23_P258190	0.06	0.01	39.78	4.57	NM_001628	AKR1B1
A_23_P218555	0.66	0.05	2.08	0.15	NM_005253	FOSL2
A_23_P215517	1.26	0.09	5.42	0.38	BC009555	KLHL7
A_23_P62081	0.01	0.01	0.11	0.01	NM_003020	SCG5
A_24_P144543	0.01	0.01	0.06	0.01	XM_371677	
A_24_P235520	0.02	0.01	0.30	0.03	NM_175923	
A_32_P160561	0.01	0.01	0.23	0.03	NM_152721	DOK6
A_24_P98948	0.37	0.03	3.88	0.27		
A_24_P788878	0.01	0.01	0.07	0.01	BM054849	
A_23_P253446	0.01	0.01	0.11	0.01	NM_002045	GAP43
A_32_P212886	0.21	0.03	2.99	0.31	BC014117	TBXAS1
A_23_P127525	0.01	0.01	0.59	0.08	NM_005238	ETS1
A_32_P103291	5.57	0.39	40.76	3.74	NM_022743	SMYD3
A_23_P98645	0.01	0.01	0.25	0.02	NM_003737	DCHS1
A_23_P154667	2.17	0.15	6.84	0.48	NM_000801	FKBP1A
A_23_P91910	0.01	0.01	0.13	0.01	NM_020353	PLSCR4
A_23_P87401	0.90	0.07	4.65	0.33	NM_030792	GDPD5

A_23_P37785	0.01	0.01	0.91	0.08	BC070103	
A_23_P39304	0.01	0.01	1.18	0.17	NM_002784	PSG9
A_23_P377965	0.01	0.01	0.35	0.03	BC010538	
A_32_P166693	0.10	0.01	6.14	0.53		
A_23_P258769	0.02	0.01	3.20	0.36	NM_002121	HLA-DPB1
A_32_P22401	4.60	0.33	21.34	1.66	NM_018067	MAP7D1
A_32_P202977	0.01	0.01	0.35	0.03		
A_23_P156708	1.18	0.08	22.05	2.48	NM_019105	TNXB
A_23_P50498	46.23	3.27	118.16	8.36	NM_000146	FTL
A_23_P28466	0.01	0.01	0.54	0.08	NM_178821	WDR69
A_23_P70060	0.47	0.03	4.20	0.43	NM_176895	PPAP2A
A_23_P401774	0.01	0.01	0.95	0.08	NM_018712	ELMOD1
A_23_P24843	0.21	0.03	4.11	0.54	NM_014632	MICAL2
A_24_P926580	0.01	0.01	0.29	0.04	AK001808	
A_23_P405267	0.48	0.04	2.50	0.23	AK057922	CDH24
A_32_P133840	0.19	0.02	0.66	0.05	NM_014858	TMCC2
A_24_P284584	0.01	0.01	0.17	0.02	NM_032497	ZNF559
A_23_P39074	8.37	0.59	37.33	2.64	NM_006270	RRAS
A_23_P113656	0.07	0.01	0.30	0.02	NM_006087	TUBB4
A_23_P167051	0.01	0.01	1.02	0.13	NM_014556	
A_23_P10172	0.01	0.01	0.15	0.01	NM_013270	
A_23_P166686	7.13	0.50	167.34	16.51	NM_016201	AMOTL2
A_23_P345460	2.50	0.18	12.70	1.09	NM_015432	PLEKHG4
A_23_P94230	0.01	0.01	0.29	0.02	NM_015364	LY96
A_24_P4171	0.20	0.02	1.14	0.09	NM_023111	
A_32_P139196	0.20	0.02	0.71	0.05	NM_213723	
A_24_P303454	0.13	0.02	2.38	0.21	NM_012454	TIAM2
A_23_P83976	0.01	0.01	0.07	0.01	NM_145036	CCDC46
A_23_P363344	1.44	0.12	12.05	1.31	NM_000366	TPM1
A_23_P142294	3.27	0.23	10.39	0.73	NM_014297	ETHE1
A_23_P20122	0.16	0.01	0.71	0.06	NM_024625	ZC3HAV1
A_23_P208788	12.72	0.90	166.86	15.09	NM_033520	C19orf33
A_23_P78762	0.71	0.05	2.14	0.15	NM_016246	HSD17B14
A_23_P87013	0.18	0.01	1.46	0.10	NM_001001522	TAGLN
A_23_P34382	0.01	0.01	0.05	0.01	NM_004921	CLCA3
A_23_P259442	0.50	0.05	3.89	0.43	NM_001873	CPE
A_23_P111888	0.52	0.04	7.41	0.91	NM_138455	CTHRC1
A_24_P48177	0.36	0.03	2.08	0.17	AK127322	ST3GAL2
A_23_P126186	2.89	0.20	17.12	1.52	NM_003676	DEGS1
A_24_P224966	0.02	0.01	0.18	0.02	NM_000085	CLCNKB
A_23_P59582	0.29	0.02	8.79	1.10	NM_012431	SEMA3E
A_23_P218879	0.63	0.05	3.66	0.29	NM_016381	TREX1
A_24_P7642	0.23	0.02	2.24	0.26	NM_001444	FABP5
A_23_P145916	0.03	0.01	3.91	0.28	NM_001129	AEBP1
A_24_P133584	0.10	0.01	1.16	0.09	NM_005928	MFGE8
A_32_P44047	0.01	0.01	0.11	0.01	BX415272	CD44
A_24_P51909	0.26	0.02	1.17	0.09	NM_006651	CPLX1
A_24_P418203	0.01	0.01	0.29	0.03	NM_033655	CNTNAP3
A_23_P36745	0.84	0.07	8.49	0.95	NM_000690	ALDH2
A_23_P313550	0.24	0.02	1.52	0.14	NM_173637	SLC25A41
A_23_P500034	0.01	0.01	0.81	0.06	NM_030379	GLI2
A_32_P220591	0.15	0.01	0.79	0.06		
A_23_P5983	0.14	0.01	5.16	0.37	NM_006227	PLTP
A_32_P70315	0.01	0.01	0.27	0.03	NM_003256	TIMP4
A_23_P216489	0.01	0.01	5.31	0.70	NM_005476	GNE
A_24_P134235	0.36	0.03	1.01	0.07	NM_003685	KHSRP
A_23_P134953	0.33	0.03	4.11	0.43	NM_001122	ADFP
A_23_P153827	0.07	0.01	0.25	0.02	NM_005934	MLLT1
A_23_P354798	0.66	0.05	1.99	0.14	NM_144576	COQ10A
A_24_P415208	0.31	0.04	3.74	0.33	BC060806	
A_24_P706340	0.01	0.01	0.89	0.12	L10374	
A_23_P66355	3.09	0.22	12.71	0.90	NM_000213	ITGB4
A_23_P84974	0.01	0.01	0.08	0.01		
A_24_P418044	7.63	0.37	56.30	4.45	BC062324	
A_23_P93442	1.21	0.10	6.83	0.62	NM_015278	SASH1
A_24_P329065	0.04	0.01	0.36	0.03	NM_007048	BTN3A1

A_23_P87528	0.01	0.01	0.49	0.07	NM_005504	BCAT1
A_23_P12965	0.18	0.02	1.60	0.14	NM_002033	FUT4
A_23_P14754	0.02	0.01	0.35	0.05	NM_178232	HAPLN3
A_23_P88589	5.16	0.36	27.80	2.43	NM_021005	NR2F2
A_23_P50504	33.29	2.35	100.25	7.09	NM_000146	FTL
A_23_P68031	0.01	0.01	0.09	0.01	NM_003151	STAT4
A_23_P90601	2.62	0.19	9.81	0.69	NM_182915	STEAP3
A_32_P70983	0.01	0.01	0.12	0.01	AK021689	
A_23_P54079	2.55	0.18	7.15	0.51	NM_017807	OSGEP
A_23_P125579	0.86	0.06	3.00	0.21	NM_021242	MID1IP1
A_23_P410159	0.05	0.01	0.15	0.01	NM_178422	PAQR7
A_23_P380076	0.25	0.02	1.39	0.12	AK055693	
A_23_P22422	1.16	0.08	45.56	5.26	NM_013364	PNMA3
A_32_P12104	0.02	0.01	0.11	0.01	NM_022662	ANAPC1
A_24_P134392	0.39	0.03	4.54	0.46	NM_006948	STCH
A_24_P225907	0.44	0.03	4.79	0.51	AK092040	DPH3
A_24_P345948	3.23	0.23	22.04	2.02	NM_001012732	DCTD
A_23_P257335	0.21	0.02	2.69	0.28	NM_006558	KHDRBS3
A_23_P46412	0.16	0.01	0.50	0.04	NM_002978	SCNN1D
A_24_P107695	10.86	0.77	57.34	4.28	NM_001102	ACTN1
A_23_P370651	0.10	0.01	0.62	0.06	NM_014883	FAM13A1
A_24_P307653	0.01	0.01	0.32	0.04	NM_182767	SLC6A15
A_32_P123514	0.44	0.04	1.74	0.17	BX648831	
A_32_P103945	0.66	0.06	2.50	0.18	NM_212543	B4GALT4
A_32_P79483	0.01	0.01	0.48	0.06	BC033993	
A_23_P316741	7.22	0.51	19.20	1.36	NM_003271	TSPAN4
A_23_P39766	0.32	0.02	0.87	0.06	NM_014905	GLS
A_32_P125338	0.01	0.01	1.11	0.14	NM_207334	FAM43B
A_23_P376449	0.91	0.06	2.39	0.17	XM_497240	
A_23_P69267	0.69	0.05	2.14	0.17	NM_015407	ABHD14A
A_23_P409093	0.01	0.01	0.38	0.04	NM_178826	TMEM16D
A_23_P422831	0.06	0.01	0.39	0.05	NM_004816	C9orf61
A_32_P34552	0.11	0.01	0.37	0.03	NM_002690	POLB
A_23_P266	1.33	0.09	3.03	0.21	U82382	PIN1
A_23_P101992	0.01	0.01	0.33	0.03	NM_006770	MARCO
A_24_P863124	0.03	0.01	1.37	0.10		
A_23_P119562	6.66	0.47	28.67	2.65	NM_001928	CFD
A_24_P56388	0.79	0.07	3.69	0.39	NM_181054	HIF1A
A_23_P379327	0.13	0.01	0.65	0.05	AB032990	
A_23_P37624	0.06	0.01	0.23	0.02	AF140675	ADAMTS7
A_23_P77623	0.01	0.01	0.78	0.06	NM_006927	ST3GAL2
A_24_P918317	0.01	0.01	19.65	2.39	NM_015881	DKK3
A_24_P222397	0.01	0.01	0.14	0.02	NM_021179	C1orf114
A_32_P29118	0.08	0.01	3.16	0.22	NM_152754	SEMA3D
A_24_P375453	0.15	0.02	0.90	0.07	NM_001009955	SSBP3
A_23_P150018	0.28	0.02	1.22	0.10	NM_004419	DUSP5
A_32_P48279	0.01	0.01	2.32	0.29		
A_23_P352799	0.32	0.02	4.18	0.30	AB084276	NPW
A_24_P161018	0.16	0.02	0.69	0.07	NM_017554	PARP14
A_23_P65240	0.05	0.01	0.66	0.12	NM_001845	COL4A1
A_23_P401106	0.15	0.01	2.04	0.22	NM_002599	PDE2A
A_23_P256716	0.23	0.02	0.53	0.04	NM_003601	SMARCA5
A_24_P261567	0.27	0.02	0.96	0.07	NM_030792	GDPD5
A_24_P238257	0.01	0.01	0.40	0.06	AK093167	
A_23_P369966	0.78	0.07	2.85	0.26	NM_152426	APOBEC3D
A_23_P303718	0.47	0.03	1.60	0.14	NM_015548	DST
A_23_P133438	0.06	0.01	0.70	0.11	NM_019018	FAM105A
A_23_P51487	0.01	0.01	0.33	0.04	NM_018284	GBP3
A_23_P118660	0.99	0.07	2.71	0.19	NM_152300	DDX52
A_23_P67980	0.06	0.01	0.19	0.02	NM_003709	KLF7
A_24_P176805	0.06	0.01	0.31	0.03	NM_003417	ZNF264
A_23_P14915	1.49	0.12	4.89	0.35	NM_001896	CSNK2A2
A_24_P419250	1.16	0.11	4.61	0.43	NM_170692	RASAL2
A_23_P138881	0.10	0.01	0.65	0.07	NM_001104	ACTN3
A_32_P34444	0.04	0.01	0.50	0.07	NM_025135	FHOD3
A_24_P687594	0.03	0.01	0.48	0.04	CR627381	LIX1L

A_24_P297888	0.01	0.01	4.03	0.79	NM_002451	MTAP
A_23_P107763	0.45	0.03	1.71	0.12	NM_006631	ZNF266
A_23_P408285	0.05	0.01	0.39	0.04	NM_153026	PRICKLE1
A_23_P252062	0.12	0.01	1.88	0.25	NM_138711	PPARG
A_23_P201445	4.00	0.35	16.47	1.64	NM_016076	C1orf121
A_32_P157965	1.63	0.13	5.92	0.54	NM_003908	EIF2S2
A_23_P14105	0.06	0.01	0.24	0.02	NM_001268	RCBTB2
A_24_P307580	0.15	0.01	0.31	0.02	AF092095	HTATIP2
A_32_P116840	0.36	0.03	1.40	0.13	NM_203356	CTAGE5
A_24_P11462	0.32	0.02	1.16	0.09	NM_052998	ADC
A_23_P200015	0.01	0.01	0.41	0.06	NM_174858	AK5
A_24_P418637	0.09	0.01	0.43	0.05	NM_012090	MACF1
A_23_P48936	1.51	0.10	4.72	0.37	NM_005902	SMAD3
A_24_P402588	0.06	0.01	7.83	0.55	NM_138553	BCL11A
A_23_P103628	3.74	0.27	10.58	0.75	AK098212	HEATR1
A_24_P410363	0.29	0.04	2.50	0.37	NM_022051	EGLN1
A_32_P30004	0.18	0.01	1.53	0.15	AF086044	
A_24_P269624	0.03	0.01	0.15	0.01	BE090931	
A_23_P69179	0.08	0.01	12.62	1.41	NM_018192	LEPREL1
A_24_P401739	2.45	0.17	5.32	0.38	NM_001006634	ARHGAP17
A_23_P160377	0.36	0.03	3.02	0.28	NM_003462	DNALI1
A_23_P83134	0.02	0.01	0.28	0.02	NM_002048	GAS1
A_24_P28977	0.11	0.01	0.58	0.06	NM_003304	TRPC1
A_32_P146579	0.01	0.01	0.12	0.01	AK128756	
A_23_P389987	0.04	0.01	0.47	0.05	NM_016170	
A_32_P30345	0.09	0.01	0.25	0.02		
A_32_P149435	0.01	0.01	0.14	0.02	BX107899	
A_24_P29885	0.04	0.01	1.06	0.22	NM_002514	NOV
A_23_P29684	0.05	0.01	0.80	0.06	NM_015873	VILL
A_23_P53176	0.35	0.03	3.55	0.32	NM_016725	FOLR1
A_24_P336705	2.62	0.22	9.39	0.66	NM_004582	RABGGTB
A_32_P75299	20.12	1.42	62.60	4.79	NM_001001790	
A_23_P90172	0.72	0.05	2.60	0.22	NM_014330	PPP1R15A
A_23_P3681	3.19	0.25	13.60	1.32	NM_018092	NETO2
A_24_P304439	1.47	0.10	5.08	0.38	NM_006843	SDS
A_32_P3527	0.25	0.02	0.82	0.07	AL711212	
A_23_P66347	10.57	0.75	44.16	3.52		
A_23_P72050	0.50	0.04	2.40	0.25	NM_153831	PTK2
A_23_P209636	0.09	0.01	1.07	0.11	NM_006449	CDC42EP3
A_23_P318300	0.38	0.03	3.51	0.34	NM_133646	
A_24_P273143	3.59	0.25	18.02	1.61	NM_052871	
A_32_P11723	24.60	1.86	76.10	5.76	CR595813	EIF2S2
A_23_P205531	0.20	0.02	1.30	0.17	NM_194430	RNASE4
A_23_P38106	8.11	0.57	39.98	3.63	NM_021972	SPHK1
A_24_P186379	3.35	0.24	12.18	1.06	NM_198472	C10orf125
A_23_P335661	0.02	0.01	1.22	0.10	AB028976	SAMD4A
A_23_P131255	2.06	0.16	12.12	1.36	NM_015535	
A_23_P216610	0.15	0.02	0.93	0.07	NM_022486	SUSD1
A_23_P400580	0.06	0.01	0.36	0.03	AB040883	
A_24_P216654	0.22	0.02	1.15	0.12	NM_003101	SOAT1
A_23_P58676	0.19	0.02	1.74	0.22	NM_024563	C5orf23
A_23_P364504	1.69	0.12	15.69	1.90	AK094353	
A_23_P213014	0.01	0.01	0.21	0.03	NM_001001290	SLC2A9
A_23_P393620	0.02	0.01	44.91	6.89	NM_006528	TFPI2
A_24_P263653	0.01	0.01	0.42	0.04	NM_004946	DOCK2
A_24_P59607	0.09	0.02	2.37	0.27	BC019938	C17orf51
A_23_P22557	0.63	0.04	1.65	0.12	AK022463	
A_23_P39465	11.01	0.84	40.13	3.58	NM_004335	BST2
A_23_P98686	1.10	0.08	3.04	0.21	NM_025092	ATHL1
A_23_P67224	0.05	0.01	0.73	0.05	NM_003811	TNFSF9
A_23_P98995	0.47	0.03	1.18	0.08	NM_020898	CALCOCO1
A_24_P16214	0.70	0.05	1.95	0.14	AK090827	
A_24_P354496	0.06	0.01	0.34	0.04	NM_153008	
A_32_P117812	0.03	0.01	0.12	0.01	BX640928	LYSMD4
A_23_P31866	1.06	0.08	2.46	0.17	NM_080651	THRAP6
A_23_P146637	0.02	0.01	27.04	1.91	NM_005866	OPRS1

A_23_P372096	0.18	0.02	1.55	0.13	AK097804	C20orf112
A_23_P203013	0.07	0.01	0.19	0.02	NM_002519	NPAT
A_23_P24457	0.01	0.01	0.08	0.01	NM_020929	LRRC4C
A_24_P408424	8.90	0.63	27.14	2.20	NM_002473	MYH9
A_23_P121265	6.59	0.53	20.58	1.46	NM_007284	TWF2
A_24_P142343	0.01	0.01	0.32	0.02	XM_290734	
A_23_P24077	0.11	0.01	1.58	0.11	NM_022153	C10orf54
A_23_P324327	0.24	0.02	3.00	0.24	NM_016235	GPRC5B
A_23_P53891	0.38	0.03	1.40	0.13	NM_001730	KLF5
A_23_P147805	0.17	0.01	2.25	0.16	BC047030	UPP1
A_23_P47304	0.05	0.01	13.31	0.94	NM_004347	CASP5
A_23_P383258	0.01	0.01	1.26	0.13	NM_004293	GDA
A_24_P371303	0.97	0.07	2.60	0.18	NM_015224	C3orf63
A_32_P104572	0.11	0.01	0.22	0.02	BM670971	NUDC
A_24_P377499	0.01	0.01	0.72	0.14	NM_145323	OSBPL3
A_24_P942481	0.25	0.02	0.93	0.07	NM_180989	GPR180
A_23_P73114	0.04	0.01	0.88	0.06	NM_000313	PROS1
A_23_P3242	1.02	0.08	3.17	0.26	BC064969	
A_24_P310756	0.20	0.02	0.69	0.05	AB007952	FBXO28
A_23_P252163	0.01	0.01	0.53	0.05	NM_004938	DAPK1
A_23_P67339	0.16	0.01	0.55	0.04	NM_020650	RCN3
A_23_P168551	0.10	0.01	0.45	0.03	NM_153247	SLC29A4
A_23_P23194	4.72	0.33	12.69	0.90	NM_032409	PINK1
A_23_P107283	0.02	0.01	5.93	0.80	NM_002145	HOXB2
A_32_P99171	0.13	0.02	2.11	0.19	AF131762	
A_23_P52058	0.58	0.05	2.35	0.24	NM_005646	TARBP1
A_23_P9416	3.72	0.26	16.42	1.55	NM_002197	ACO1
A_23_P259561	0.04	0.01	0.95	0.07	CD690854	
A_23_P307844	0.01	0.01	1.44	0.10	NM_174933	PHYHD1
A_23_P111311	0.01	0.01	1.58	0.31	NM_144497	AKAP12
A_24_P52921	0.01	0.01	0.78	0.07	NM_005504	BCAT1
A_24_P417267	0.01	0.01	0.06	0.01	AK097861	
A_24_P171075	1.72	0.14	5.90	0.53	NM_183013	CREM
A_24_P328504	0.14	0.01	0.32	0.02	NM_007237	SP140
A_24_P364296	0.18	0.02	1.16	0.14	NM_001980	STX2
A_32_P62796	0.01	0.01	0.06	0.01	CR740121	
A_23_P250825	1.29	0.11	4.78	0.46	NM_014504	RABGEF1
A_23_P211522	4.66	0.33	13.38	1.01	NM_145738	SYNGR1
A_23_P162171	0.34	0.06	20.15	1.55	NM_006500	MCAM
A_23_P501276	26.77	1.89	94.47	8.24	NM_001069	TUBB2A
A_23_P425332	0.01	0.01	0.11	0.01	NM_058237	KIAA1622
A_24_P923757	0.07	0.01	0.19	0.02	NM_018179	ATF7IP
A_32_P202214	0.06	0.01	0.22	0.02	CR627188	LY6K
A_24_P48204	0.65	0.05	1.34	0.10	NM_003004	SECTM1
A_24_P898945	0.07	0.01	0.31	0.03	U78519	
A_32_P167825	0.02	0.01	0.45	0.04		
A_23_P146347	0.19	0.02	0.81	0.08	NM_017645	FAM29A
A_24_P95439	0.61	0.04	2.31	0.16	NM_001014438	CARS
A_23_P257945	5.68	0.45	19.47	1.76	NM_005333	HCCS
A_23_P205959	0.05	0.01	5.40	0.45	NM_000693	ALDH1A3
A_23_P29330	0.01	0.01	1.17	0.19	NM_148674	SMC1B
A_23_P55477	1.19	0.08	8.05	0.57	NM_000676	ADORA2B
A_23_P402157	0.45	0.03	1.86	0.13	NM_024681	KCTD17
A_23_P206169	0.14	0.02	0.66	0.07	NM_173500	TTBK2
A_23_P256384	0.39	0.05	1.94	0.22	NM_021144	PSIP1
A_23_P47377	1.53	0.14	5.20	0.38	NM_016142	HSD17B12
A_24_P256404	0.08	0.01	0.76	0.05	AK093202	
A_32_P171427	0.01	0.01	0.07	0.01		
A_23_P88347	0.19	0.03	1.86	0.16	NM_006832	PLEKHC1
A_23_P216149	1.06	0.08	2.82	0.20	NM_017489	TERF1
A_23_P60579	7.21	0.51	19.28	1.38	NM_002435	MPI
A_23_P64879	0.67	0.05	1.92	0.14	NM_004982	KCNJ8
A_23_P91640	0.12	0.01	0.63	0.06	NM_020437	ASPHD2
A_23_P168531	0.12	0.01	0.55	0.04	NM_003088	FSCN1
A_23_P212497	0.07	0.01	0.60	0.09	NM_032169	ACAD11
A_24_P784765	0.04	0.01	0.75	0.11	NM_203330	CD59

A_23_P53467	0.51	0.04	4.99	0.67	NM_201612	
A_24_P194017	6.83	0.48	15.89	1.12	NM_015953	NOSIP
A_23_P214908	1.16	0.08	4.22	0.33	AY374131	MTHFD1L
A_23_P213766	0.16	0.02	0.70	0.05	NM_054027	ANKH
A_23_P256676	0.60	0.05	4.04	0.50	NM_013396	USP25
A_24_P355246	0.01	0.01	0.28	0.04	AK023096	
A_32_P25050	0.15	0.01	0.93	0.08	NM_172037	RDH10
A_32_P115947	1.26	0.11	4.27	0.36	BC040651	IDI2
A_24_P331918	0.01	0.01	0.09	0.01	NM_001848	COL6A1
A_24_P295245	0.23	0.02	5.33	0.77	NM_032467	ASPH
A_24_P264166	0.01	0.01	0.11	0.01	NM_001012452	
A_32_P174790	0.01	0.01	0.47	0.03	BC048265	
A_23_P203983	0.95	0.07	3.10	0.22	NM_019034	RHOF
A_23_P34597	0.03	0.01	4.31	0.31	NM_001785	CDA
A_23_P348146	0.14	0.01	0.42	0.03	NM_144595	SLAIN1
A_23_P166248	0.24	0.02	0.77	0.06	NM_004414	DSCR1
A_24_P32085	0.01	0.01	0.73	0.08	NM_024761	MOBKL2B
A_23_P342910	0.22	0.02	0.55	0.04	AB051464	KLHL15
A_23_P164316	0.59	0.04	1.46	0.10	BC044941	
A_24_P134340	1.39	0.11	4.85	0.34	NM_182483	NSFL1C
A_32_P234459	7.85	0.55	41.61	3.76	NR_001434	HLA-H
A_32_P192314	0.08	0.01	1.06	0.10		
A_23_P143102	0.05	0.01	1.40	0.10	BC004960	
A_23_P125383	0.01	0.01	0.18	0.01	NM_016192	TMEFF2
A_23_P94422	2.14	0.17	6.46	0.46	NM_014791	MELK
A_23_P87532	0.18	0.02	0.61	0.04	NM_001002259	CAPRIN2
A_23_P96568	2.89	0.21	8.12	0.57	NM_001456	FLNA
A_23_P77993	0.13	0.01	1.19	0.08	NM_006688	C1QL1
A_24_P133171	1.06	0.08	10.31	1.34	AK026078	
A_23_P48637	0.47	0.05	2.16	0.24	BG108194	HIF1A
A_23_P55149	1.72	0.13	5.49	0.39	NM_001282	AP2B1
A_24_P339974	1.13	0.08	3.10	0.22	NM_012305	AP2A2
A_24_P169634	0.01	0.01	0.05	0.01	AF019382	
A_23_P145644	0.01	0.01	0.09	0.01	NM_000790	DDC
A_32_P133090	0.10	0.01	1.39	0.12	XM_379210	
A_23_P102462	0.03	0.01	1.91	0.22	NM_153214	
A_23_P77529	0.06	0.01	3.73	0.26	NM_005823	MSLN
A_23_P85140	0.12	0.03	5.92	0.71	NM_080390	TCEAL2
A_23_P214026	0.01	0.01	6.83	0.89	NM_001999	FBN2
A_23_P418031	1.86	0.13	4.89	0.35	AK024480	
A_24_P208721	0.07	0.01	0.17	0.02	NM_025010	KLHL18
A_23_P145006	0.70	0.05	2.53	0.18	NM_054023	SCGB3A2
A_23_P101093	0.40	0.03	2.09	0.18	NM_016429	COPZ2
A_24_P220485	0.37	0.03	2.29	0.24	NM_182487	OLFML2A
A_24_P184799	0.09	0.01	6.12	0.43	NM_004086	COCH
A_24_P152968	0.08	0.01	0.25	0.02	NM_001353	AKR1C1
A_23_P16671	0.82	0.06	2.46	0.17	NM_024050	C19orf58
A_24_P7594	0.01	0.01	0.12	0.01	AK097266	
A_24_P65910	3.13	0.22	11.22	1.00	NM_018244	C20orf44
A_23_P31810	4.81	0.34	32.67	3.34	NM_005195	CEBPD
A_32_P157208	0.03	0.01	0.33	0.03	AY343891	
A_23_P117980	1.63	0.12	4.43	0.31	NM_005886	KATNB1
A_23_P149050	0.08	0.01	0.32	0.02	NM_024796	
A_23_P370682	0.06	0.01	1.71	0.12	NM_138456	BATF2
A_23_P347632	0.13	0.01	0.41	0.03	NM_014751	MTSS1
A_24_P287664	0.02	0.01	0.31	0.04	NM_004573	PLCB2
A_23_P103371	0.10	0.01	0.37	0.03	NM_052998	ADC
A_24_P307025	3.88	0.27	10.96	0.80	NR_000029	RPL23AP7
A_24_P69654	0.96	0.07	12.81	1.57	NM_001300	
A_23_P413585	0.09	0.01	0.25	0.02	NM_004474	FOXD2
A_23_P408271	0.38	0.03	3.60	0.50	NM_016245	HSD17B11
A_24_P630109	0.04	0.01	0.15	0.01	AF007192	
A_24_P333293	0.04	0.01	0.12	0.01	NM_016575	NT5DC3
A_23_P211816	1.36	0.10	3.33	0.24	NM_002375	MAP4
A_23_P145507	0.01	0.01	0.42	0.03	NM_004100	EYA4
A_23_P414713	0.01	0.01	0.08	0.01	NM_032423	ZNF528

A_23_P76749	1.10	0.10	5.23	0.52	AB032956	GALNTL1
A_23_P158096	1.59	0.12	8.88	0.91	AK021957	COL27A1
A_32_P192823	0.51	0.04	2.77	0.20	NM_175886	PRPS1L1
A_23_P61466	0.03	0.01	0.52	0.04	NM_174941	CD163L1
A_24_P149036	0.09	0.01	8.21	0.58	NM_001387	DPYSL3
A_23_P88678	0.18	0.02	1.31	0.16	NM_152335	C15orf27
A_32_P232559	0.01	0.01	3.84	0.30	AY007155	
A_24_P97825	0.02	0.01	0.23	0.02	NM_015621	CCDC69
A_24_P391918	0.01	0.01	0.07	0.01	NM_173464	L3MBTL4
A_23_P125107	6.52	0.46	53.09	4.58	NM_005514	HLA-B
A_23_P43425	1.44	0.10	6.99	0.65	NM_017998	C9orf40
A_23_P418204	0.07	0.01	0.16	0.01	BC006258	
A_24_P55092	0.01	0.01	0.43	0.04	NM_133493	CD109
A_23_P123234	0.02	0.01	17.28	1.87		
A_32_P80678	0.05	0.01	0.47	0.04	NM_001005845	ADAM9
A_32_P62026	0.01	0.01	0.07	0.01		
A_23_P164733	0.15	0.01	0.32	0.02	NM_004756	NUMBL
A_23_P328323	0.13	0.01	0.44	0.03	NM_018211	RAVER2
A_32_P155247	36.33	2.57	104.22	7.37	NM_000146	FTL
A_32_P93584	0.03	0.01	2.14	0.15		
A_23_P118722	0.37	0.03	8.05	0.94	NM_001671	ASGR1
A_23_P95764	7.14	0.51	30.14	2.63	NM_002764	PRPS1
A_23_P384085	0.19	0.02	0.80	0.06	NM_181453	GCC2
A_23_P503233	37.67	2.66	80.33	5.68	NM_080738	EDARADD
A_23_P203419	5.66	0.40	26.98	2.58	NM_013402	FADS1
A_23_P18684	0.08	0.01	3.03	0.41	NM_004362	CLGN
A_23_P256205	0.73	0.06	2.92	0.26	NM_014945	ABLIM3
A_24_P260361	0.15	0.01	0.63	0.05	NM_176824	BBS7
A_23_P29836	6.17	0.46	20.24	1.51	NM_144638	TMEM42
A_24_P526190	0.03	0.01	0.54	0.06	BC034407	
A_23_P44724	3.70	0.26	18.94	1.77	NM_001321	CSRP2
A_23_P113994	0.03	0.01	0.45	0.03	NM_018240	KIRREL
A_32_P138950	0.01	0.01	0.14	0.01	BC034326	
A_23_P140884	2.28	0.16	8.69	0.72	BC014971	
A_23_P127891	0.01	0.01	3.92	0.73	NM_170735	BDNF
A_24_P143189	12.80	0.91	106.71	11.30	NM_183049	TMSL3
A_23_P389897	0.05	0.01	0.24	0.02	NM_002507	NGFR
A_32_P114246	0.28	0.02	0.92	0.07	AL832991	USP47
A_23_P41204	0.28	0.02	1.24	0.11	NM_144635	FAM131A
A_23_P215900	1.84	0.13	4.15	0.29	NM_016240	SCARA3
A_32_P140489	0.01	0.01	0.07	0.01	NM_001001557	GDF6
A_32_P74120	17.57	1.32	58.37	4.13	BC070363	
A_32_P183904	0.47	0.05	4.08	0.29	BC007586	SHF
A_23_P123336	0.44	0.03	3.69	0.34	NM_018444	PPM2C
A_24_P304154	0.01	0.01	0.10	0.01	NM_000480	AMPD3
A_23_P330070	0.09	0.01	2.01	0.14	BC015514	TFPI
A_32_P46238	0.04	0.01	0.36	0.05	NR_001443	
A_23_P34710	1.36	0.10	7.11	0.62	CR604521	
A_23_P168651	0.01	0.01	0.24	0.03	NM_001259	CDK6
A_23_P113453	0.01	0.01	0.12	0.01		
A_24_P410952	1.06	0.07	2.69	0.19	NM_003768	PEA15
A_32_P163306	0.05	0.01	1.26	0.10	AB058725	KIAA1822
A_32_P104263	1.79	0.13	14.80	1.05		
A_24_P478556	0.12	0.01	0.68	0.05	BC030102	
A_23_P142560	0.01	0.01	0.49	0.07	NM_014795	ZEB2
A_23_P76538	2.71	0.19	22.54	1.97	NM_017899	TESC
A_23_P142631	0.67	0.05	2.31	0.16	NM_054033	FKBP1B
A_23_P358244	0.09	0.01	0.21	0.02	D90278	CEACAM3
A_23_P131183	0.05	0.01	0.99	0.17	NM_001485	GBX2
A_23_P148609	0.19	0.02	1.83	0.13	NM_021796	PLAC1
A_32_P126733	0.01	0.01	0.06	0.01		
A_24_P218688	0.30	0.02	0.80	0.06	NM_000694	ALDH3B1
A_23_P6822	0.01	0.01	0.10	0.01	NM_002217	ITIH3
A_23_P91250	0.01	0.01	0.08	0.01	NM_199441	ZNF334
A_23_P36831	0.09	0.01	0.53	0.06	NM_003979	GPRC5A
A_23_P170839	2.44	0.17	6.43	0.45	NM_001008491	2.Sep

A_23_P5241	0.31	0.02	0.62	0.04	NM_213604	ADAMTSL5
A_32_P132827	0.43	0.03	1.76	0.14		
A_32_P106376	0.01	0.01	0.11	0.01	NM_182645	VGLL2
A_23_P121037	0.16	0.01	0.58	0.04	NM_015720	PODXL2
A_24_P316364	3.02	0.31	18.53	2.08	NM_015523	REXO2
A_24_P535256	0.01	0.01	0.40	0.03	AK001903	
A_23_P65851	0.07	0.01	0.21	0.02	AK024793	
A_23_P425502	0.47	0.03	1.31	0.09	NM_017613	DONSON
A_23_P51397	2.20	0.19	8.91	0.64	NM_001008493	ENAH
A_23_P147711	0.03	0.01	0.30	0.04	NM_000906	NPR1
A_32_P23047	0.09	0.01	0.73	0.05		
A_32_P351968	0.05	0.01	1.65	0.13	NM_002118	HLA-DMB
A_24_P887857	0.13	0.01	0.51	0.04	XM_496202	
A_23_P50146	0.08	0.01	0.58	0.06	NM_213602	SIGLEC15
A_24_P677890	0.56	0.04	1.63	0.12	BC016384	
A_23_P167276	0.69	0.07	3.09	0.26	NM_177453	
A_23_P394395	0.20	0.02	0.84	0.06	NM_020433	JPH2
A_32_P232523	0.04	0.01	0.28	0.02		
A_24_P105191	0.99	0.09	4.07	0.36	NM_147175	HS6ST2
A_32_P194704	0.02	0.01	0.06	0.01	BI836739	
A_32_P77989	0.97	0.09	5.25	0.56	NM_018092	NETO2
A_23_P138680	0.20	0.02	0.95	0.08	NM_172200	IL15RA
A_23_P250156	0.06	0.01	5.03	0.36	NM_006548	IGF2BP2
A_23_P357571	0.01	0.01	0.79	0.06	NM_000854	GSTT2
A_23_P310956	0.12	0.02	7.09	0.50	NM_058175	COL6A2
A_23_P70818	0.04	0.01	7.62	0.61	NM_005631	SMO
A_23_P501781	0.01	0.01	0.07	0.01	NM_153767	KCNJ1
A_23_P22072	0.03	0.01	0.20	0.02	NM_006021	
A_24_P261417	0.01	0.01	11.32	1.12	NM_015881	DKK3
A_23_P121545	0.01	0.01	0.18	0.02	NM_201591	GPM6A
A_23_P337689	0.01	0.01	0.24	0.03	NM_015855	WIT1
A_23_P91346	0.40	0.03	1.46	0.13	BC008667	
A_23_P57709	0.87	0.08	3.59	0.34	NM_013363	PCOLCE2
A_23_P85004	0.09	0.01	0.44	0.05	NM_007309	DIAPH2
A_23_P216307	0.01	0.01	0.29	0.04	NM_004349	RUNX1T1
A_32_P16451	0.12	0.01	0.85	0.11	BE855644	
A_24_P196519	0.01	0.01	0.43	0.05	NM_002451	MTAP
A_23_P137786	0.11	0.01	0.62	0.04	AK023606	ADAMTSL4
A_23_P122439	0.41	0.03	1.39	0.11	NM_181531	BTN2A2
A_32_P80455	0.02	0.01	0.53	0.04	BX647070	RORB
A_23_P24884	0.25	0.02	1.07	0.09	NM_005418	ST5
A_24_P4816	0.08	0.01	3.54	0.39	NM_031412	GABARAPL1
A_23_P164814	1.06	0.08	3.74	0.31	NM_024323	C19orf57
A_32_P115606	0.43	0.04	2.01	0.18	AK131385	PXK
A_23_P166087	1.23	0.11	5.47	0.54	NM_014737	RASSF2
A_32_P313405	0.01	0.01	8.33	0.78	NM_005559	LAMA1
A_23_P169039	0.01	0.01	0.72	0.05	NM_003068	SNAI2
A_24_P384029	0.02	0.01	0.10	0.01		
A_23_P255104	0.80	0.07	3.44	0.24	NM_005779	LHFPL2
A_23_P138194	0.01	0.01	0.62	0.07	NM_000433	NCF2
A_23_P72697	0.07	0.01	0.63	0.05	NM_178172	
A_24_P273157	0.16	0.02	0.71	0.06	NM_052843	OBSCN
A_32_P462013	0.65	0.06	3.50	0.39	AK021694	
A_24_P391586	0.43	0.05	2.39	0.20	NM_178507	OAF
A_23_P144959	0.02	0.01	0.80	0.10	NM_004385	VCAN
A_23_P218637	1.09	0.10	4.22	0.32	NM_005054	RGPD5
A_32_P176018	0.52	0.05	2.23	0.16	NM_030812	ACTL8
A_23_P29124	0.51	0.04	2.80	0.24	NM_000407	GP1BB
A_23_P17914	0.26	0.02	3.03	0.42	NM_025225	PNPLA3
A_32_P85433	0.01	0.01	0.17	0.02	BQ130701	
A_32_P231265	0.22	0.02	0.61	0.04	AI694800	
A_23_P20864	0.04	0.01	1.23	0.15	NM_012098	ANGPTL2
A_24_P379512	0.23	0.02	0.83	0.06	CR594196	
A_23_P110686	3.09	0.22	37.01	3.18	NM_003714	STC2
A_24_P756657	1.90	0.13	8.80	0.73	AL049461	
A_23_P16262	0.07	0.01	0.41	0.03	NM_004533	MYBPC2

A_24_P255524	0.01	0.01	0.06	0.01	AF247820	CALD1
A_24_P373152	0.20	0.02	3.31	0.41	NM_021914	CFL2
A_32_P94798	49.10	3.47	433.31	46.56	NM_001002857	ANXA2
A_23_P45304	0.25	0.02	1.14	0.11	NM_021083	XK
A_32_P29703	0.24	0.02	16.77	1.59	BC042853	
A_23_P91230	0.22	0.02	2.99	0.21	NM_003064	SLPI
A_23_P46429	2.02	0.15	186.13	30.77	NM_001554	CYR61
A_24_P314179	0.27	0.03	1.41	0.11	NM_005239	ETS2
A_23_P13725	0.01	0.01	0.98	0.17	NM_182767	SLC6A15
A_24_P852756	0.01	0.01	0.05	0.01	NM_020056	HLA-DQA2
A_23_P156788	0.03	0.01	0.42	0.05	NM_003764	STX11
A_32_P60145	0.02	0.01	0.07	0.01	AL834308	C1orf167
A_23_P69868	8.32	0.59	24.10	1.73	NM_022978	SERF1B
A_24_P511686	0.42	0.05	2.64	0.31	CR616845	
A_32_P452655	0.11	0.01	0.51	0.04	NM_009587	LGALS9
A_23_P207319	1.03	0.07	4.04	0.31	NM_003954	MAP3K14
A_23_P7325	0.07	0.01	2.10	0.30	NM_004334	BST1
A_23_P425681	0.01	0.01	0.26	0.04	NM_000729	CCK
A_23_P118061	4.12	0.37	17.09	1.57	NM_181641	CKLF
A_23_P138805	0.18	0.02	1.90	0.29	NM_012124	CHORDC1
A_24_P348090	0.18	0.02	0.87	0.09	NM_002396	ME2
A_23_P155837	0.08	0.01	0.20	0.02	NM_183075	CYP2U1
A_24_P235266	0.42	0.04	3.06	0.36	NM_001001555	GRB10
A_23_P149075	5.72	0.40	23.83	1.69	NM_018067	MAP7D1
A_23_P313828	0.33	0.02	1.49	0.12	NM_181716	PRR6
A_23_P423348	0.02	0.01	0.16	0.02	NM_139017	IL31RA
A_23_P51958	0.26	0.02	0.65	0.05	NM_182744	NBL1
A_23_P209904	3.45	0.24	16.81	1.54	NM_002081	GPC1
A_32_P224727	0.58	0.06	2.65	0.25		
A_23_P256581	0.01	0.01	7.20	1.01	NM_021620	PRDM13
A_23_P50825	0.26	0.03	1.82	0.22	D70836	ZNF714
A_23_P54447	0.02	0.01	0.10	0.01	NM_030944	
A_23_P48056	1.79	0.20	13.29	1.01	NM_006825	CKAP4
A_23_P81298	0.90	0.07	6.85	0.78	AK025816	
A_32_P230868	9.17	0.65	19.89	1.41	NM_024519	FAM65A
A_23_P96965	0.11	0.01	1.01	0.13	NM_030786	SYNC1
A_23_P35597	0.06	0.01	0.35	0.03	NM_007021	C10orf10
A_24_P820372	0.10	0.01	0.69	0.07		
A_23_P27758	0.01	0.01	0.16	0.02	AK092138	
A_23_P41765	0.71	0.05	2.38	0.17	NM_002198	IRF1
A_32_P123729	0.18	0.01	0.88	0.06	BX394228	
A_23_P18579	20.35	1.44	48.63	3.44	NM_006607	PTTG2
A_32_P175935	0.03	0.01	0.66	0.10	AK125162	
A_23_P326325	0.03	0.01	0.13	0.01	AK131446	C6orf170
A_23_P5731	3.23	0.27	11.80	0.95	NM_016044	FAHD2A
A_24_P936145	0.02	0.01	0.67	0.05	BX647214	GNAO1
A_23_P111525	0.86	0.06	3.69	0.35	BX648200	
A_24_P339611	6.45	0.46	18.31	1.29	NM_004708	PDCD5
A_23_P75088	0.03	0.01	0.16	0.01	NM_024895	PDZD7
A_23_P357724	0.01	0.01	0.19	0.01	NM_002246	KCNK3
A_23_P202071	0.01	0.01	1.11	0.14	NM_006561	CUGBP2
A_23_P77459	3.89	0.35	16.24	1.51	NM_003905	APPBP1
A_24_P527716	0.01	0.01	0.12	0.02	BC094740	
A_32_P205944	0.76	0.05	4.61	0.46	NM_005054	RGPD5
A_32_P124773	0.18	0.02	0.61	0.05		
A_24_P236008	0.85	0.06	1.84	0.13	AK001597	SCYL2
A_23_P319640	0.47	0.03	1.02	0.07	BC009264	
A_23_P40847	0.01	0.01	0.53	0.05	NM_004267	CHST2
A_23_P33673	0.05	0.01	0.26	0.02	NM_001003818	TRIM6
A_24_P180654	0.30	0.04	2.18	0.23	BC046181	CREB3L2
A_23_P206396	7.51	0.64	29.78	2.11	NM_016951	CKLF
A_23_P100344	2.24	0.16	7.11	0.54	NM_014321	ORC6L
A_23_P48109	0.01	0.01	0.17	0.02	NM_016533	NINJ2
A_23_P201538	3.37	0.54	51.43	5.42	NM_002228	JUN
A_23_P41804	2.98	0.21	8.93	0.65	NM_033120	NKD2
A_23_P128036	2.86	0.20	12.22	1.12	NM_004990	MARS

A_32_P204795	0.01	0.01	0.22	0.02	BC008651	
A_23_P503072	0.01	0.01	0.23	0.02	NM_148672	
A_23_P427122	0.07	0.01	0.41	0.03	NM_053017	ART5
A_23_P211233	0.22	0.02	4.21	0.30	NM_001849	COL6A2
A_32_P112623	0.01	0.01	1.63	0.18	NM_175923	
A_23_P136721	0.04	0.01	0.60	0.04	U88896	
A_23_P376036	0.01	0.01	0.06	0.01	NM_152596	EXDL1
A_23_P358597	0.04	0.01	0.91	0.08	NM_022361	POPDC3
A_23_P161194	1.03	0.08	215.63	25.32	NM_003380	VIM
A_32_P79492	0.01	0.01	0.20	0.02	AK092432	PSCA
A_23_P25964	0.78	0.06	28.63	2.71	NM_000153	GALC
A_24_P230869	0.01	0.01	0.32	0.03	NM_003619	PRSS12
A_23_P153256	0.01	0.01	0.19	0.02	NM_198542	ZNF773
A_23_P92899	0.04	0.01	1.36	0.10	NM_031908	C1QTNF2
A_32_P203184	0.01	0.01	0.25	0.02	CR606809	
A_23_P151059	0.16	0.01	1.65	0.12	NM_018088	FAM90A1
A_24_P314477	0.04	0.01	5.45	0.57	NM_178012	TUBB2B
A_24_P933319	0.45	0.05	18.18	1.71	AK002107	
A_23_P169249	0.20	0.02	1.08	0.08	NM_017585	SLC2A6
A_23_P88865	2.38	0.19	61.27	5.46	NM_144601	CMTM3
A_23_P156327	0.16	0.02	207.23	14.65	NM_000358	TGFBI
A_23_P145024	0.01	0.00	6.71	0.80	NM_000024	ADRB2
A_23_P105957	12.94	0.92	60.49	4.28	NM_001102	ACTN1
A_23_P45456	0.01	0.01	0.22	0.02	NM_001289	CLIC2
A_23_P500614	0.03	0.01	1.91	0.19	NM_001243	TNFRSF8
A_23_P14821	0.01	0.01	1.09	0.11	NM_000814	GABRB3
A_24_P186370	0.01	0.01	0.20	0.02	NM_002444	MSN
A_23_P90710	0.01	0.01	0.22	0.02	NM_017569	FAM48A
A_23_P23611	0.01	0.01	0.23	0.02	NM_001008219	AMY1C
A_23_P259741	0.01	0.01	1.51	0.16	NM_002971	SATB1
A_23_P250413	0.01	0.01	0.31	0.02	NM_022141	PARVG
A_24_P112747	0.01	0.01	0.21	0.02	NM_005653	TFCP2
A_24_P345451	0.01	0.01	1.43	0.16	NM_024843	CYBRD1
A_24_P206328	0.01	0.01	0.75	0.08	NM_005020	PDE1C
A_23_P351837	1.39	0.10	14.30	1.01	NM_173583	
A_24_P339429	0.01	0.01	0.64	0.06	AK024229	
A_24_P23400	0.89	0.06	3.59	0.25	NM_005629	SLC6A8
A_24_P548953	0.01	0.01	0.40	0.04	NM_001013670	
A_32_P35595	0.15	0.01	1.00	0.07		
A_23_P163087	0.01	0.01	3.46	0.42	NM_007361	NID2
A_24_P409971	0.01	0.01	1.51	0.18	NM_144573	NEXN
A_24_P400027	0.01	0.01	0.29	0.02	NM_173698	FAM133A
A_23_P21976	0.08	0.01	7.00	0.50	NM_001897	CSPG4
A_23_P420293	0.01	0.01	0.31	0.03	NM_145013	C11orf45
A_32_P196142	0.06	0.01	1.10	0.08		
A_32_P154321	0.07	0.01	0.53	0.04	AW804939	LGALS3BP
A_23_P361014	0.05	0.01	2.76	0.29	NM_020856	TSHZ3
A_23_P42331	16.67	1.18	140.43	9.93	NM_145904	HMGA1
A_24_P639665	0.01	0.01	0.32	0.03	AK094342	
A_23_P14216	0.01	0.01	0.13	0.01		
A_24_P276808	0.01	0.01	0.22	0.02	NM_080589	PTPN7
A_23_P134041	0.01	0.01	1.58	0.15	BC040697	TBX18
A_23_P87752	0.02	0.01	14.21	1.39	NM_014262	LEPREL2
A_23_P211680	0.01	0.01	1.07	0.08	NM_015166	MLC1
A_24_P683013	0.01	0.01	0.79	0.08	BM696546	
A_23_P17130	0.01	0.01	0.09	0.01	NM_032321	
A_23_P132619	0.36	0.06	95.31	11.31	NM_000916	OXTR
A_23_P106922	0.08	0.01	1.30	0.09	NM_021615	CHST6
A_23_P64173	0.01	0.01	0.25	0.02	NM_001017534	
A_23_P433016	0.02	0.01	2.51	0.18	NM_001996	FBLN1
A_32_P200970	0.01	0.01	1.53	0.11	AI127867	CNN3
A_24_P272073	0.49	0.04	1.63	0.12		CYCSP52
A_23_P47616	0.01	0.01	0.82	0.09	NM_004476	FOLH1
A_23_P31064	0.01	0.01	3.66	0.43	NM_015529	MOXD1
A_23_P164958	0.04	0.01	2.61	0.24	NM_032040	CCDC8
A_23_P82503	0.02	0.01	2.08	0.16	AB049150	

A_24_P68153	0.18	0.01	0.96	0.07	BC005136	SLC35A3
A_32_P24832	0.01	0.01	0.89	0.09	NM_020190	OLFML3
A_23_P14863	1.75	0.12	4.90	0.35	NM_024111	CHAC1
A_23_P3111	0.28	0.02	7.98	0.69	NM_006821	ACOT2
A_23_P53588	0.08	0.01	8.59	0.91	NM_030775	WNT5B
A_32_P19840	0.20	0.02	9.04	0.64	XM_380151	
A_23_P345081	0.01	0.01	1.12	0.12	NM_001009956	
A_32_P216520	0.01	0.01	0.31	0.03	NM_007191	WIF1
A_24_P941322	0.02	0.01	0.15	0.01	NM_006775	QKI
A_23_P205713	0.24	0.02	9.77	0.92	NM_014178	STXBP6
A_23_P363149	0.01	0.01	0.17	0.01	AF034209	DKK3
A_24_P204244	6.27	0.44	98.07	6.95	NR_001562	ANXA2P1
A_24_P406986	0.01	0.01	2.64	0.29	NM_199329	SLC43A3
A_24_P108301	0.23	0.02	2.23	0.16	NM_173630	RTTN
A_23_P80594	0.01	0.01	0.58	0.05	NM_015184	PLCL2
A_24_P11315	0.01	0.01	0.67	0.06	NM_020190	OLFML3
A_32_P19101	0.15	0.01	3.23	0.23	BX097691	
A_23_P256487	0.02	0.01	0.73	0.05		
A_23_P1374	0.01	0.01	1.08	0.11	NM_006257	PRKCQ
A_32_P164573	0.01	0.01	0.30	0.02		
A_23_P14157	0.01	0.01	3.85	0.41	NM_198968	DZIP1
A_23_P26697	0.33	0.02	6.21	0.44	NM_033452	TRIM47
A_23_P340848	0.01	0.01	0.42	0.03	NM_000960	PTGIR
A_23_P142796	0.13	0.01	1.89	0.13	NM_017980	LIMS2
A_23_P360240	0.45	0.03	8.14	0.58	NM_138768	MYEOV
A_23_P332268	0.01	0.01	0.74	0.07	NM_033223	GABRG3
A_32_P152437	0.15	0.01	15.63	1.51	BC033829	
A_23_P209232	0.01	0.01	2.28	0.26	NM_024692	CLIP4
A_24_P400172	0.01	0.01	0.16	0.01	NM_182585	
A_24_P161036	0.36	0.03	9.29	0.72	NM_006821	ACOT2
A_24_P337104	0.08	0.01	9.75	1.16	NM_000916	OXTR
A_23_P18649	0.01	0.01	0.28	0.02	AK026709	FAT4
A_23_P303833	0.06	0.01	0.76	0.05	NM_174934	SCN4B
A_32_P7316	0.01	0.01	0.27	0.02	NM_170735	BDNF
A_23_P31006	1.10	0.08	8.71	0.62	NM_002125	HLA-DRB5
A_32_P61708	0.01	0.01	0.35	0.03		
A_23_P54840	36.03	2.55	77.81	5.50	NM_005946	MT1A
A_23_P64121	0.01	0.01	0.10	0.01	NM_152314	C11orf69
A_23_P106405	0.01	0.01	7.52	0.66	NM_002487	NDN
A_23_P315571	0.16	0.02	10.04	1.14	NM_015150	RFTN1
A_23_P99141	0.11	0.01	2.23	0.16	NM_019858	GPR162
A_23_P50217	0.01	0.01	1.27	0.13	NM_024833	ZNF671
A_23_P254626	0.01	0.01	5.36	0.85	NM_003919	SGCE
A_24_P349039	0.01	0.01	0.07	0.01	NM_020754	
A_24_P159515	0.08	0.01	0.18	0.02	NM_014726	TBKBP1
A_32_P103955	0.01	0.01	3.52	0.40		
A_32_P441530	0.01	0.01	0.59	0.04		
A_23_P374082	0.10	0.01	2.68	0.23	NM_033274	ADAM19
A_23_P207106	0.29	0.02	1.25	0.09	NM_000747	CHRNB1
A_24_P170726	0.01	0.01	0.09	0.01	AK094407	
A_24_P916547	0.10	0.01	7.93	0.84		
A_24_P203134	0.01	0.01	0.23	0.02	NM_178470	WDR40B
A_23_P94494	0.01	0.01	0.26	0.02	NM_007005	TLE4
A_23_P413888	0.01	0.01	0.20	0.02	BX640761	SLC35F1
A_23_P303087	0.01	0.01	1.79	0.20	NM_002825	PTN
A_23_P205057	0.01	0.01	1.68	0.19	NM_014459	
A_24_P256380	0.02	0.01	1.83	0.23	NM_024911	GPR177
A_23_P110052	0.01	0.01	3.86	0.42	NM_023067	FOXL2
A_23_P27265	0.01	0.01	0.18	0.01	NM_032160	DSEL
A_23_P58251	0.07	0.01	7.78	0.62	NM_001014448	CPZ
A_23_P76488	0.06	0.01	19.43	1.92	NM_001423	EMP1
A_24_P95070	0.01	0.01	2.26	0.26	AK129833	TFPI2
A_23_P51410	0.26	0.02	3.61	0.31	NM_022743	SMYD3
A_23_P15247	0.01	0.01	2.15	0.15	NM_013399	C16orf5
A_23_P38732	0.01	0.01	5.19	0.64	NM_001792	CDH2
A_23_P157628	0.07	0.01	0.23	0.02	NM_004942	DEFB4

A_24_P166443	0.05	0.01	2.47	0.29	NM_002121	HLA-DPB1
A_32_P205329	0.01	0.01	0.22	0.02	AF088007	
A_24_P913431	0.19	0.02	1.37	0.10	AK131423	TRIO
A_32_P134427	0.01	0.01	0.22	0.02	BC048201	
A_23_P218774	0.02	0.01	17.71	1.25	NM_002872	RAC2
A_23_P18078	0.01	0.01	0.13	0.01	NM_002888	RARRES1
A_23_P162640	0.19	0.02	8.23	0.94	NM_031412	GABARAPL1
A_23_P155057	0.01	0.01	0.20	0.02	NM_013385	PSCD4
A_23_P65401	1.15	0.08	12.30	0.94	NM_021914	CFL2
A_24_P418816	0.01	0.01	0.13	0.01	NM_015696	GPX7
A_24_P110780	0.01	0.01	0.23	0.02	NM_199343	
A_23_P17095	0.50	0.04	6.43	0.45	NM_006287	TFPI
A_23_P65789	0.01	0.01	0.09	0.01	AK002037	MCTP2
A_23_P160025	0.01	0.01	1.60	0.16	NM_005531	IFI16
A_24_P759477	0.01	0.01	0.30	0.03	BC042028	
A_23_P308483	0.01	0.01	0.21	0.02	NM_001001671	MAP3K15
A_23_P43273	0.56	0.05	14.34	1.49	NM_000127	EXT1
A_23_P109655	0.01	0.01	2.79	0.33	NM_025246	TMEM22
A_23_P83028	0.03	0.01	2.16	0.22	NM_021111	RECK
A_24_P458479	0.01	0.01	0.31	0.03	NM_001013404	
A_23_P35066	0.48	0.04	15.78	1.73	NM_015976	SNX7
A_23_P134109	0.04	0.01	5.50	0.92	NM_001431	EPB41L2
A_32_P114675	0.01	0.01	0.11	0.01		
A_23_P381714	0.01	0.01	0.70	0.07	NM_198584	CA13
A_23_P259292	0.09	0.01	1.31	0.09	NM_015645	C1QTNF5
A_24_P916496	0.27	0.02	17.21	2.10	NM_002737	PRKCA
A_32_P24376	0.01	0.01	3.94	0.42	BC063625	KRTAP2-4
A_23_P87700	0.01	0.01	2.28	0.16	NM_003480	MFAP5
A_23_P202978	0.01	0.01	2.07	0.23	NM_033292	CASP1
A_24_P365807	1.69	0.12	36.78	3.37	NM_004429	EFNB1
A_24_P922808	0.06	0.01	0.45	0.03	BC020640	C1orf121
A_23_P57784	0.09	0.01	2.19	0.20	NM_021101	CLDN1
A_24_P350589	0.06	0.01	0.43	0.03	NM_020724	RNF150
A_24_P322229	0.09	0.01	0.50	0.04	NM_033315	RASL10B
A_24_P925062	0.16	0.01	1.34	0.10	NM_001008528	MXRA7
A_23_P397285	0.01	0.01	2.57	0.32	NM_017527	LY6K
A_24_P272290	0.11	0.01	6.43	0.69	NM_183373	C6orf145
A_24_P184803	0.28	0.02	16.57	1.52	NM_004086	COCH
A_23_P124837	0.13	0.01	0.72	0.03	NM_002332	LRP1
A_24_P411186	0.01	0.01	0.67	0.07	NM_022893	BCL11A
A_23_P62607	0.01	0.01	0.33	0.03	NM_021258	IL22RA1
A_23_P141362	4.30	0.30	31.20	2.21	NM_001466	FZD2
A_24_P33982	0.01	0.01	0.09	0.01	BC053534	C17orf60
A_23_P64102	0.07	0.01	1.55	0.11	NM_004292	RIN1
A_24_P299685	0.01	0.01	2.96	0.21	NM_198389	PDPN
A_24_P115762	0.17	0.02	14.84	1.05	NM_148170	CTSC
A_23_P337242	0.01	0.01	0.10	0.01	NM_003242	TGFBR2
A_23_P421843	0.05	0.01	0.90	0.08	BC047081	SAMD14
A_23_P110276	0.01	0.01	3.56	0.29	BC004397	
A_24_P235429	0.01	0.01	0.08	0.01	NM_005502	ABCA1
A_32_P152586	0.01	0.01	0.97	0.10	BM970287	TITF1
A_23_P15542	0.87	0.07	12.25	1.06	NM_000413	HSD17B1
A_24_P400702	0.01	0.01	1.11	0.10		
A_24_P110141	0.02	0.01	0.64	0.07	NM_194295	
A_32_P3545	0.01	0.01	0.15	0.01	N52197	
A_23_P56746	0.01	0.01	1.91	0.23	NM_004460	FAP
A_23_P7528	0.01	0.01	0.43	0.04	NM_001387	DPYSL3
A_23_P128919	1.49	0.11	20.28	1.55	NM_002306	LGALS3
A_23_P330616	0.03	0.01	0.19	0.02	NM_003387	WIPF1
A_24_P5653	0.01	0.01	0.15	0.01	NM_138999	NETO1
A_24_P607880	0.01	0.01	3.26	0.39	AL832758	
A_23_P142974	0.03	0.01	0.19	0.02	NM_001007231	ARHGAP25
A_23_P210100	0.03	0.01	2.16	0.27	NM_019885	CYP26B1
A_23_P206707	2.41	0.17	7.06	0.50	NM_005950	MT1G
A_24_P260325	0.01	0.01	0.11	0.01	NM_004744	LRAT
A_23_P73632	0.01	0.01	6.63	0.76	NM_000475	NR0B1

A_23_P104438	0.01	0.01	2.17	0.30	NM_032578	MYPN
A_24_P748377	0.01	0.01	0.16	0.01	AL833456	
A_23_P335329	0.51	0.04	11.00	0.99	NM_004485	GNG4
A_23_P159027	0.01	0.01	0.74	0.08	NM_015461	ZNF521
A_23_P167389	0.11	0.01	2.95	0.27	NM_022481	CENTD3
A_24_P526623	0.01	0.01	2.71	0.29	CR606637	
A_23_P428129	0.27	0.02	17.86	1.47	NM_000076	CDKN1C
A_23_P159255	0.01	0.01	14.81	1.86	NM_002845	PTPRM
A_23_P11685	0.01	0.00	0.45	0.05	NM_024420	PLA2G4A
A_24_P273726	0.03	0.01	2.34	0.17	NM_198080	MSRB3
A_23_P106322	0.06	0.01	3.10	0.25	NM_030594	CPEB1
A_24_P65941	0.01	0.01	0.25	0.02	AK024509	
A_24_P240166	0.09	0.01	3.59	0.39	NM_145753	PHLDB2
A_23_P50697	0.01	0.01	0.87	0.09	NM_006905	PSG1
A_23_P136493	0.01	0.01	1.64	0.20	NM_013962	NRG1
A_23_P119353	0.26	0.02	79.88	6.35	NM_017805	RASIP1
A_32_P141418	0.01	0.01	0.25	0.02	NM_018076	ARMC4
A_24_P940365	0.01	0.01	0.21	0.02	BC041481	
A_23_P167559	1.06	0.08	15.02	1.22	NM_144726	RNF145
A_23_P64661	0.01	0.01	0.11	0.01	NM_032496	ARHGAP9
A_23_P127948	0.11	0.01	10.58	1.10	NM_001124	ADM
A_23_P213562	0.01	0.01	0.17	0.01	NM_001992	F2R
A_23_P158318	0.09	0.01	0.47	0.03	NM_004560	ROR2
A_32_P117503	0.01	0.01	0.05	0.01	XM_379201	
A_23_P43175	1.15	0.09	24.44	2.26	NM_144710	10.Sep
A_24_P400324	0.01	0.01	0.14	0.01	AB023177	
A_23_P205031	0.23	0.02	14.13	1.00	NM_001846	COL4A2
A_23_P133902	0.18	0.01	3.62	0.31	NM_014068	PSORS1C1
A_23_P105307	0.14	0.01	0.86	0.06	NM_201444	DGKA
A_32_P416583	0.03	0.01	0.23	0.02	NM_032206	NLRC5
A_24_P639671	0.01	0.01	2.94	0.27	AK095831	
A_32_P224234	0.01	0.01	0.57	0.06	AK123450	
A_23_P82929	0.04	0.01	7.90	1.17	NM_002514	NOV
A_23_P19291	5.65	0.43	125.25	11.56	NM_001069	TUBB2A
A_23_P396135	0.37	0.03	2.50	0.18	NM_004387	NKX2-5
A_23_P254816	0.04	0.01	7.79	0.87	NM_004609	TCF15
A_24_P323148	0.01	0.01	0.38	0.04	NM_182573	LYPD5
A_32_P187571	0.01	0.01	0.94	0.10	NM_004588	SCN2B
A_23_P202658	0.05	0.01	312.10	30.42	NM_000852	GSTP1
A_23_P79518	0.01	0.00	0.46	0.04	NM_000576	IL1B
A_23_P214168	0.01	0.01	3.45	0.44	NM_004370	COL12A1
A_24_P225862	0.01	0.01	0.10	0.01		
A_24_P334726	0.01	0.01	22.58	1.60	NM_015689	DENND2A
A_24_P58549	0.05	0.01	0.27	0.02	NM_021148	ZNF273
A_23_P344281	0.01	0.01	0.17	0.01	NM_001010879	ZIK1
A_23_P257871	0.05	0.01	2.79	0.25	NM_001343	DAB2
A_23_P156890	0.01	0.01	0.12	0.01	NM_003206	TCF21
A_23_P53039	0.05	0.01	3.47	0.42	NM_002301	LDHC
A_23_P92809	0.01	0.01	1.05	0.07	AF067801	
A_23_P20566	0.11	0.01	27.56	1.95	NM_213674	TPM2
A_23_P218569	0.01	0.01	0.19	0.02		
A_24_P851522	0.01	0.01	1.15	0.09	BC015449	
A_23_P66881	0.01	0.01	2.38	0.20	NM_003835	RGS9
A_23_P344568	0.01	0.01	0.23	0.02	NM_145019	FAM124A
A_23_P415984	0.11	0.01	3.53	0.25	NM_002518	NPAS2
A_32_P37867	0.01	0.01	1.10	0.08	AB051431	
A_23_P201706	0.97	0.07	83.76	5.92	NM_005978	S100A2
A_24_P20120	0.03	0.01	0.61	0.04	NM_018084	CCDC88A
A_24_P410453	0.01	0.01	0.25	0.02	NM_033071	SYNE1
A_23_P27556	0.01	0.01	2.54	0.18	NM_001974	EMR1
A_32_P128572	0.05	0.01	0.38	0.03	NM_020948	MIER1
A_23_P250629	0.01	0.01	5.94	0.49	NM_004159	PSMB8
A_23_P88626	0.01	0.01	9.55	0.70	NM_001150	ANPEP
A_24_P237896	0.03	0.01	5.03	0.36		
A_23_P159952	0.01	0.01	34.92	3.62	NM_018476	BEX1
A_32_P180265	0.01	0.01	2.95	0.23		

A_23_P360777	0.01	0.01	2.17	0.16	NM_013957	NRG1
A_23_P301105	0.01	0.01	0.22	0.02	NM_144682	SLFN13
A_24_P347624	0.01	0.01	25.34	2.43	NM_022804	SNURF
A_23_P43095	0.01	0.01	0.96	0.07	NM_024721	ZFHX4
A_23_P127565	0.01	0.01	20.75	1.47	NM_178834	LAYN
A_23_P81219	0.24	0.02	15.91	1.13	NM_016619	PLAC8
A_23_P213877	0.01	0.01	1.17	0.08	NM_023073	
A_23_P118042	0.01	0.01	1.04	0.07	NM_018296	LRRC36
A_23_P40415	0.01	0.01	3.85	0.33	NM_007038	ADAMTS5
A_23_P65518	0.01	0.01	0.33	0.02	NM_016651	DACT1
A_23_P40240	0.01	0.01	8.19	0.80	NM_001336	CTSZ
A_32_P165557	0.01	0.01	0.82	0.06		
A_24_P57047	0.01	0.01	2.91	0.25	NM_203486	DLL3
A_23_P92903	0.02	0.01	1.05	0.07	NM_031908	C1QTNF2
A_24_P110983	0.01	0.01	3.30	0.32	AK055109	AKT3
A_24_P746314	0.01	0.01	0.78	0.06		
A_23_P116614	0.01	0.01	10.32	1.08	NM_001014811	ME3
A_23_P352950	0.61	0.04	13.43	0.95	NM_052926	PNMA5
A_23_P12549	0.01	0.01	2.03	0.14	NM_019043	APBB1IP
A_24_P316430	0.01	0.01	0.89	0.06	NM_002526	NT5E
A_24_P886096	0.01	0.01	0.93	0.07	AK094413	
A_23_P168909	0.01	0.01	0.52	0.04	NM_012082	ZFPM2
A_23_P211561	0.04	0.01	17.89	1.87	AK093341	
A_24_P940149	0.01	0.01	0.82	0.06	NM_199050	C21orf25
A_32_P525524	0.01	0.01	0.74	0.05	NM_178495	
A_23_P377094	0.01	0.01	0.35	0.03	NM_013364	PNMA3
A_24_P88850	0.23	0.02	6.28	0.44	NM_012219	MRAS
A_23_P397293	0.01	0.01	598.90	51.17	NM_017527	LY6K
A_24_P123385	0.01	0.01	0.63	0.05	NM_005909	MAP1B
A_23_P201778	0.01	0.01	2.96	0.21	NM_080588	PTPN7
A_23_P51231	0.01	0.01	1.56	0.13	NM_004350	RUNX3
A_23_P52676	0.01	0.01	1.12	0.09	NM_053054	CATSPER1
A_32_P30649	0.01	0.01	1.19	0.08	NM_004454	ETV5
A_24_P289818	0.38	0.03	1.37	0.10	NM_001011510	FKBP1A
A_23_P76480	0.01	0.01	0.43	0.03	BF213738	CLEC2B
A_23_P254688	0.01	0.01	2.01	0.17	NM_023943	TMEM108
A_23_P157926	0.01	0.01	1.92	0.16	NM_152570	LINGO2
A_24_P921366	0.01	0.01	9.87	1.13	NM_033138	CALD1
A_23_P55198	0.01	0.01	0.97	0.07	NM_173478	CNTD1
A_23_P210361	0.01	0.01	1.04	0.07	NM_022082	C20orf59
A_23_P69030	0.01	0.01	0.55	0.04	NM_001850	COL8A1
A_23_P130948	0.01	0.01	1.18	0.08	NM_002785	PSG11
A_23_P329353	0.01	0.01	1.66	0.12	NM_015463	C2orf32
A_23_P16252	0.16	0.01	1.92	0.14	NM_002257	KLK1
A_32_P79434	0.01	0.01	1.27	0.09	NM_002847	PTPRN2
A_23_P250671	0.13	0.01	262.44	24.69	NM_201397	GPX1
A_24_P882732	0.16	0.01	0.60	0.04		
A_24_P639679	0.01	0.01	0.51	0.04	AK095831	
A_32_P170547	0.03	0.01	11.38	0.80	NM_001007551	
A_23_P119593	2.94	0.21	9.03	0.64	NM_024794	ABHD9
A_23_P216501	1.59	0.11	255.73	18.08	NM_213674	TPM2
A_23_P77066	0.01	0.01	12.36	0.87	NM_022807	SNRPN
A_32_P31666	0.02	0.01	0.37	0.03	AF064804	SUPT3H
A_24_P921446	0.02	0.01	0.90	0.06	BC017854	EMP1
A_23_P87742	0.05	0.01	4.43	0.31	NM_080731	
A_32_P15466	0.01	0.01	16.87	1.36		
A_23_P161727	0.01	0.01	30.35	2.15	NM_001541	HSPB2
A_23_P65506	0.04	0.01	0.26	0.02	NM_000347	SPTB
A_23_P2181	0.01	0.01	21.11	1.74	NM_001001336	
A_24_P554882	0.01	0.01	1.18	0.08		
A_23_P171074	0.01	0.01	2.60	0.18	NM_004867	ITM2A
A_23_P156562	0.01	0.01	0.08	0.01		
A_24_P303480	0.01	0.01	8.48	0.60	NM_006834	RAB32
A_32_P55462	0.40	0.03	10.57	0.75	CR593500	
A_23_P203882	0.01	0.01	0.96	0.07	NM_022791	
A_32_P138348	0.01	0.01	240.83	19.17	NM_017527	LY6K

A_23_P15146	0.04	0.01	5.15	0.36	NM_001012631	IL32
A_32_P199292	0.22	0.02	0.44	0.03	BC071966	FAM60A
A_32_P378035	0.01	0.01	0.08	0.01	AB051443	
A_23_P166779	0.04	0.01	0.19	0.02	NM_013343	LOH3CR2A
A_24_P77968	0.01	0.01	17.27	1.22	NM_001458	FLNC
A_23_P325606	0.01	0.01	0.77	0.05	NM_153448	ESX1
A_24_P271696	0.01	0.01	164.98	13.47	NM_133431	XAGE1
A_32_P780817	0.01	0.01	1.36	0.10	NM_001017417	
A_32_P138032	0.02	0.01	0.39	0.03	NM_006365	C1orf61
A_23_P324916	0.01	0.01	0.35	0.03	NM_013992	PAX8
A_23_P5103	0.01	0.01	3.54	0.26	NM_213633	PSG4
A_32_P50275	0.11	0.01	0.82	0.06	AK095453	CD59
A_23_P43484	0.01	0.00	86.47	8.71	NM_058197	CDKN2A
A_24_P274814	0.02	0.01	0.29	0.02	NM_030984	TBXAS1
A_24_P942312	0.01	0.01	0.05	0.01		
A_23_P94501	0.19	0.02	120.44	9.38	NM_000700	ANXA1
A_24_P334130	0.01	0.01	2.01	0.14	NM_054034	FN1
A_23_P401700	0.01	0.01	4.92	0.36	NM_019043	APBB1IP
A_24_P206776	0.07	0.01	15.05	1.06	NM_001885	CRYAB
A_24_P372613	0.14	0.01	2.27	0.16	NM_001164	APBB1
A_32_P27706	0.01	0.01	2.56	0.19		
A_24_P331128	0.01	0.01	1.71	0.12	NM_002068	GNA15
A_23_P132956	0.10	0.01	274.19	26.43	NM_004181	UCHL1
A_23_P53476	0.01	0.01	304.02	31.12	NM_002300	LDHB
A_32_P137604	0.01	0.01	2.82	0.20	BC018597	
A_24_P466590	0.01	0.01	3.60	0.25		
A_32_P97169	0.01	0.01	2.56	0.19	BX640888	
A_23_P209799	0.01	0.01	1.28	0.09	BC035615	
A_32_P43826	0.01	0.01	0.13	0.01	AL832540	
A_23_P47924	0.01	0.01	0.64	0.05	NM_002849	PTPRR
A_32_P377880	0.01	0.01	0.27	0.02	BC008580	
A_23_P161190	1.01	0.07	216.85	15.33	NM_003380	VIM
A_32_P133670	1.04	0.07	2.91	0.21	NM_006305	ANP32A
A_32_P154053	0.01	0.01	0.12	0.01	NM_173681	ATG9B
A_24_P940509	0.01	0.01	0.25	0.02	AK022793	
A_23_P125233	0.01	0.01	5.64	0.40	NM_001299	CNN1
A_23_P329962	0.01	0.01	5.53	0.39	NM_152782	SUNC1
A_23_P314052	0.01	0.01	8.00	0.57	NM_015431	TRIM58
A_23_P102706	0.01	0.01	3.27	0.23	NM_014723	SNPH
A_23_P205428	0.01	0.01	4.90	0.36	NM_005249	FOXG1
A_23_P27332	0.01	0.01	1.78	0.13	NM_003199	TCF4
A_23_P430068	0.01	0.01	3.45	0.24	NM_006474	PDPN
A_23_P205519	0.95	0.07	4.66	0.33	NM_022060	ABHD4
A_23_P433229	0.01	0.01	0.19	0.02	NM_014759	PHYHIP
A_23_P425073	0.30	0.02	2.37	0.17	NM_002898	RBMS2
A_24_P498854	0.01	0.01	0.91	0.08	AK096621	
A_24_P58620	0.01	0.01	0.32	0.02	AY358924	
A_24_P870509	0.01	0.01	0.36	0.03	AF086261	
A_24_P233786	0.25	0.02	7.25	0.55	NM_052966	
A_24_P941167	0.01	0.01	1.27	0.12	AK074645	APOL6
A_23_P8175	0.26	0.02	6.07	0.44	NM_006718	PLAGL1
A_32_P103220	0.01	0.01	0.38	0.03	NM_030955	ADAMTS12
A_32_P88240	0.01	0.01	0.59	0.04	NM_207335	KLHDC6
A_23_P360605	0.05	0.01	1.17	0.08	AB018345	KIAA0802
A_32_P57013	0.01	0.01	2.29	0.16	BU540282	
A_23_P132763	0.01	0.01	3.99	0.44	NM_016206	VGLL3
A_23_P95672	0.01	0.01	0.43	0.04	NM_006548	IGF2BP2
A_23_P16438	0.01	0.01	0.17	0.01	NM_016941	DLL3
A_32_P117322	0.01	0.01	0.24	0.02		
A_32_P21255	0.01	0.01	0.27	0.02	NM_013309	SLC30A4
A_24_P294851	0.01	0.01	0.25	0.02	NM_006355	TRIM38
A_32_P42705	0.01	0.01	0.59	0.05	BC017721	
A_23_P107432	0.02	0.01	1.00	0.07	NM_207453	
A_23_P73589	0.01	0.01	19.79	2.72	NM_002444	MSN
A_23_P79398	0.02	0.01	2.29	0.24	NM_004633	IL1R2
A_24_P185854	0.01	0.01	0.76	0.07	NM_004010	DMD

A_23_P168188	0.59	0.04	15.59	1.10	NM_001009991	SYTL3
A_23_P136173	0.01	0.01	0.83	0.07	NM_172247	CSF2RA
A_23_P92467	0.01	0.01	0.61	0.05	NM_198179	GPR103
A_32_P217750	0.01	0.01	0.40	0.03	NM_002183	IL3RA
A_23_P120667	0.03	0.01	2.30	0.17	NM_021219	JAM2
A_23_P143981	0.01	0.01	6.68	0.68	NM_001004019	FBLN2
A_23_P46936	0.01	0.01	0.39	0.03	NM_000399	EGR2
A_23_P35912	0.28	0.02	68.77	6.14	NM_033306	CASP4
A_23_P364024	0.01	0.01	1.95	0.16	NM_006851	GLIPR1
A_23_P2831	0.01	0.01	0.90	0.08	NM_003991	EDNRB
A_23_P92909	0.01	0.01	0.10	0.01	NM_205841	SPINK6
A_24_P532232	0.01	0.01	0.17	0.01	NM_182898	CREB5
A_23_P404494	0.01	0.01	1.91	0.20	NM_002185	IL7R
A_32_P24382	0.04	0.01	2.42	0.18	BC063625	KRTAP2-4
A_32_P117016	0.02	0.01	1.97	0.18	CR749561	ALDH1L2
A_32_P202502	0.01	0.01	1.04	0.10	NM_181723	EFHA2
A_23_P157963	0.01	0.01	0.15	0.01	NM_017738	C9orf39
A_23_P307002	4.02	0.28	15.45	1.09	AK023110	SSU72
A_23_P218646	0.92	0.07	96.29	8.71	NM_032945	TNFRSF6B
A_23_P109143	0.85	0.06	11.51	0.92	NM_000311	PRNP
A_23_P257583	0.02	0.01	32.63	2.31	NM_015689	DENND2A
A_23_P45560	0.76	0.05	7.65	0.54	NM_000273	GPR143
A_24_P76809	0.01	0.01	0.23	0.02	BC035960	PTPRO
A_23_P158231	0.03	0.01	2.50	0.18	NM_005503	APBA2
A_32_P181222	0.04	0.01	0.53	0.04	NM_002247	KCNMA1
A_32_P169847	0.01	0.01	0.20	0.02		
A_23_P123402	0.02	0.01	0.74	0.07	NM_184086	TRIM55
A_23_P84448	0.84	0.06	10.94	0.77	NM_025019	
A_32_P358887	0.01	0.01	0.34	0.03	NM_003759	SLC4A4
A_23_P136623	0.01	0.01	0.89	0.07	NM_183357	ADCY5
A_23_P73747	0.09	0.01	18.39	2.00	NM_014782	ARMCX2
A_23_P11331	0.01	0.01	18.47	2.27	NM_153333	TCEAL8
A_23_P363275	0.02	0.01	4.94	0.35	NM_144668	WDR66
A_23_P352402	1.77	0.13	37.29	2.64	NM_153256	C10orf47
A_23_P209564	0.05	0.01	3.25	0.31	NM_024843	CYBRD1
A_32_P150876	0.01	0.01	0.36	0.03	BC041875	
A_24_P408736	0.01	0.01	0.12	0.01	NM_014568	GALNT5
A_23_P16469	0.29	0.02	9.26	0.68	NM_001005377	PLAUR
A_24_P354689	0.01	0.01	1.04	0.10	NM_004598	SPOCK1
A_32_P221000	0.01	0.01	3.54	0.25	AK094154	
A_24_P205458	0.25	0.02	1.60	0.11	NM_003607	CDC42BPA
A_32_P136516	0.01	0.01	0.36	0.03	BC035720	
A_23_P431776	0.08	0.01	1.25	0.09	NM_001986	ETV4
A_24_P305784	0.01	0.01	0.80	0.07	NM_145664	SPANXB2
A_23_P56505	0.01	0.01	0.42	0.03	NM_000885	ITGA4
A_23_P83328	0.07	0.01	1.25	0.09	NM_000118	ENG
A_24_P333993	0.01	0.01	2.17	0.21	NM_001001957	OR2W3
A_23_P105873	0.01	0.01	1.09	0.09	NM_016179	TRPC4
A_24_P393312	0.01	0.01	2.17	0.20	AK090554	KIRREL
A_23_P250516	0.01	0.01	0.75	0.06	XM_211339	
A_23_P80763	0.01	0.01	1.09	0.08	AL050071	PVRL3
A_23_P22735	3.62	0.26	126.98	8.98	NM_032621	BEX2
A_32_P30339	0.01	0.01	0.07	0.01		
A_23_P24414	0.11	0.01	6.27	0.44	NM_016938	EFEMP2
A_23_P133543	0.01	0.01	0.17	0.01	NM_017415	KLHL3
A_24_P153643	0.01	0.01	0.33	0.02	NM_004947	DOCK3
A_24_P815062	0.01	0.01	0.66	0.05		
A_23_P42575	0.01	0.01	2.50	0.24	NM_033138	CALD1
A_23_P88602	0.01	0.01	0.76	0.05	NM_170677	MEIS2
A_32_P168727	0.01	0.01	6.74	0.72		
A_24_P561165	0.01	0.01	0.59	0.04		SERPING1
A_24_P100613	0.01	0.01	4.17	0.43	NM_005559	LAMA1
A_23_P159893	0.01	0.01	0.70	0.05	NM_145234	CHRDL1
A_23_P74088	0.12	0.01	17.19	1.22	NM_006983	MMP23B
A_23_P368645	0.01	0.01	2.25	0.21	NM_003659	AGPS
A_23_P406508	0.01	0.01	0.67	0.05	NM_005020	PDE1C

A_23_P166823	0.06	0.01	1.78	0.13	NM_003280	TNNC1
A_24_P55295	0.02	0.01	2.17	0.21	NM_000165	GJA1
A_24_P311917	0.01	0.01	0.25	0.02	NM_006994	BTN3A3
A_23_P258393	0.35	0.03	0.82	0.06	NM_144641	PPM1M
A_23_P162165	0.01	0.01	1.68	0.12	NM_023930	KCTD14
A_23_P300600	0.09	0.01	173.51	18.94	NM_021076	NEFH
A_24_P923142	0.01	0.01	2.74	0.26	BC020784	ZC3HAV1L
A_23_P103532	0.22	0.02	1.61	0.11	NM_007369	GPR161
A_23_P66241	0.01	0.01	0.74	0.07	NM_176870	MT1M
A_23_P57658	0.01	0.01	4.02	0.29	NM_020386	HRASLS
A_23_P395438	0.02	0.01	1.84	0.17	NM_053044	HTRA3
A_24_P758010	0.01	0.01	0.05	0.01	AJ318805	
A_23_P105227	0.01	0.01	0.65	0.06	NM_001014811	ME3
A_23_P32444	0.14	0.01	3.89	0.27	NM_032348	MXRA8
A_24_P170983	0.11	0.01	14.03	1.27	NM_194312	ESPNL
A_23_P388168	0.01	0.01	1.77	0.13	NM_002867	RAB3B
A_23_P161563	0.01	0.01	0.42	0.03	NM_022337	RAB38
A_24_P548966	0.18	0.02	7.18	0.56		
A_24_P75948	0.10	0.01	0.38	0.03		HSP90B1
A_23_P8754	0.01	0.01	1.26	0.12	NM_005763	AASS
A_23_P312132	0.01	0.01	0.22	0.02	NM_000887	ITGAX
A_32_P36715	0.01	0.01	0.35	0.03	CR627135	
A_23_P70598	0.01	0.01	1.60	0.16	NM_018679	TCP11
A_32_P228618	0.01	0.01	1.06	0.10	NM_001003793	RBMS3
A_32_P94160	0.01	0.01	0.19	0.02	BC043195	
A_23_P417363	0.01	0.01	0.83	0.06	AK024722	CLIP4
A_32_P5800	0.09	0.01	0.69	0.05	CR602075	
A_23_P39925	0.07	0.01	2.15	0.15	NM_003494	DYSF
A_23_P23924	1.78	0.15	88.67	6.85	NM_001748	CAPN2
A_23_P31273	0.05	0.01	1.27	0.09	NM_001635	AMPH
A_23_P333228	0.01	0.01	0.64	0.05	NM_020814	4.Mar
A_23_P72001	0.09	0.01	6.39	0.45	NM_174933	PHYHD1
A_23_P345674	0.01	0.01	1.26	0.12	NM_021216	ZNF71
A_23_P209167	0.58	0.04	5.85	0.41	NM_005860	FSTL3
A_23_P9836	0.01	0.01	0.76	0.05	NM_004454	ETV5
A_23_P65678	0.01	0.01	4.76	0.37	NM_000138	FBN1
A_32_P40288	0.01	0.01	4.18	0.44	NM_052913	KIAA1913
A_24_P683011	0.01	0.01	0.96	0.07	BM696546	EFCBP1
A_23_P390755	0.01	0.01	0.10	0.01	AK098558	C8orf45
A_24_P56240	0.01	0.01	0.43	0.05	NM_153634	CPNE8
A_23_P200260	0.90	0.06	4.31	0.30	NM_014801	PCNXL2
A_32_P120851	0.01	0.01	0.08	0.01		
A_23_P156826	0.01	0.01	0.48	0.05	NM_032744	C6orf105
A_32_P183718	0.08	0.01	2.15	0.23	NM_001003845	SP5
A_32_P42236	0.91	0.07	3.69	0.26	XM_370839	
A_23_P90357	0.16	0.01	1.43	0.10	NM_201636	TBXA2R
A_32_P234853	0.01	0.01	0.09	0.01		
A_23_P214185	0.83	0.06	4.29	0.31	NM_016021	UBE2J1
A_23_P311875	0.01	0.01	0.22	0.02	NM_006725	CD6
A_24_P152983	70.57	4.99	155.72	11.01		
A_24_P194688	0.01	0.01	0.08	0.01	NM_181723	EFHA2
A_24_P3045	0.01	0.01	0.06	0.01	NM_032974	CASP10
A_24_P208567	0.01	0.01	0.33	0.03	NM_003855	IL18R1
A_24_P930327	0.20	0.02	0.45	0.03		
A_23_P51105	0.05	0.01	0.97	0.12	NM_016441	CRIM1
A_32_P69149	0.04	0.01	6.71	0.64	NM_012449	STEAP1
A_23_P321307	0.01	0.01	0.07	0.01	NM_021599	ADAMTS2
A_24_P156113	1.75	0.12	7.70	0.54	NM_014601	EHD2
A_24_P247902	0.08	0.01	1.09	0.12	AB011131	
A_23_P75063	0.01	0.01	0.22	0.02	NM_032372	DYDC2
A_23_P201547	0.01	0.01	0.56	0.07	NM_014917	NTNG1
A_23_P163782	17.50	1.24	50.23	3.55	NM_005951	MT1H
A_24_P902100	0.01	0.01	0.76	0.09	AL359559	
A_24_P125096	18.00	1.27	53.09	3.75	NM_005952	MT1X
A_23_P9166	0.21	0.02	0.55	0.04	NM_006289	TLN1
A_32_P213342	0.01	0.01	4.02	0.58	BC019241	

A_23_P416766	0.01	0.01	0.14	0.01	NM_003787	NOL4
A_23_P404259	0.51	0.04	3.40	0.26	NM_001008397	
A_24_P291814	0.01	0.01	0.45	0.06	NM_004370	COL12A1
A_32_P156851	0.03	0.01	0.91	0.07	NM_005822	DSCR1L1
A_32_P310335	0.01	0.01	0.28	0.03	AK056079	
A_23_P19226	0.33	0.03	3.71	0.26	NM_013352	DSE
A_24_P416370	0.01	0.01	4.70	0.43	NM_024015	HOXB4
A_24_P621701	0.08	0.01	0.67	0.06		
A_23_P131089	0.29	0.02	2.33	0.18	NM_198471	ANKRD47
A_23_P380298	0.07	0.01	0.60	0.06	NM_014731	
A_24_P379820	1.70	0.13	14.34	1.36	NM_030926	ITM2C
A_23_P166360	1.72	0.12	18.78	1.81	NM_206956	PRAME
A_24_P166613	0.75	0.08	10.44	1.22	NM_017549	EPDR1
A_23_P387630	0.17	0.02	1.41	0.13	NM_014725	STARD8
A_23_P51213	0.01	0.01	0.65	0.08	NM_152372	MYOM3
A_24_P277211	0.01	0.01	0.47	0.05	NM_000861	HRH1
A_23_P217763	0.08	0.01	0.74	0.07	AK055976	TMSB4X
A_23_P434430	0.01	0.01	0.13	0.01	NM_152262	ZNF439
A_32_P226205	0.28	0.02	0.80	0.06	NM_033400	
A_23_P415006	1.00	0.07	8.23	0.73	NM_015470	RAB11FIP5
A_23_P142849	0.14	0.01	2.44	0.28	NM_005168	RND3
A_23_P27035	0.09	0.01	5.23	0.45	NM_015544	TMEM98
A_23_P427703	14.75	1.04	49.51	3.50	X97261	MT1L
A_24_P343233	1.78	0.05	11.17	0.60	NM_002124	HLA-DRB1
A_32_P358264	0.01	0.01	0.08	0.01	BC036441	
A_32_P226525	0.01	0.01	0.41	0.04	BE004814	SOX9
A_32_P65571	0.01	0.01	0.43	0.05	BX648855	
A_23_P85209	0.01	0.01	0.63	0.06	NM_000640	IL13RA2
A_23_P84744	0.01	0.01	0.06	0.01	NM_017558	HYDIN
A_24_P117294	0.02	0.01	2.19	0.24	NM_002463	MX2
A_23_P383679	0.01	0.01	0.08	0.01	AK026688	HYDIN
A_23_P1691	0.02	0.00	268.60	28.11	NM_002421	MMP1
A_23_P303455	0.02	0.01	1.25	0.09	NM_153832	GPR161
A_24_P228717	0.67	0.05	2.60	0.18	NM_002872	RAC2
A_24_P662636	0.01	0.01	0.14	0.01	AW134546	
A_23_P374104	0.01	0.01	0.26	0.03	NM_012098	ANGPTL2
A_23_P203475	0.08	0.01	35.97	3.06	NM_145040	PRKCDBP
A_23_P135273	0.46	0.03	1.60	0.11	NM_001005336	DNM1
A_32_P47874	0.06	0.01	4.77	0.34		
A_23_P139912	0.36	0.03	11.55	1.14	NM_002178	IGFBP6
A_23_P133656	0.01	0.01	2.65	0.34	NM_002290	LAMA4
A_32_P167212	0.06	0.01	2.05	0.27		
A_23_P202484	0.82	0.06	2.90	0.20	NM_032772	ZNF503
A_32_P34372	0.01	0.01	0.06	0.01	CA840930	CPE
A_23_P63798	2.38	0.20	31.32	3.25	NM_001300	
A_23_P23074	0.02	0.01	1.92	0.26	NM_006417	IFI44
A_23_P140394	0.28	0.02	0.90	0.06	NM_004554	NFATC4
A_23_P78518	0.17	0.01	2.86	0.20	AK128234	
A_24_P111106	0.01	0.01	1.06	0.10	NM_000800	FGF1
A_23_P350295	10.05	0.71	69.44	5.33	CR594843	
A_23_P37983	16.33	1.15	49.49	3.50	NM_005947	MT1B
A_23_P92727	0.48	0.05	6.97	0.75	NM_015577	RAI14
A_23_P429383	0.02	0.01	0.31	0.02	NM_014213	HOXD9
A_23_P63038	1.82	0.13	6.66	0.47	NM_022356	LEPRE1
A_23_P210158	0.01	0.01	1.18	0.12		
A_24_P82032	0.01	0.01	0.18	0.02	NM_020663	RHOJ
A_24_P205045	0.03	0.01	0.58	0.07	NM_015576	ERC2
A_23_P34142	0.01	0.01	3.70	0.37	NM_016303	WBP5
A_23_P330611	0.14	0.01	1.01	0.08	NM_003387	WIPF1
A_23_P146644	23.88	1.69	262.26	22.79	NM_001002857	ANXA2
A_23_P106389	0.01	0.01	0.24	0.03	NM_003612	SEMA7A
A_32_P178945	0.03	0.01	0.89	0.11	NM_018566	YOD1
A_24_P221323	0.04	0.01	0.21	0.02	AK092355	
A_23_P386364	0.01	0.01	0.12	0.01	NM_144657	CXorf43
A_23_P20743	0.01	0.01	3.86	0.36	NM_032342	C9orf125
A_23_P384635	0.01	0.01	0.34	0.03	NM_173543	DZIP1L

A_23_P200976	3.41	0.26	22.97	1.62	NM_031207	HYI
A_24_P68088	0.01	0.01	4.41	0.51	AB026156	
A_23_P154037	0.08	0.01	1.55	0.17	NM_001159	AOX1
A_24_P290354	0.93	0.07	7.51	0.67	NM_006007	ZFAND5
A_23_P27315	0.68	0.05	10.02	0.97	NM_032048	EMILIN2
A_32_P208733	0.01	0.01	0.05	0.01	AK055279	C8orf53
A_23_P370707	0.67	0.05	4.61	0.33		
A_23_P258814	1.04	0.09	8.35	0.64		
A_23_P107322	0.26	0.02	0.85	0.06	NM_032854	CORO6
A_23_P259692	1.33	0.09	23.29	2.14	NM_058179	PSAT1
A_23_P35995	0.03	0.01	5.50	0.47	NM_024769	
A_24_P225534	0.02	0.01	0.61	0.08	NM_017821	RHBDL2
A_24_P15621	0.64	0.05	4.14	0.29	NM_198857	
A_23_P389391	0.19	0.02	4.52	0.32	NM_005072	SLC12A4
A_24_P745883	0.30	0.02	1.00	0.07		
A_23_P82523	0.01	0.00	2.09	0.28	NM_000927	ABCB1
A_32_P175301	0.76	0.08	8.91	0.86	NM_014957	DENND3
A_24_P57631	0.02	0.01	0.95	0.14	NM_004484	GPC3
A_23_P39202	2.93	0.21	34.49	2.78	NM_033520	C19orf33
A_23_P86059	0.04	0.01	0.15	0.01	BC007638	KIAA1822L
A_32_P206839	0.80	0.06	5.32	0.42	AF075027	
A_32_P118568	0.01	0.01	0.68	0.08	AJ010230	
A_23_P49254	0.18	0.02	4.28	0.44	NM_005331	HBQ1
A_24_P291826	0.20	0.02	5.71	0.50	NM_001009991	SYTL3
A_24_P383762	0.08	0.01	0.51	0.04	NM_175062	
A_23_P122052	0.70	0.05	4.22	0.35	NM_001008397	
A_23_P67127	0.21	0.02	2.12	0.15	NM_173633	TMEM145
A_23_P141035	0.07	0.01	0.32	0.02	NM_005769	CHST4
A_23_P310590	0.26	0.02	1.33	0.10	NM_178507	OAF
A_23_P208389	0.19	0.02	63.70	4.50	NM_021913	AXL
A_24_P184295	0.76	0.06	3.52	0.25	AF099011	EHD1
A_24_P85300	0.02	0.01	0.38	0.05	BC004539	HEG1
A_24_P935652	0.09	0.01	0.77	0.07	CR606629	NUB1
A_32_P33802	0.01	0.01	1.56	0.11	AA219130	ETV5
A_24_P29733	0.04	0.01	0.23	0.02	NM_012395	PFTK1
A_24_P6921	3.50	0.26	17.23	1.22	NM_001013744	
A_23_P111260	0.01	0.01	1.43	0.17	NM_002526	NT5E
A_23_P42065	0.04	0.01	0.42	0.03	NM_014452	TNFRSF21
A_24_P540560	0.02	0.01	0.15	0.01		RDX
A_23_P67162	9.17	0.65	19.77	1.40	NM_006221	PIN1
A_24_P334445	29.82	2.11	209.25	17.97	NM_001017405	MAEA
A_24_P126282	0.01	0.01	0.06	0.01	NM_194441	BTN3A1
A_23_P145752	0.01	0.01	0.07	0.01	NM_153620	HOXA1
A_24_P313354	6.71	0.47	37.36	2.89	NM_021005	NR2F2
A_24_P514678	0.01	0.01	0.27	0.02		
A_23_P111995	0.09	0.00	15.88	1.72	NM_002318	LOXL2
A_23_P24104	0.02	0.00	115.28	14.54	NM_002658	PLAU
A_23_P160559	0.03	0.01	2.86	0.20	NM_004425	ECM1
A_24_P551302	0.01	0.01	1.34	0.12	BC038432	
A_32_P164477	0.01	0.01	0.19	0.02	AI379175	C9orf19
A_23_P149562	0.05	0.01	0.56	0.06	NM_004815	ARHGAP29
A_23_P7791	0.99	0.09	8.64	0.90	NM_024576	OGFRL1
A_23_P120243	0.02	0.01	0.27	0.02	NM_024501	HOXD1
A_23_P214603	15.11	1.07	39.06	2.76	NM_005803	FLOT1
A_24_P297166	0.01	0.01	0.13	0.01	NM_001009939	
A_24_P295633	0.03	0.01	0.17	0.01	NM_024841	
A_24_P942933	0.01	0.01	0.08	0.01	AK022802	APOBEC3G
A_23_P202427	0.01	0.01	3.84	0.52	NM_025130	HKDC1
A_24_P168925	0.01	0.01	6.95	0.53	CR623913	CHRDL1
A_23_P2492	0.03	0.01	3.02	0.21	NM_001734	C1S
A_23_P130158	0.59	0.04	4.21	0.36	NM_030753	WNT3
A_23_P386320	0.19	0.02	2.07	0.21	NM_033316	MFI2
A_24_P246406	0.02	0.01	0.20	0.02	AK027146	FAM69A
A_24_P204690	0.01	0.01	0.24	0.02	NM_001013692	PRAMEF3
A_23_P327022	0.01	0.01	0.20	0.02	NM_199072	MDFIC
A_23_P510	0.01	0.01	0.06	0.01	NM_152666	PLD5

A_24_P82142	0.28	0.02	1.67	0.13	NM_207038	TCF12
A_23_P129246	0.73	0.05	6.68	0.56	NM_025201	PLEKHQ1
A_23_P156687	0.03	0.01	0.62	0.08	NM_001710	CFB
A_23_P75310	0.06	0.01	1.93	0.16	NM_021226	ARHGAP22
A_23_P22224	15.08	1.07	59.05	4.18	NM_004095	EIF4EBP1
A_23_P26426	0.70	0.05	4.18	0.31	NM_014427	CPNE7
A_23_P414343	20.33	1.44	61.44	4.34	NM_005951	MT1H
A_23_P82979	0.36	0.03	8.66	0.61	NM_006059	LAMC3
A_24_P334361	0.01	0.01	0.06	0.01	NM_017631	
A_23_P165636	12.25	0.87	134.68	11.76	NM_001747	CAPG
A_24_P76831	0.01	0.01	0.05	0.01	NM_000578	SLC11A1
A_32_P141768	0.01	0.01	1.97	0.27	CR627395	
A_24_P65864	2.68	0.19	8.99	0.64	NM_016143	NSFL1C
A_23_P213336	0.01	0.01	0.66	0.09	NM_000800	FGF1
A_32_P70626	0.02	0.01	0.29	0.02		
A_23_P67151	0.22	0.02	0.78	0.06	NM_058164	OLFM2
A_23_P19754	0.01	0.01	0.21	0.02	NM_016352	CPA4
A_32_P147830	0.01	0.01	0.11	0.01		
A_23_P122393	0.11	0.01	1.31	0.09	NM_206539	EGFL9
A_24_P366787	0.10	0.01	0.57	0.04	BC029785	
A_24_P326660	0.14	0.01	7.41	0.75	NM_006500	MCAM
A_23_P74609	0.01	0.01	0.96	0.12	NM_015714	G0S2
A_23_P75790	0.68	0.05	9.08	0.72	NM_013279	C11orf9
A_24_P30194	0.06	0.01	0.74	0.08	NM_012420	IFIT5
A_24_P148261	0.04	0.01	0.55	0.06	AK021874	
A_23_P35456	0.10	0.01	6.78	0.91	NM_014631	SH3PXD2A
A_24_P712350	0.30	0.03	2.15	0.19	NM_001821	CHML
A_23_P51215	0.01	0.01	0.22	0.02	NM_152372	MYOM3
A_32_P103726	0.04	0.01	0.59	0.07		
A_23_P59877	4.69	0.38	37.00	3.43	NM_001444	FABP5
A_32_P132748	0.01	0.01	2.26	0.21	BC018626	
A_24_P717586	0.01	0.01	0.09	0.01		
A_23_P303242	22.66	1.60	64.87	4.59	NM_005952	MT1X
A_24_P63019	0.01	0.01	0.08	0.01	NM_004633	IL1R2
A_23_P106425	0.16	0.02	1.22	0.10	NM_182616	C15orf38
A_32_P143000	0.05	0.01	0.31	0.02	AB011146	
A_24_P341731	0.12	0.01	2.31	0.16		
A_23_P429998	0.02	0.01	0.06	0.01	NM_006732	FOSB
A_24_P124349	0.01	0.01	0.07	0.01	NM_025208	PDGFD
A_23_P206960	2.86	0.30	27.40	2.74	NM_003003	SEC14L1
A_23_P302787	0.22	0.02	1.68	0.16	BC013438	
A_23_P61371	0.09	0.01	9.00	0.64	NM_198282	TMEM173
A_23_P331479	1.66	0.16	14.00	1.27	AB075829	
A_23_P137173	1.65	0.12	11.14	0.95	NM_021992	TMSL8
A_23_P154367	0.19	0.02	0.40	0.03	NM_004226	STK17B
A_24_P371281	0.01	0.01	1.13	0.16	NM_025246	TMEM22
A_23_P64980	0.01	0.01	0.10	0.01	NM_016615	SLC6A13
A_23_P324011	0.02	0.01	0.29	0.04	BC008001	
A_24_P73943	0.14	0.01	0.62	0.04	NM_004375	COX11
A_23_P32175	0.72	0.05	3.14	0.22	NM_014368	LHX6
A_23_P13740	0.01	0.01	0.26	0.03	AB023155	NAV3
A_23_P211926	0.01	0.01	0.82	0.10	NM_003392	WNT5A
A_24_P520767	0.10	0.01	1.22	0.10	BC036441	
A_24_P307248	0.31	0.02	0.82	0.06	NM_001013617	
A_24_P11737	0.57	0.04	1.30	0.09	AK095678	
A_23_P126782	0.01	0.01	0.96	0.13	NM_001993	F3
A_23_P15394	0.10	0.01	2.60	0.29	NM_001251	CD68
A_24_P17870	0.05	0.01	1.15	0.09	NM_006674	HCP5
A_24_P344416	0.01	0.01	0.14	0.01	NM_024423	DSC3
A_32_P214565	0.01	0.01	1.15	0.15		EFCBP1
A_23_P23639	0.07	0.01	0.71	0.07	NM_153259	MCOLN2
A_24_P763655	8.26	0.65	114.37	8.09		
A_23_P22614	0.04	0.01	2.82	0.35	NM_145802	6.Sep
A_24_P34155	0.01	0.01	0.19	0.02	X90980	
A_23_P729	0.01	0.01	0.13	0.01	NM_003176	SYCP1
A_23_P104073	0.04	0.01	0.37	0.03	NM_002960	S100A3

A_23_P92929	0.01	0.01	0.52	0.05	NM_016644	PRR16
A_32_P151102	0.01	0.01	0.10	0.01	NM_002444	MSN
A_23_P110266	0.01	0.01	0.25	0.03	NM_024574	C4orf31
A_23_P95930	0.01	0.01	2.42	0.19	NM_001015886	
A_23_P163475	0.37	0.03	6.93	0.69	NM_207380	
A_23_P120227	0.01	0.01	0.16	0.01	NM_030915	LBH
A_32_P12232	0.01	0.01	1.00	0.11		
A_32_P933041	0.01	0.01	0.11	0.01	AK093435	
A_24_P467073	0.01	0.01	0.10	0.01	AK131023	
A_24_P308029	0.05	0.01	0.48	0.04	NM_144617	HSPB6
A_23_P162668	0.05	0.01	0.72	0.06	NM_001874	CPM
A_23_P160354	0.01	0.01	0.82	0.07	NM_181690	AKT3
A_24_P192978	0.24	0.02	4.55	0.32	NM_173583	
A_24_P298179	0.67	0.05	2.08	0.15		
A_32_P232747	0.12	0.01	0.74	0.05	BC030024	ASNS
A_23_P403488	0.01	0.01	0.17	0.02	NM_176821	NLRP10
A_24_P915464	0.01	0.01	0.11	0.01		
A_23_P29551	0.97	0.07	7.35	0.52	NM_018306	TMEM40
A_32_P164203	0.01	0.01	1.35	0.13		
A_24_P409661	4.83	0.34	15.35	1.09		
A_23_P27040	0.07	0.01	3.51	0.41	NM_015544	TMEM98
A_32_P48842	0.01	0.01	0.95	0.09	CR595668	
A_23_P164706	0.01	0.01	0.62	0.07	NM_003451	ZNF177
A_23_P256948	0.01	0.01	0.10	0.01	NM_005098	MSC
A_24_P212531	0.22	0.02	0.87	0.06	NM_152490	B3GALNT2
A_32_P167176	0.34	0.03	2.40	0.17		
A_23_P151166	0.01	0.01	0.13	0.01	NM_032369	HVCN1
A_24_P282377	0.01	0.01	0.17	0.02	AL713660	
A_24_P643028	0.08	0.01	0.51	0.04	NM_018088	FAM90A1
A_23_P9883	0.01	0.01	0.66	0.08	NM_004895	NLRP3
A_23_P71699	0.01	0.01	0.14	0.01	NM_006914	RORB
A_23_P434919	0.01	0.01	0.59	0.05	NM_152304	RAB42
A_23_P78867	0.01	0.01	0.86	0.06	NM_000540	RYR1
A_23_P96383	0.05	0.01	3.96	0.49	NM_006307	SRPX
A_24_P279704	0.11	0.01	0.30	0.02	NM_080600	MAG
A_24_P10214	0.12	0.01	5.78	0.75	NM_014178	STXBP6
A_23_P209856	0.02	0.01	0.82	0.09	NM_017488	ADD2
A_23_P18490	2.70	0.21	22.39	1.66	NM_001017405	MAEA
A_23_P110957	0.01	0.01	0.66	0.07	NM_001452	FOXF2
A_32_P150748	0.01	0.01	0.19	0.02	G31710	
A_23_P502363	0.01	0.01	0.10	0.01	NM_000503	EYA1
A_24_P943802	0.24	0.02	1.15	0.08	AK128047	
A_23_P24083	0.01	0.01	0.91	0.11	NM_005396	PNLIPRP2
A_23_P103672	0.34	0.03	2.76	0.21	NM_006617	NES
A_32_P167239	0.01	0.01	3.15	0.31	NM_152406	AFAP1L1
A_23_P141429	0.05	0.01	6.52	0.47	NM_016428	ABI3
A_23_P210001	2.05	0.17	45.48	4.86	NM_003466	PAX8
A_23_P252556	0.01	0.01	0.50	0.06	NM_001704	BAI3
A_24_P208345	0.41	0.03	1.90	0.13	NM_033102	SLC45A3
A_23_P206724	7.77	0.55	30.86	2.18	NM_175617	MT1E
A_23_P48951	0.08	0.01	1.52	0.11	NM_005928	MFGE8
A_24_P358591	0.02	0.01	0.52	0.04	AK091483	
A_23_P80040	1.55	0.11	23.48	1.82	NM_006404	PROCR
A_23_P212179	0.01	0.01	0.64	0.05	NM_000861	HRH1
A_23_P35444	0.05	0.01	1.33	0.14	NM_032727	INA
A_24_P347480	0.05	0.01	5.20	0.62	AB082526	NEK9
A_23_P99063	0.01	0.01	0.35	0.04	NM_002345	LUM
A_23_P259189	0.91	0.08	11.73	0.98	NM_013943	CLIC4
A_23_P328298	0.01	0.01	0.13	0.01	NM_020729	ODF2L
A_23_P254888	0.87	0.06	23.13	1.64	NM_003461	ZYX
A_23_P212696	0.09	0.01	81.09	6.76	NM_007085	FSTL1
A_32_P74477	0.01	0.01	0.29	0.02		
A_32_P157391	0.01	0.01	0.25	0.03	NM_153696	
A_24_P131236	0.01	0.01	0.05	0.01	NM_014849	SV2A
A_23_P125423	0.14	0.01	5.77	0.72	NM_001733	C1R
A_32_P151823	0.04	0.01	11.84	1.30	BC040619	

A_23_P93938	0.01	0.01	0.48	0.04	AB002361	NACAD
A_23_P55251	0.98	0.07	13.34	0.94	NM_002204	ITGA3
A_23_P66525	0.01	0.01	1.18	0.13	NM_006042	HS3ST3A1
A_23_P341938	0.01	0.01	0.08	0.01	NM_005450	NOG
A_24_P293358	0.01	0.01	0.09	0.01	NM_199050	C21orf25
A_24_P845223	0.67	0.05	6.10	0.43	M27126	
A_24_P133017	0.01	0.01	0.80	0.09	NM_032780	TMEM25
A_23_P44964	0.16	0.02	4.38	0.44	NM_001010924	C10orf38
A_24_P305570	0.03	0.01	1.27	0.10	NM_018993	RIN2
A_32_P88987	0.01	0.01	0.16	0.01	AK022346	
A_32_P66222	0.06	0.01	0.56	0.04	NM_001012421	ANKRD20A2
A_23_P363472	0.15	0.01	1.33	0.09	AB032991	NDFIP2
A_24_P323395	0.03	0.01	0.52	0.05	NM_016441	CRIM1
A_32_P216507	0.01	0.01	0.29	0.02	AI024778	
A_32_P108156	0.01	0.01	0.26	0.02	NR_001458	
A_24_P327181	0.07	0.01	0.62	0.05	NM_032387	WNK4
A_23_P134946	0.71	0.05	6.52	0.46	NM_014665	LRRC14
A_23_P416395	0.10	0.01	0.78	0.06	NM_003714	STC2
A_23_P101905	0.24	0.03	7.01	0.69	NM_005883	APC2
A_24_P589266	0.01	0.01	0.35	0.04	AL831825	SH3MD4
A_32_P27479	0.01	0.01	0.29	0.02	NM_145007	NLRP11
A_23_P425917	0.01	0.01	0.10	0.01	NM_153711	FAM26E
A_24_P329795	0.06	0.01	0.65	0.05	NM_007021	C10orf10
A_23_P354314	0.01	0.01	0.33	0.04	NM_153832	GPR161
A_24_P406754	0.01	0.01	0.42	0.05	NM_032211	LOXL4
A_23_P145657	1.30	0.09	41.12	3.95	NM_012447	STAG3
A_32_P119869	0.01	0.01	0.24	0.02		
A_32_P748131	0.01	0.01	0.25	0.02	AB075828	ZNF545
A_23_P411761	0.01	0.01	0.27	0.02	NM_031205	CABP1
A_32_P141338	0.01	0.01	0.37	0.04	BE930053	ANXA1
A_23_P402892	0.01	0.01	0.10	0.01	NM_032206	NLRC5
A_23_P356581	1.50	0.12	17.21	1.46	NM_022370	ROBO3
A_32_P50603	0.12	0.01	2.79	0.23	AK091483	
A_23_P64404	1.43	0.10	8.91	0.63	NM_021727	FADS3
A_23_P34700	0.08	0.01	0.24	0.02	NM_000364	TNNT2
A_24_P323114	0.07	0.01	1.66	0.19	NR_001446	ANXA2P3
A_24_P943588	0.04	0.01	0.14	0.01	AF201385	TXNRD2
A_23_P153320	0.22	0.01	4.37	0.39	NM_000201	ICAM1
A_23_P351275	0.03	0.01	1.19	0.10	NM_181597	UPP1
A_32_P91821	0.01	0.01	0.22	0.02	BM041657	LDHB
A_23_P83094	0.04	0.01	5.02	0.68	NM_007005	TLE4
A_23_P331928	0.35	0.03	6.78	0.70	AK095888	CD109
A_32_P165477	0.01	0.01	1.76	0.12	BC041925	SLC7A11
A_23_P50349	0.59	0.05	6.40	0.53	NM_004240	TRIP10
A_32_P109242	0.01	0.01	0.17	0.02	AK055302	
A_23_P21485	0.01	0.01	0.87	0.08	NM_017933	PID1
A_23_P42116	4.39	0.31	13.68	0.97	NM_005155	PPT2
A_24_P221485	0.01	0.01	0.31	0.02		
A_23_P69863	0.01	0.01	0.45	0.05	NM_015669	PCDHB5
A_23_P101054	0.02	0.01	3.03	0.25	NM_021013	KRT34
A_32_P162880	0.01	0.01	0.14	0.01	AK092078	
A_23_P258912	0.01	0.01	0.99	0.09	NM_003970	MYOM2
A_23_P110712	0.66	0.05	6.48	0.52	NM_004417	DUSP1
A_23_P312246	0.01	0.01	0.91	0.11	NM_024725	CCDC82
A_23_P202004	0.48	0.04	4.34	0.38	NM_020200	PRTFDC1
A_23_P72668	0.01	0.01	0.33	0.03	NM_004657	SDPR
A_32_P201976	0.02	0.01	0.35	0.04	AI860683	
A_23_P63736	0.01	0.01	7.82	0.55	NM_032770	
A_24_P39195	0.01	0.01	0.23	0.02	NM_080818	OXGR1
A_24_P94916	0.01	0.01	0.15	0.01	NM_007161	LST1
A_23_P88069	0.08	0.01	3.52	0.48	NM_005780	LHFP
A_23_P54055	0.93	0.07	10.28	0.94	NM_032876	JUB
A_23_P22682	0.01	0.01	2.94	0.39	NM_016608	ARMCX1
A_23_P55666	0.01	0.01	0.21	0.02	AK023047	ZNF702
A_23_P213319	0.01	0.01	1.17	0.08	NM_197941	ADAMTS6
A_23_P39076	4.78	0.34	23.51	1.66	NM_006270	RRAS

A_23_P144126	0.01	0.01	0.47	0.05	NM_014375	FETUB
A_32_P55979	0.03	0.01	2.71	0.28		
A_23_P43864	0.01	0.01	1.55	0.20		GABRG3
A_32_P200238	0.09	0.01	0.56	0.04	CR603668	
A_24_P860781	0.64	0.05	10.51	0.74	AK092921	
A_23_P24135	0.23	0.02	0.83	0.06	NM_001057	TACR2
A_32_P86118	1.00	0.09	10.61	1.02	NM_001010853	ACY1L2
A_32_P28402	0.22	0.02	0.74	0.05	AL713754	
A_23_P359376	0.01	0.01	0.06	0.01	NR_001317	HCG4P6
A_23_P80759	0.01	0.01	1.47	0.18	BC017572	PVRL3
A_23_P101623	0.01	0.01	0.18	0.01	NM_022103	ZNF667
A_24_P43810	0.05	0.01	0.44	0.03	NM_207006	FAM83A
A_23_P151267	3.02	0.25	30.16	2.85	NM_016357	LIMA1
A_23_P17269	0.09	0.01	1.42	0.18	NM_018084	CCDC88A
A_32_P39049	0.52	0.04	1.53	0.11	AK055214	
A_23_P422667	0.01	0.01	0.15	0.01	NM_016102	TRIM17
A_24_P223124	0.67	0.06	6.85	0.65	NM_022763	FNDC3B
A_24_P383523	0.12	0.02	3.47	0.31	NM_015589	SAMD4A
A_32_P153195	0.04	0.01	0.85	0.08	AK056142	
A_23_P320225	0.01	0.01	0.15	0.01	NM_147127	EVC2
A_23_P28857	0.01	0.01	0.16	0.01	NM_080816	SIRPG
A_32_P140501	0.05	0.01	6.41	0.55		AXL
A_23_P21134	0.38	0.03	2.82	0.23	NM_004083	DDIT3
A_24_P580698	0.04	0.01	0.79	0.07		
A_24_P114739	0.28	0.03	4.32	0.45	NM_172127	CAMK2D
A_23_P108751	0.57	0.04	289.66	27.78	NM_201555	FHL2
A_32_P207986	0.06	0.01	0.41	0.03		
A_24_P940166	0.28	0.02	1.66	0.12	NM_001015880	PAPSS2
A_23_P15357	0.97	0.07	4.64	0.33	NM_005567	LGALS3BP
A_23_P160618	0.45	0.03	2.35	0.17	NM_003975	SH2D2A
A_23_P390139	0.01	0.01	0.11	0.01	NM_153015	TMEM74
A_24_P24848	0.01	0.01	0.21	0.02	NM_014068	PSORS1C1
A_24_P330303	0.26	0.02	2.16	0.17	NM_152330	FRMD6
A_23_P171296	0.08	0.01	1.85	0.20	NM_002436	MPP1
A_23_P79331	0.07	0.01	5.73	0.66	NM_022152	TMBIM1
A_23_P353005	0.01	0.01	0.20	0.02	NM_152553	IBRDC1
A_32_P184039	0.01	0.01	0.22	0.02		
A_24_P500621	0.60	0.04	4.99	0.40	AK074291	
A_24_P339126	0.01	0.01	18.52	1.56	NM_144957	PRSS21
A_24_P62505	0.06	0.01	0.37	0.03	NM_015101	GLT25D2
A_23_P147109	0.48	0.05	6.48	0.62	NM_017723	C9orf167
A_24_P67534	0.01	0.01	0.21	0.02	BC008642	
A_23_P352484	0.01	0.01	0.48	0.05	NM_152721	DOK6
A_24_P365469	1.82	0.13	11.57	0.82	NM_004776	B4GALT5
A_23_P106016	0.02	0.01	1.53	0.19	NM_002742	PRKD1
A_32_P154726	0.01	0.01	0.26	0.03		
A_24_P384969	0.01	0.01	0.30	0.02	BC015977	
A_23_P501634	0.02	0.01	0.10	0.01	NM_078476	BTN2A1
A_32_P122703	0.01	0.01	0.37	0.04	NM_173582	PGM2L1
A_24_P944154	0.01	0.01	0.30	0.03	AL832717	
A_24_P910169	0.99	0.07	2.03	0.14		
A_24_P479793	0.05	0.01	0.39	0.03		
A_23_P138168	0.01	0.01	9.10	1.24	NM_001839	CNN3
A_23_P107401	3.86	0.27	30.61	2.16	NM_003255	TIMP2
A_23_P52797	0.08	0.01	0.90	0.08	NM_030770	TMPRSS5
A_23_P138541	0.26	0.02	6.80	0.48	NM_003739	AKR1C3
A_23_P215525	0.01	0.01	2.56	0.34	NM_145323	OSBPL3
A_32_P89709	0.85	0.06	5.23	0.37	NM_001018004	TPM1
A_23_P55179	0.32	0.02	4.09	0.29	NM_001661	ARL4D
A_24_P315256	0.01	0.01	2.94	0.21		
A_23_P131846	0.63	0.05	3.93	0.28	NM_005985	SNAI1
A_23_P313389	1.53	0.14	16.65	1.51	NM_003358	UGCG
A_23_P129821	0.06	0.01	0.42	0.03	NM_021012	KCNJ12
A_23_P45955	0.03	0.01	0.41	0.04	NM_014466	TEKT2
A_23_P43684	0.01	0.01	0.67	0.06	NM_017637	BNC2
A_23_P258310	0.01	0.01	0.27	0.03	NM_144651	PXDNL

A_23_P157299	1.19	0.12	14.91	1.27	NM_001129	AEBP1
A_24_P186274	0.03	0.01	1.03	0.10	NM_001007563	IGFBPL1
A_23_P256470	0.02	0.01	2.23	0.16	NM_000905	NPY
A_24_P123833	0.01	0.01	0.30	0.03	NM_012431	SEMA3E
A_23_P54576	0.28	0.03	3.28	0.26	NM_005550	KIFC3
A_23_P125680	0.01	0.01	0.29	0.03	NM_017938	FAM70A
A_32_P108544	0.78	0.06	2.45	0.17	AY354203	RGPD1
A_24_P870620	0.01	0.01	1.48	0.19	NM_002825	PTN
A_23_P352957	0.16	0.02	2.58	0.27	NM_025090	USP36
A_24_P296808	0.01	0.01	10.68	1.12	NM_018215	
A_24_P673063	2.52	0.18	20.59	1.64	NM_001444	FABP5
A_23_P431569	0.38	0.03	2.37	0.17	BC033133	
A_24_P399220	0.01	0.01	1.46	0.16		
A_23_P209408	0.01	0.01	0.45	0.05	NM_032977	CASP10
A_23_P96350	2.39	0.17	9.06	0.64	NM_007213	PRAF2
A_23_P4536	0.01	0.01	3.43	0.47	NM_012307	EPB41L3
A_23_P323180	0.01	0.01	0.80	0.10	NM_006898	HOXD3
A_32_P218025	0.01	0.01	0.17	0.02	BG288911	
A_23_P215634	0.07	0.01	15.42	2.20	NM_001013398	IGFBP3
A_24_P226008	4.80	0.40	40.97	3.17	NM_007283	MGLL
A_23_P34433	0.01	0.01	1.24	0.16	NM_001009881	ZCCHC11
A_24_P933151	0.05	0.01	0.08	0.01	S69023	HOXB6
A_23_P370625	1.21	0.09	2.36	0.17	NM_020451	SEPN1
A_23_P131924	0.33	0.02	0.60	0.04	NM_000678	ADRA1D
A_24_P129417	0.15	0.01	0.29	0.02	NM_006131	BMP1
A_24_P924329	0.06	0.01	0.11	0.01		
A_23_P130169	0.93	0.07	1.51	0.11	NM_014726	TBKBP1
A_23_P65890	0.62	0.04	0.95	0.07	NM_006383	CIB2
A_32_P79719	7.83	0.55	10.41	0.74		
A_24_P931628	0.12	0.01	0.16	0.01	AY358705	
A_24_P227971	0.06	0.01	0.12	0.01	NM_016212	
A_24_P375132	4.30	0.30	6.03	0.43		
A_24_P460419	0.11	0.01	0.21	0.02		
A_32_P133526	0.04	0.01	0.07	0.01		
A_23_P218111	0.09	0.01	0.15	0.01	NM_001002236	SERPINA1
A_32_P44316	24.65	1.74	47.38	3.35	NM_001402	EEF1A1
A_24_P33213	31.42	2.22	43.75	3.09		
A_24_P61150	0.08	0.01	0.12	0.01	NM_002247	KCNMA1
A_32_P218493	3.93	0.28	7.41	0.52	BX113802	
A_24_P73408	3.78	0.27	5.26	0.37	NM_145293	
A_32_P178503	0.15	0.01	0.19	0.02	BC032064	
A_32_P192984	0.26	0.02	0.42	0.03	AK128396	
A_32_P127812	0.16	0.01	0.28	0.02	BX329117	
A_24_P231250	0.23	0.02	0.46	0.03	NM_020451	SEPN1
A_24_P941540	14.15	1.00	21.28	1.51		
A_23_P393627	0.56	0.04	1.12	0.08	NM_138693	KLF14
A_24_P13715	0.12	0.01	0.17	0.01	NM_147188	FBXO22
A_24_P363896	0.55	0.04	0.92	0.07	NM_032888	COL27A1
A_23_P17152	0.15	0.01	0.27	0.02		
