# Supplementary material for: An integrative genomic analysis revealed the relevance of microRNA and gene expression for drug-resistance in human breast cancer cells
Source: Mol Cancer. 2011 Nov 3;10:135. doi: 10.1186/1476-4598-10-135 (PMC3247093; doi:10.1186/1476-4598-10-135)
Supplement: Additional file 11 — Table S6. List of genes accosiated with apoptosis (KEGG web site). [file 1476-4598-10-135-S11.PDF]

Table S6 List of genes accosiated with apoptosis (KEGG web site)

ID	MCF7		MCF7-ADR			
Systematic	Normalized	StdErr Norm	Normalized	StdErr Norm	Genbank	Gene Symbol
A_23_P100111	0.14	0.01	0.23	0.02	NM_007236	
A_24_P111217	0.01	0.01	0.01	0.01	AF116689	
A_32_P199736	0.31	0.02	0.27	0.02	AA765447	
A_23_P424561	5.08	0.36	1.29	0.12	NM_133639	RHOV
A_23_P112801	41.17	2.91	43.98	4.22	NM_007236	
A_23_P92623	5.11	0.39	6.21	0.79	NM_000944	PPP3CA
A_24_P414371	5.82	0.47	7.42	0.97	NM_000944	PPP3CA
A_24_P282083	0.10	0.04	0.09	0.01	AK097085	
A_32_P12344	0.86	0.07	0.87	0.06	BU618279	PPP3CA
A_24_P350372	0.01	0.01	0.01	0.01	AF090942	
A_24_P98524	3.04	0.22	1.94	0.17	NM_021132	PPP3CB
A_24_P718672	0.03	0.01	0.04	0.01	AK024173	
A_23_P157495	2.30	0.16	2.34	0.38	NM_005605	PPP3CC
A_24_P388252	0.35	0.05	0.33	0.03	NM_000945	PPP3R1
A_23_P108592	0.06	0.01	0.03	0.02	NM_000945	PPP3R1
A_24_P149689	0.04	0.15	0.04	0.17	NM_147180	PPP3R2
A_23_P9348	0.02	0.01	0.01	0.01	NM_147180	PPP3R2
A_23_P208706	42.64	0.97	29.36	2.93	NM_138764	BAX
A_23_P346309	26.92	1.90	34.39	2.43	NM_138762	BAX
A_23_P346311	10.07	0.71	6.61	0.88	NM_138762	BAX
A_32_P215078	0.12	0.01	0.09	0.01	BF857817	BCL2L1
A_24_P100130	0.10	0.02	0.08	0.01	NM_001191	BCL2L1
A_23_P210886	0.97	0.06	0.81	0.02	NM_138578	BCL2L1
A_24_P222758	4.87	0.42	13.06	1.04	NM_004401	DFFA
A_23_P12189	5.74	0.45	16.32	1.67	NM_213566	DFFA
A_24_P512775	0.01	0.01	0.01	0.01	AU146963	
A_23_P352266	6.89	0.73	0.34	0.03	NM_000633	BCL2
A_23_P208132	4.56	0.32	3.39	0.41	NM_000657	BCL2
A_23_P374812	0.02	0.01	0.09	0.01	NM_138293	ATM
A_23_P35916	0.05	0.00	0.16	0.02	NM_000051	ATM
A_24_P103944	0.01	0.01	0.01	0.01	NM_138293	ATM
A_23_P203267	0.04	0.01	0.06	0.01	NM_012101	TRIM29
A_23_P340123	0.09	0.01	0.06	0.01	NM_058193	TRIM29
A_23_P26810	0.90	0.02	0.33	0.01	NM_000546	TP53
A_23_P83266	12.92	0.91	4.15	0.33	NM_004435	ENDOG
A_23_P72537	21.63	1.53	9.98	1.15	NM_004208	AIFM1
A_23_P92410	3.40	0.24	5.24	0.51	NM_004346	CASP3
A_24_P115774	0.67	0.08	1.46	0.22	NM_001166	BIRC2
A_23_P98350	0.01	0.01	0.10	0.02	NM_001165	BIRC3
A_23_P22460	0.35	0.04	0.17	0.02	NM_001167	BIRC4
A_24_P278684	0.01	0.01	0.01	0.01	NM_001004285	DFFB
A_24_P370626	0.08	0.01	0.09	0.01	NM_001004285	DFFB
A_23_P44546	0.29	0.03	0.25	0.02	NM_001004285	DFFB
A_23_P97309	0.94	0.07	1.33	0.14	NM_001229	CASP9
A_24_P111342	1.39	0.10	1.99	0.24	NM_001229	CASP9
A_23_P12572	3.12	0.22	1.29	0.20	NM_033339	CASP7
A_23_P150207	22.36	1.58	21.26	1.92	NM_004322	BAD
A_23_P30024	4.44	0.24	2.40	0.18	NM_003998	NFKB1
A_32_P180199	0.06	0.01	0.02	0.01	AW511634	NFKB1
A_23_P202156	0.34	0.01	0.47	0.01	NM_002502	NFKB2
A_24_P937256	0.04	0.01	0.04	0.01	U88316	
A_23_P104689	4.51	0.31	3.13	0.15	L19067	RELA
A_23_P500799	7.19	0.79	4.83	0.64	NM_001226	CASP6
A_24_P245322	1.61	0.12	1.26	0.10	NM_212472	PRKAR1A
A_24_P356592	0.84	0.06	0.84	0.10	NM_212472	PRKAR1A
A_32_P190097	0.29	0.04	0.30	0.05	AI467970	PRKAR1A
A_23_P433753	5.96	0.42	5.60	0.64	NM_212472	PRKAR1A
A_24_P216501	1.43	0.10	0.89	0.06	NM_002735	PRKAR1B
A_32_P72394	0.38	0.03	0.44	0.03	NM_002735	PRKAR1B
A_23_P83599	0.78	0.06	0.32	0.02	NM_002735	PRKAR1B
A_23_P22214	0.09	0.01	0.27	0.03	NM_004157	PRKAR2A
A_32_P142991	11.05	0.78	20.56	2.18	AK026351	
A_24_P943335	0.03	0.01	0.05	0.01	BC002763	PRKAR2A
A_32_P76882	0.01	0.01	0.01	0.01	AI720033	
A_23_P42975	0.77	0.08	0.19	0.02	NM_002736	PRKAR2B
A_32_P145477	0.19	0.09	0.02	0.02	BX350256	
A_24_P916496	0.27	0.02	17.21	2.10	NM_002737	PRKCA
A_23_P55099	0.03	0.03	0.60	0.07	NM_002737	PRKCA
A_24_P399630	2.41	0.18	1.30	0.09	NM_002730	PRKACA
A_24_P408206	0.13	0.01	0.16	0.02	NM_002730	PRKACA
A_24_P62708	0.12	0.01	0.16	0.03	NM_002731	PRKACB
A_23_P371410	0.02	0.01	0.09	0.01	NM_207578	PRKACB
A_23_P71926	0.01	0.01	0.01	0.01	NM_002732	PRKACG
A_23_P106002	40.96	2.90	13.49	1.58	NM_020529	NFKBIA
A_23_P36611	0.06	0.01	0.30	0.06	NM_181861	APAF1
A_24_P376556	5.12	0.36	13.52	1.90	NM_018947	CYCS
A_24_P29665	0.09	0.01	0.34	0.03	NM_018947	CYCS
A_32_P174083	0.35	0.03	1.83	0.20	NM_018947	CYCS
A_24_P187948	13.52	1.01	17.93	1.38	NM_197966	BID
A_23_P154929	2.30	0.18	1.43	0.17	NM_197966	BID
A_24_P157087	0.15	0.04	0.32	0.05	NM_033356	CASP8
A_24_P148499	0.01	0.01	0.01	0.01	NM_033358	CASP8
A_23_P209389	2.80	0.10	3.25	0.10	NM_033356	CASP8
A_24_P3045	0.01	0.01	0.06	0.01	NM_032974	CASP10

A_24_P139993	0.01	0.01	0.39	0.05	NM_032977	CASP10
A_23_P209408	0.01	0.01	0.45	0.05	NM_032977	CASP10
A_24_P544543	1.49	0.25	1.25	0.16	AK022319	CAPN1
A_23_P138835	7.05	0.50	2.76	0.20	NM_005186	CAPN1
A_23_P23924	1.78	0.15	88.67	6.85	NM_001748	CAPN2
A_24_P45481	0.01	0.01	0.04	0.01	NM_005465	AKT3
A_24_P110983	0.01	0.01	3.30	0.32	AK055109	AKT3
A_23_P160354	0.01	0.01	0.82	0.07	NM_181690	AKT3
A_23_P2960	2.18	0.11	0.55	0.05	NM_005163	AKT1
A_23_P373475	0.72	0.06	0.36	0.12	NM_001626	AKT2
A_24_P359267	130.66	9.24	52.48	5.64	BC022779	AKT2
A_24_P935345	0.05	0.01	0.04	0.02	AY358191	
A_23_P208870	15.07	1.07	5.63	0.50	BC022779	AKT2
A_24_P550562	0.01	0.01	0.01	0.01	AA420988	
A_23_P207319	1.03	0.07	4.04	0.31	NM_003954	MAP3K14
A_23_P46748	0.41	0.03	0.45	0.11	NM_001278	CHUK
A_24_P132518	1.12	0.09	1.76	0.24	NM_001556	IKBKB
A_23_P216188	1.57	0.11	2.35	0.28	NM_001556	IKBKB
A_24_P155058	0.14	0.02	0.19	0.03	NM_001556	IKBKB
A_23_P159920	3.50	0.25	1.93	0.14	NM_003639	IKBKG
A_24_P120115	0.26	0.03	0.43	0.04	NM_003879	CFLAR
A_23_P209394	1.69	0.12	5.31	0.50	U97075	CFLAR
A_23_P66543	0.02	0.01	0.02	0.01	NM_014308	PIK3R5
A_23_P66540	0.05	0.01	0.06	0.01	NM_014308	PIK3R5
A_23_P92057	0.32	0.04	0.78	0.11	NM_006218	PIK3CA
A_23_P346969	0.25	0.02	0.42	0.08	NM_006219	PIK3CB
A_24_P920264	0.01	0.01	0.01	0.01	Z36836	
A_24_P191067	7.75	0.58	11.44	1.16	NM_001009566	CLSTN1
A_23_P33303	0.05	0.02	0.47	0.04	NM_005026	PIK3CD
A_24_P71244	0.08	0.01	1.48	0.14	NM_005026	PIK3CD
A_23_P259833	0.01	0.01	0.21	0.03	NM_005026	PIK3CD
A_24_P751082	0.26	0.02	0.37	0.03		CLSTN1
A_23_P251359	0.01	0.01	0.01	0.01	NM_002649	PIK3CG
A_24_P937193	0.01	0.01	0.01	0.01	L26969	PIK3R1
A_23_P144980	0.03	0.01	0.02	0.01	NM_181523	PIK3R1
A_24_P29401	0.54	0.05	0.43	0.05	NM_181523	PIK3R1
A_32_P37704	0.03	0.01	0.02	0.01	AA042935	PIK3R2
A_23_P142361	0.85	0.06	0.18	0.01	NM_005027	PIK3R2
A_24_P156781	0.20	0.02	0.01	0.01	NM_003629	PIK3R3
A_23_P22970	0.66	0.19	0.07	0.01	NM_003629	PIK3R3
A_23_P162300	0.02	0.01	0.12	0.01	NM_007199	IRAK3
A_32_P188388	0.12	0.01	0.09	0.01	AI185185	
A_23_P73780	19.84	1.40	11.44	0.95	NM_001569	IRAK1
A_23_P80635	0.02	0.01	0.01	0.01	NM_001570	IRAK2
A_24_P158903	0.16	0.02	0.09	0.01	NM_016123	IRAK4
A_23_P13978	0.43	0.05	0.22	0.02	NM_016123	IRAK4
A_23_P92140	0.57	0.04	0.10	0.01	NM_002468	MYD88
A_23_P362659	4.15	0.29	0.59	0.04	NM_002468	MYD88
A_24_P278637	5.20	0.37	2.83	0.26	NM_003824	FADD
A_23_P86917	30.51	2.31	17.74	1.50	NM_003824	FADD
A_23_P54649	22.30	1.58	19.31	1.85	NM_153425	TRADD
A_23_P169331	7.71	0.54	8.10	0.68	NM_021138	TRAF2
A_23_P154306	1.51	0.11	1.66	0.32	NM_004180	TANK
A_24_P257108	0.31	0.02	0.34	0.04	NM_133484	TANK
A_23_P370005	1.21	0.09	1.50	0.15	NM_003804	RIPK1
A_23_P120899	0.06	0.01	0.06	0.01	NM_000395	CSF2RB
A_23_P253081	0.01	0.01	0.04	0.01	NM_002183	IL3RA
A_32_P217750	0.01	0.01	0.40	0.03	NM_002183	IL3RA
A_24_P265506	0.08	0.06	0.06	0.02	NM_002529	NTRK1
A_23_P34804	0.02	0.01	0.06	0.01	NM_002529	NTRK1
A_24_P200023	0.01	0.01	0.02	0.01	NM_000877	IL1R1
A_23_P68006	0.05	0.01	0.16	0.03	NM_000877	IL1R1
A_24_P180165	0.02	0.01	0.06	0.02	NM_002182	IL1RAP
A_23_P170857	0.10	0.01	0.97	0.09	NM_002182	IL1RAP
A_24_P148762	0.01	0.01	0.05	0.01	NM_002182	IL1RAP
A_23_P336554	0.03	0.01	0.10	0.01	NM_134470	IL1RAP
A_23_P139722	0.83	0.06	2.08	0.14	NM_001065	TNFRSF1A
A_24_P364363	4.87	0.34	9.01	0.80	NM_001065	TNFRSF1A
A_23_P95417	0.01	0.01	0.03	0.01	NM_003840	TNFRSF10D
A_23_P409723	0.04	0.01	0.02	0.01	BC021569	
A_23_P256724	0.01	0.01	0.01	0.01	NM_003841	TNFRSF10C
A_23_P169030	0.53	0.04	0.96	0.11	NM_003842	TNFRSF10B
A_24_P218265	4.49	0.32	6.12	0.50	NM_003842	TNFRSF10B
A_23_P255653	0.50	0.06	0.14	0.01	NM_003844	TNFRSF10A
A_23_P44132	4.52	0.32	1.23	0.09	NM_004104	FASN
A_23_P63896	0.06	0.00	0.14	0.01	NM_000043	FAS
A_23_P348831	0.01	0.01	0.01	0.01	NM_000588	IL3
A_23_P115190	0.02	0.01	0.03	0.02	NM_002506	NGFB
A_23_P72096	0.03	0.07	0.08	0.01	NM_000575	IL1A
A_23_P79518	0.01	0.00	0.46	0.04	NM_000576	IL1B
A_23_P376488	0.05	0.01	0.23	0.02	NM_000594	TNF
A_24_P50759	0.04	0.01	0.06	0.01	NM_000594	TNF
A_23_P121253	0.02	0.00	0.18	0.15	NM_003810	TNFSF10
A_32_P126023	0.01	0.01	0.01	0.01	AI376429	TNFSF10
A_23_P369815	0.03	0.05	0.01	0.00	NM_000639	FASLG
A_24_P54220	0.01	0.01	0.01	0.01	NM_000639	FASLG
